# Long-term Effects of Multimodal Treatment on Adult Attention-Deficit/Hyperactivity Disorder Symptoms: Follow-up Analysis of the COMPAS Trial

**DOI:** 10.1001/jamanetworkopen.2019.4980

**Published:** 2019-05-31

**Authors:** Alexandra P. Lam, Swantje Matthies, Erika Graf, Michael Colla, Christian Jacob, Esther Sobanski, Barbara Alm, Michael Rösler, Wolfgang Retz, Petra Retz-Junginger, Bernhard Kis, Mona Abdel-Hamid, Helge H. O. Müller, Caroline Lücke, Michael Huss, Thomas Jans, Mathias Berger, Ludger Tebartz van Elst, Alexandra Philipsen

**Affiliations:** 1 Department of Psychiatry and Psychotherapy, University of Bonn, Bonn, Germany; 2 Department of Psychiatry and Psychotherapy, Faculty of Medicine, University Medical Center Freiburg, University of Freiburg, Freiburg, Germany; 3 Clinical Trials Unit, Institute of Medical Biometry and Statistics, Faculty of Medicine, University Medical Center Freiburg, University of Freiburg, Freiburg, Germany; 4 Clinic and Polyclinic for Psychiatry and Psychotherapy, University of Rostock, Rostock, Germany; 5 Clinic for Psychiatry and Psychotherapy, Medius Clinic, Kirchheim, Germany; 6 Center of Mental Health, Department of Psychiatry, Psychosomatics, and Psychotherapy, University Hospital of Würzburg, Würzburg, Germany; 7 Department of Psychiatry and Psychotherapy, Central Institute of Mental Health, Clinical Faculty Mannheim, University of Heidelberg, Mannheim, Germany; 8 Department of Child and Adolescent Psychiatry and Psychotherapy, University Medicine Mainz, Mainz, Germany; 9 Institute for Forensic Psychology and Psychiatry, Saarland University Faculty of Medicine, Homburg/Saar, Germany; 10 Department of Psychiatry and Psychotherapy, University Medical Center Mainz, Mainz, Germany; 11 Department of Psychiatry and Psychotherapy, University Medical Center Göttingen, Göttingen, Germany; 12 Center of Mental Health, Department of Child and Adolescent Psychiatry, Psychosomatics, and Psychotherapy, University Hospital of Würzburg, Würzburg, Germany

## Abstract

**Importance:**

Knowledge about the long-term effects of multimodal treatment in adult attention-deficit/hyperactivity disorder (ADHD) is much needed.

**Objective:**

To evaluate the long-term efficacy of multimodal treatment for adult ADHD.

**Design, Setting, and Participants:**

This observer-masked, 1.5-year follow-up of the Comparison of Methylphenidate and Psychotherapy in Adult ADHD Study (COMPAS), a prospective, multicenter randomized clinical trial, compared cognitive behavioral group psychotherapy (GPT) with individual clinical management (CM) and methylphenidate (MPH) with placebo (2 × 2 factorial design). Recruitment started January 2007 and ended August 2010, and treatments were finalized in August 2011 with follow-up through March 2013. Overall, 433 adults with ADHD participated in the trial, and 256 (59.1%) participated in the follow-up assessment. Analysis began in November 2013 and was completed in February 2018.

**Interventions:**

After 1-year treatment with GPT or CM and MPH or placebo, no further treatment restrictions were imposed.

**Main Outcomes and Measures:**

The primary outcome was change in the observer-masked ADHD Index of Conners Adult ADHD Rating Scale score from baseline to follow-up. Secondary outcomes included further ADHD rating scale scores, observer-masked ratings of the Clinical Global Impression scale, and self-ratings of depression on the Beck Depression Inventory.

**Results:**

At follow-up, 256 of 433 randomized patients (baseline measured in 419 individuals) participated. Of the 256 patients participating in follow-up, the observer-masked ADHD Index of Conners Adult ADHD Rating Scale score was assessed for 251; the mean (SD) baseline age was 36.3 (10.1) years; 125 patients (49.8%) were men; and the sample was well-balanced with respect to prior randomization (GPT and MPH: 64 of 107; GPT and placebo: 67 of 109; CM and MPH: 70 of 110; and CM and placebo: 55 of 107). At baseline, the all-group mean ADHD Index of Conners Adult ADHD Rating Scale score was 20.6, which improved to adjusted means of 14.2 for the GPT arm and 14.7 for the CM arm at follow-up with no significant difference between groups (difference, −0.5; 95% CI, −1.9 to 0.9; *P* = .48). The adjusted mean decreased to 13.8 for the MPH arm and 15.2 for the placebo arm (difference, −1.4; 95% CI, −2.8 to −0.1; *P* = .04). As in the core study, MPH was associated with a larger reduction in symptoms than placebo at follow-up. These results remained unchanged when accounting for MPH intake at follow-up. Compared with participants in the CM arm, patients who participated in group psychotherapy were associated with less severe symptoms as measured by the self-reported ADHD Symptoms Total Score according to the *Diagnostic and Statistical Manual of Mental Disorders* (Fourth Edition) (*DSM*-*IV*) of Conners Adult ADHD Rating Scale (AMD, −2.1; 95% CI, −4.2 to −0.1; *P* = .04) and in the subscale of reducing pure hyperactive symptoms, measured via the Diagnostic Checklist for the diagnosis of ADHD in adults (AMD, −1.3; 95% CI, −2.8 to 0.1; *P* = .08). Regarding the Clinical Global Impression scale assessment of effectiveness, the difference between GPT and CM remained significant at follow-up (odds ratio, 1.63; 95% CI, 1.03-2.59; *P* = .04). No differences were found for any comparison concerning depression as measured with the Beck Depression Inventory.

**Conclusions and Relevance:**

Results from COMPAS demonstrate a maintained improvement in ADHD symptoms for adults 1.5 years after the end of a 52-week controlled multimodal treatment period. The results indicate that MPH treatment combined with GPT or CM provides a benefit lasting 1.5 years. Confirming the results of the core study, GPT was not associated with better results regarding the primary outcome compared with CM.

**Trial Registration:**

isrctn.org Identifier: ISRCTN54096201

## Key Points

**Question:**

What are the long-term results of multimodal treatment for adult attention-deficit/hyperactivity disorder (ADHD) when comparing cognitive behavioral group psychotherapy (GPT) with individual clinical management (CM) in combination with either methylphenidate or placebo?

**Findings:**

In this follow-up assessment of the Comparison of Methylphenidate and Psychotherapy in Adult ADHD Study (COMPAS), a multicenter randomized clinical trial, 256 adults participated in follow-up 1.5 years after the intervention ended. The severity of ADHD symptoms improved in all 4 prior treatment groups, with no significant difference found between GPT and CM, but methylphenidate was associated with a larger improvement in symptoms compared with placebo.

**Meaning:**

Results from the COMPAS trial demonstrate an improvement of ADHD symptoms over 1.5 years in adults with ADHD after 1 year of treatment with methylphenidate plus either GPT or CM.

## Teaser

This follow-up assessment of the Comparison of Methylphenidate and Psychotherapy in Adult ADHD Study (COMPAS), a multicenter randomized clinical trial, evaluates the long-term efficacy of multimodal treatment for attention-deficit/hyperactivity disorder (ADHD).

## Introduction

Although guidelines recommend multimodal treatments in attention-deficit/hyperactivity disorder (ADHD), evidence of long-lasting effects in adults is scarce.^1,2,3,4,5^ In particular, studies investigating long-term effectiveness of interventions combining psychotherapy and stimulant medication compared with interventions not including medication are hardly available.^4^ Owing to the paucity of long-term follow-up data, generalizability of results for cognitive behavioral group psychotherapy (GPT) combined with pharmacotherapy is very limited. Moreover, quality of evidence was evaluated as poor by a 2018 Cochrane review.^6^

To our knowledge, the Comparison of Methylphenidate and Psychotherapy Study (COMPAS) is the first, and so far largest, multicenter randomized clinical study that evaluates the effects of GPT compared with clinical management (CM) combined with methylphenidate (MPH) or placebo in adults with ADHD over a 1-year treatment period.^7,8,9^ To assess the long-term effects of multimodal treatments, participants in COMPAS were reexamined 1.5 years after the interventions were terminated.

In the core study, all treatment arms showed improvements in ADHD symptoms.^7^ Methylphenidate combined with GPT or CM yielded better results than placebo regarding the primary outcome (ADHD Index of Conners Adult Rating Scale [CAARS], long version, in German)^10,11,12^ after 12 weeks of intensive treatment and during the maintenance phase after 1 year. Group psychotherapy was significantly associated with better results on the Clinical Global Impression (CGI) scale compared with CM.^13^

The question of the long-term effects of MPH treatment is still open to debate. In natural settings, discontinuation rates in patients with ADHD are high.^14,15,16^ Nevertheless, there is some evidence indicating a potential lasting effect of MPH even after discontinuation in adults.^17^ Huss et al^18^ found a 6-month enduring effect of long-acting MPH on symptoms of ADHD after discontinuation.

Psychological interventions are part of comprehensive treatment programs for ADHD^4,5^; however, evidence of long-lasting effects is lacking.^6,19^ Given these considerations, this article analyzes long-term effects of multimodal treatments (GPT vs CM plus MPH or placebo) on ADHD symptoms in the 1.5-year follow-up of COMPAS.

## Methods

### Study Design and Participants

Comparison of Methylphenidate and Psychotherapy Study is a 3-step factorial, multicenter randomized clinical trial. The trial protocol and statistical analysis plan are available in Supplement 1. In eTable 1 in Supplement 2, the 2 × 2 factorial study design is illustrated. Methods, including lists of criteria for participation and instruments for assessment of eligibility according to the European Medicines Agency guidelines,^20^ as well as results of the core study (steps 1 and 2) have been reported previously.^7,8,9^

**Figure 1.**
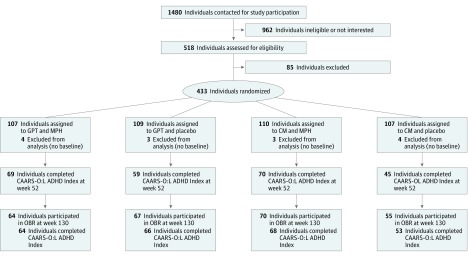
Study Design and Flow Diagram For information about participants excluded between week 0 and week 52, see original report of Comparison of Methylphenidate and Psychotherapy in Adult ADHD Study.^7^ CAARS-O:L ADHD Index indicates observer-rated Conners Adult ADHD Rating Scale ADHD Index, long version; CM, clinical management; GPT, group psychotherapy; MPH, methylphenidate; and OBR, observer-blinded ratings.

The core study comprised a 12-week intensive treatment followed by maintenance therapy over 9 months. Follow-up assessment (step 3) was conducted 2.5 years after baseline (Figure 1). The baseline represents the first assessment of primary and secondary end points after randomization (T1, week 0). It took place within 7 days of randomization. Written informed consent was obtained from all study participants before enrollment.

The COMPAS trial received approval from the leading ethics committee (Faculty of Medicine, Freiburg University) and local ethics committees at each study site. It was authorized by relevant German authorities (EudraCT No.: 2006-000222-31). This report follows the Consolidated Standards of Reporting Trials (CONSORT) reporting guideline.

#### Follow-up Procedure

Treatments ended after 52 weeks (T4).^7,8^ In the last sessions, continuation of psychotherapy and pharmacotherapy was discussed and advice provided. The follow-up visit at week 130 (T5) was included in the amendment of the trial protocol in 2009.

#### Outcome Measures Follow-up

Long-term efficacy was evaluated, including masked observer ratings and self-ratings of the CAARS (12 items, theoretical range, 0-36),^10,11,12^ the Diagnostic Checklist for the diagnosis of ADHD in adults (ADHD-DC,^21^ covering *Diagnostic and Statistical Manual of Mental Disorders* [Fourth Edition] [*DSM*-*IV*] criteria), and the CGI scale.^13^ The CGI scale comprises 3 global subscales (severity of illness, global improvement, and efficacy index) and assesses the global severity of illness and change in the clinical condition since baseline. To assess depressive symptoms, the Beck Depression Inventory (BDI-II)^22,23^ was used.

Treatment allocation, including follow-up assessment, was masked at all times for interviewing raters. Comparison of Methylphenidate and Psychotherapy Study was double-masked for medication and open with respect to assignment to GPT and CM for patients and therapists.

This report focuses on changes in the observer-rated ADHD Index of CAARS^10,11,12^ (CAARS-O:L) score from baseline (T1) to T5 and the stability of results from T4 (week 52) to T5. It further examines CAARS-O:L and self-ratings of CAARS (CAARS-S:L),^10,11,12^ ADHD-DC,^21^ CGI,^13^ and BDI-II.^22,23^

### Statistical Analysis

As in the core study, analyses were conducted according to randomized treatment in the full analysis set (FAS). The statistical analysis plan appears in Supplement 1. Rating scales of ADHD and depression were evaluated in analysis of covariance linear models including treatments (GPT vs CM and MPH vs placebo), center, and baseline measurements^24^ as fixed covariates. Regression-based within-group means at T5 were calculated at the corresponding all-group baseline mean in the FAS, ie, the mean at T1 across all groups, to account for random baseline imbalances and for dropout at T5. Response measurements (binary) and CGI measurements (ordinal) were analyzed in logistic and proportional odds models, respectively.

The primary focus of statistical analysis was on 2 × 2 comparisons of GPT vs CM and MPH vs placebo. Data description was done for the 4 randomized arms (GPT and MPH, GPT and placebo, CM and MPH, and CM and placebo; eTable 1 in Supplement 2). The primary CAARS ADHD Index was further explored by additional modified analyses: (1) 4-arm coding of treatment (GPT and MPH, GPT and placebo, CM and MPH, and CM and placebo), (2) additional inclusion of further prognostic baseline variables and of medication intake at T5, and (3) inclusion of interactive effects of potential baseline moderators and medication intake at T5 on ADHD symptoms. Changes from T4 to T5 were evaluated for both primary and major secondary outcomes (mixed-effects model for repeated measures). All *P* values were 2-tailed and considered to be significant in an exploratory sense if less than .05. Programming was performed using SAS version 9.2 (SAS Institute) in UNIX.

## Results

### Sample

Among 433 randomized patients, a baseline was obtained from 419, constituting the FAS of the core study; 256 of these individuals participated in observer-masked ratings at follow-up (details on enrollment in the article by Philipsen et al^8^). The CAARS-O:L ADHD Index^10,11,12^ was assessed at follow-up for 251 participants. Table 1 lists sociodemographic characteristics at baseline for these patients. The sample was well-balanced by sex (125 [49.8%] men; 126 [50.2%] women), age (range, 18-58 years; mean [SD] 36.3 [10.1]), and prior randomization (GPT and MPH: 64 of 107; GPT and placebo: 67 of 109; CM and MPH: 70 of 110; and CM and placebo: 55 of 107). Most (207 [82.5%]) had not taken MPH prior to randomization.

**Table 1.** Demographic Characteristics of 251 Participants With CAARS-O:L Index Scores at Follow-up by Randomized Interventions

Characteristic	No. (%)			
	GPT and MPH (n = 64)	GPT and Placebo (n = 66)	CM and MPH (n = 68)	CM and Placebo (n = 53)
Age, mean (SD) [range], y	35.7 (9.6) [19-57]	37.2 (11.2) [18-58]	36.5 (10.1) [18-54]	35.8 (9.7) [20-56]
Men	29 (45.3)	41 (62.1)	35 (51.5)	20 (37.7)
Verbal IQ, mean (SD) [range]	112.9 (14.7) [88-145]	112.2 (15.3) [89-143]	115.0 (13.9) [92-136]	111.1 (20.1) [23-145]^a^
White	62 (96.9)	66 (100)	68 (100)	52 (98.1)
University entrance diploma, y 5-12/13	29 (45.3)	30 (45.5)	42 (63.2)	26 (49.1)
Employment				
Full- or part-time	50 (84.7)	42 (68.9)	52 (83.9)	41 (82.0)
Unemployed	7 (11.9)	14 (23.0)	8 (12.9)	8 (16.0)
Family life				
≥2 Children	24 (37.5)	25 (37.9)	24 (35.3)	18 (33.9)
Single according to marital status	35 (54.7)	31 (47.0)	33 (48.5)	27 (50.9)
Living with a partner	28 (43.8)	33 (50.0)	26 (38.2)	30 (56.6)
Previous psychopharmacological treatments				
≥1 Previous psychopharmacological medication	27 (42.2)	33 (50.0)	35 (51.5)	25 (47.2)
Antidepressants	16 (25.0)	18 (27.3)	22 (32.4)	16 (30.2)
Methylphenidate, amphetamine, or other psychostimulants	15 (23.4)	18 (27.3)	13 (19.1)	9 (17.0)
Sedatives, neuroleptics, atomoxetine hydrochloride, mood stabilizers, or others	6 (9.4)	13 (19.7)	11 (16.2)	11 (20.8)
Previous psychiatric or psychotherapeutic treatments				
Outpatient^b^				
Psychiatric	21 (32.8)	20 (30.3)	22 (32.4)	22 (41.5)
Psychotherapeutic	37 (57.8)	32 (48.5)	39 (57.4)	26 (49.1)
Psychiatric and psychotherapeutic	13 (20.3)	10 (15.2)	15 (22.1)	13 (24.5)
Inpatient	12 (18.8)	13 (19.7)	16 (23.5)	10 (18.9)
WURS-k score, mean (SD)	40.5 (8.1)	41.6 (10.9)	41.9 (10.4)	41.6 (10.7)
CAARS-O:L Index score, mean (SD)	21.0 (5.3)	19.3 (6.2)	21.1 (5.2)	19.8 (4.4)
ADHD subtype				
Combined	41 (64.1)	32 (48.5)	37 (54.4)	31 (58.5)
Predominantly inattentive	23 (35.9)	31 (47.0)	26 (38.2)	18 (34.0)
Predominantly hyperactive-impulsive	0	3 (4.5)	5 (7.4)	4 (7.5)
Current comorbid Axis I disorder^b^^,^^c^				
≥1 Current clinical disorder	27 (42.2)	26 (39.4)	28 (41.2)	32 (60.4)
Affective disorders	18 (28.1)	18 (27.3)	19 (27.9)	26 (49.1)
Anxiety disorders	11 (17.2)	12 (18.2)	16 (23.5)	12 (22.6)
Other disorders	2 (3.1)	2 (3.0)	0	4 (7.5)
Current comorbid Axis II disorder^b^				
≥1 Current personality disorder	15 (23.4)	10 (15.2)	7 (10.3)	10 (18.9)
Cluster A: schizoid, paranoid	0	0	0	2 (3.8)
Cluster B: borderline, narcissistic, histrionic	3 (4.7)	2 (3.0)	3 (4.4)	2 (3.8)
Cluster C: avoidant, obsessive-compulsive, dependent	11 (17.2)	7 (10.6)	4 (5.9)	7 (13.2)
Other: depressive, negativistic, NOS	2 (3.1)	2 (3.0)	0	1 (1.9)

Abbreviations: ADHD, attention-deficit/hyperactivity disorder; CAARS-O:L, Conners Adult ADHD Rating Scale–Observer rating scale, long version in German; CM, clinical management; GPT, behavioral group psychotherapy; MPH, methylphenidate; NOS, not otherwise specified; WURS-k, Wender-Utah Rating Scale (in German).

^a^ IQ assessed with the Mehrfachwahl Wortschatz Intelligenztest. The IQ score of 23 was estimated because German was not the patient’s native language.

^b^ Multiple categories can apply.

^c^ Except nicotine dependency.

### Treatments After T4

After the study treatment ended, no further treatment restrictions were imposed. For final analysis, any medical or nonpharmacological intervention from T4 to T5 and at T5 was assessed (eTable 2 in Supplement 2). Overall, 23 patients (9.2%) took MPH intermittently (defined as MPH intake >31 days from T4 to T5 but not at T5). Current MPH intake at T5 was balanced among the 4 prior randomized arms with 78 of 251 (31.1%) taking MPH at follow-up. The mean (SD) daily dosage of MPH at T5 was 36.00 (24.77) mg and 0.46 (0.27) mg/kg of body weight (eTable 3 and eTable 4 in Supplement 2).

### Primary Outcome at T5

At baseline, the all-group mean ADHD Index of Conners Adult ADHD Rating Scale score was 20.6, which improved to adjusted means of 14.2 for the GPT arm and 14.7 for the CM arm at follow-up with no significant difference between groups (difference, −0.5; 95% CI, −1.9 to 0.9; *P* = .48). The adjusted mean decreased to 13.8 for the MPH arm and 15.2 for the placebo arm (difference, −1.4; 95% CI, −2.8 to −0.1; *P* = .04). A significant difference in the CAARS-O:L ADHD Index score at T5 was found between the group that was previously randomized to MPH compared with the group previously randomized to placebo (adjusted mean difference [AMD], −1.4; 95% CI, −2.8 to −0.1; *P* = .04), whereas the difference between GPT and CM was not significant (AMD, −0.5; 95% CI, −1.9 to 0.9; *P* = .48) (Table 2). See eTable 5 in Supplement 2 for further details. The results for the CAARS:O-L ADHD Index were virtually unchanged when current MPH intake was accounted for (GPT vs CM: AMD, −0.6; 95% CI, −1.9 to 0.8; *P* = .42; MPH vs placebo: AMD, −1.4; 95% CI, −2.8 to −0.1; *P* = .04), indicating stable overall effects of randomized GPT vs CM and MPH vs placebo on ADHD symptoms at follow-up.

**Table 2.** Observer-Rated Conners Adult ADHD Rating Scale (CAARS-O:L) Scores and Subscales

Group	T1, All-Group Mean	T5, Mean (95% CI)	T5 − T1, Mean (No. of Individuals)
**CAARS-O:L ADHD Index, Primary Outcome Scale, Range, 0-36**^a^			
GPT and MPH	20.6	13.7 (12.4 to 15.1)	−6.8 (64)
GPT and placebo	20.6	14.8 (13.4 to 16.1)	−5.8 (66)
CM and MPH	20.6	13.8 (12.5 to 15.2)	−6.7 (68)
CM and placebo	20.6	15.7 (14.2 to 17.2)	−4.9 (53)
GPT	20.6	14.2 (13.3 to 15.2)	−6.3 (130)
CM	20.6	14.7 (13.7 to 15.7)	−5.8 (121)
MPH	20.6	13.8 (12.8 to 14.7)	−6.8 (132)
Placebo	20.6	15.2 (14.2 to 16.2)	−5.4 (119)
Difference, GPT vs CM	NA	−0.5 (−1.9 to 0.9)	NA
*P* value	NA	.48	NA
Difference, MPH vs placebo	NA	−1.4 (−2.8 to −0.1)	NA
*P* value	NA	.04	NA
**CAARS-O:L Inattention/Memory Problems, Range, 0-36**^a^			
GPT	20.8	13.9 (12.9 to 15.0)	−6.9 (130)
CM	20.8	14.6 (13.6 to 15.7)	−6.2 (121)
MPH	20.8	13.8 (12.7 to 14.8)	−7.1 (132)
Placebo	20.8	14.8 (13.8 to 15.9)	−6.0 (119)
Difference, GPT vs CM	NA	−0.7 (−2.2 to 0.8)	NA
*P* value	NA	.34	NA
Difference, MPH vs placebo	NA	−1.1 (−2.5 to 0.4)	NA
*P* value	NA	.14	NA
**CAARS-O:L Hyperactivity/Restlessness, Range, 0-36**^a^			
GPT	18.3	12.6 (11.6 to 13.7)	−5.6 (130)
CM	18.3	14.1 (12.9 to 15.2)	−4.2 (121)
MPH	18.3	12.7 (11.7 to 13.8)	−5.5 (132)
Placebo	18.3	14.0 (12.9 to 15.1)	−4.3 (119)
Difference, GPT vs CM	NA	−1.4 (−2.9 to 0.1)	NA
*P* value	NA	.07	NA
Difference, MPH vs placebo	NA	−1.2 (−2.7 to 0.3)	NA
*P* value	NA	.11	NA
**CAARS-O:L Impulsivity and Emotional Lability, Range, 0-36**^a^			
GPT	18.6	12.7 (11.7 to 13.7)	−5.8 (130)
CM	18.6	13.5 (12.4 to 14.6)	−5.0 (121)
MPH	18.6	12.4 (11.4 to 13.5)	−6.1 (132)
Placebo	18.6	13.8 (12.8 to 14.9)	−4.7 (119)
Difference, GPT vs CM	NA	−0.8 (−2.2 to 0.7)	NA
*P* value	NA	.29	NA
Difference, MPH vs placebo	NA	−1.4 (−2.8 to 0.1)	NA
*P* value	NA	.06	NA
**CAARS-O:L Problems With Self-Concept, Range, 0-18**^a^			
GPT	9.9	7.7 (7.0 to 8.4)	−2.2 (130)
CM	9.9	8.0 (7.2 to 8.7)	−1.9 (121)
MPH	9.9	7.7 (7.0 to 8.4)	−2.2 (132)
Placebo	9.9	7.9 (7.2 to 8.7)	−1.9 (119)
Difference, GPT vs CM	NA	−0.3 (−1.3 to 0.7)	NA
*P* value	NA	.56	NA
Difference, MPH vs placebo	NA	−0.2 (−1.2 to 0.8)	NA
*P* value	NA	.65	NA
**Decrease in ADHD Index ≥30% Compared With T1, No./Total No. (%)**			
GPT	NA	65/130 (50.0)	NA
CM	NA	56/121 (42.3)	NA
MPH	NA	67/132 (50.8)	NA
Placebo	NA	54/119 (45.4)	NA
GPT vs CM, OR (95% CI)^a^^,^^b^	NA	1.26 (0.75 to 2.12)	NA
*P* value	NA	.38	NA
MPH vs placebo, OR (95% CI)^b^	NA	1.09 (0.65 to 1.83)	NA
*P* value	NA	.74	NA

Abbreviations: ADHD, attention-deficit/hyperactivity disorder; CM, clinical management; GPT, behavioral group psychotherapy; MPH, methylphenidate; NA, not applicable; T1, week 0; T5, week 130.

^a^ Regression analysis adjusted for baseline and center (least squares means from linear regression for CAARS-O:L scores). Lower score values represent better outcomes.

^b^ Odds ratios from logistic regression for CAARS-O:L response. Lower score values represent better outcomes. An odds ratio greater than 1 indicates higher odds for a better outcome for the first vs second intervention.

#### Interaction Between Randomized Medication and MPH at T5

To explore how the CAARS ADHD Index was associated with MPH at T5 and randomized study medication, a further model including an interaction was calculated. The lowest mean CAARS ADHD Index score was found for patients taking MPH at T5 who had previously taken MPH (mean score, 12.3). It was higher for patients previously randomized to the MPH arm but not taking MPH at T5 (mean score, 14.5) as well as for patients previously randomized to the placebo arm who were currently taking MPH (mean score, 14.7). However, it was highest in the former placebo group not treated with MPH at T5 (mean score, 15.4). Thus, the benefit of randomized MPH over placebo at follow-up was numerically larger for those who were taking MPH at T5 compared with those who were not. However, these effects of randomized medication on ADHD symptoms for the 2 groups with or without MPH at T5 as well as the difference between the effects were not statistically significant (MPH at T5 for MPH vs placebo: AMD, −2.4; 95% CI, −4.8 to 0.0; *P* = .06; no MPH at T5 for MPH vs placebo: AMD, −1.0; 95% CI, −2.6 to 0.7; *P* = .25; *P* value for interaction = .35). The mean difference in the CAARS ADHD Index between those taking MPH at T5 and those not taking it was numerically greater for those previously randomized to MPH compared with those randomized to placebo, reaching statistical significance for the MPH group (randomized to MPH with MPH at T5 vs randomized to MPH without MPH at T5: AMD, −2.2; 95% CI, −4.2 to −0.2; *P* = .03; randomized to placebo with MPH at T5 vs randomized to placebo without MPH at T5: AMD, −0.8; 95% CI, −3.0 to 1.4; *P* = .48).

#### Accounting for Antidepressants at T5

In an additional analysis, antidepressant intake at T5 was added to the initial model. Patients taking antidepressants scored 2.1 points (95% CI, 0.2-4.0) higher on the CAARS ADHD Index than patients not taking antidepressants (*P* = .03). The effect estimate of MPH vs placebo and GPT vs CM remained unchanged after adjustment.

#### Stability of Primary Outcome from T4 to T5

**Figure 2.**
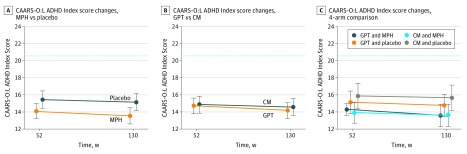
Attention-Deficit/Hyperactivity Disorder (ADHD) Index Score Changes Score changes from end of treatment (week 52) to follow-up (week 130). Least squares means with 95% CIs from longitudinal linear model. Dotted lines represent all-group baseline mean. CAARS-O:L ADHD Index indicates observer-rated Conners Adult ADHD Rating Scale ADHD Index, long version; CM, clinical management; GPT, group psychotherapy; and MPH, methylphenidate.

The stability of the primary outcome scale from T4 to T5 was analyzed in a longitudinal linear model (eTable 6 in Supplement 2). The mean differences between T5 and T4 CAARS-O:L ADHD Index scores revealed a nonsignificant decline in all study groups (GPT: AMD, −0.6; 95% CI, −1.5 to 0.4; *P* = .26; CM: AMD, −0.3; 95% CI, −1.3 to 0.8; *P* = .63; MPH: AMD, −0.5; 95% CI, −1.5 to 0.4; *P* = .28; placebo: AMD, −0.3; 95% CI, −1.4 to 0.8; *P* = .60). The results are depicted in Figure 2. Conclusions regarding between–treatment arm comparisons at follow-up were the same as previously seen in the core study at T4.^7^

### Secondary Outcomes

Secondary outcomes at T5 describing ADHD symptoms, which were measured with CAARS-O:L^10,11,12^ and CAARS-S:L^10,11,12^ as well as ADHD-DC,^21^ BDI-II,^22,23^ and CGI^13^ are listed in Table 2 and Table 3 as well as eTable 7 in Supplement 2. Changes for major secondary outcomes from T4 to T5 are listed in eTable 6 in Supplement 2. We found participants in the GPT arm scored significantly better on self-rated *DSM*-*IV* ADHD Symptoms Total of CAARS compared with participants in the CM arm (AMD, −2.1; 95% CI, −4.2 to −0.1; *P* = .04). The CAARS-S:L ADHD Index (eTable 7 in Supplement 2) and CAARS-O:L subscales (Table 2) revealed no significant results. In the ADHD-DC,^21^ GPT was significantly associated with reducing pure hyperactive symptoms at T4^7^ and T5 compared with CM (AMD, −1.3; 95% CI, −2.8 to 0.1; *P* = .08) (eTable 8 in Supplement 2). Concerning depression as measured with the BDI-II,^22,23^ no differences were found for any comparison (eTable 7 in Supplement 2). In the former GPT group, the mean BDI-II score significantly increased by 1.3 points (95% CI, 0.1 to 2.4; *P* = .03) from T4 to T5 (eTable 6 in Supplement 2). In the core study, CGI global assessment of effectiveness favored GPT over CM and MPH over placebo.^7^ The difference between GPT and CM was significant at T4^7^ and remained significant at follow-up (odds ratio, 1.63; 95% CI, 1.03-2.59; *P* = .04) (Table 3). Furthermore, with respect to CGI severity, a significant benefit of MPH over placebo at T5 was found.

**Table 3.** Clinical Global Impression (CGI) Scale–Observer Ratings at Follow-up by Randomized Intervention

Measure	T5, Mean (No.)^a^
**CGI Severity**^b^	
GPT	3.4 (128)
CM	3.5 (121)
MPH	3.2 (131)
Placebo	3.7 (118)
GPT vs CM, OR (95% CI)^c^	0.81 (0.51-1.28)
*P* value	.37
MPH vs placebo, OR (95% CI)^c^	0.47 (0.30-0.74)
*P* value	.001
**CGI Global Change**^d^	
GPT	2.7 (129)
CM	2.9 (122)
MPH	2.8 (132)
Placebo	2.8 (119)
GPT vs CM, OR (95% CI)^b^	0.76 (0.48-1.21)
*P* value	.25
MPH vs placebo, OR (95% CI)^b^	0.90 (0.57-1.42)
*P* value	.65
**CGI Global Assessment of Effectiveness**^e^	
GPT	2.5 (129)
CM	2.2 (122)
MPH	2.4 (132)
Placebo	2.2 (119)
GPT vs CM, OR (95% CI)^f^	1.63 (1.03-2.59)
*P* value	.04
MPH vs placebo, OR (95% CI)^f^	1.55 (0.99-2.44)
*P* value	.06

Abbrevations: CM, clinical management; GPT, group psychotherapy; MPH, methylphenidate; OR, odds ratio; T5, week 130.

^a^ Descriptive numerical evaluation.

^b^ Range for CGI Severity, 1 to 7, in which 1 indicates not at all ill and 7 indicates extremely ill.

^c^ An OR less than 1 indicates higher odds of a better outcome for the first vs second intervention.

^d^ Range for CGI Global Change, 1 to 7, in which 1 indicates very much improved and 7 indicates very much worse.

^e^ Range for CGI Global Assessment of Effectiveness, 1 to 4, in which 1 indicates minimally effectivene and 4 indicates very effective.

^f^ An OR greater than 1 indicates higher odds of a better outcome for the first vs second intervention.

### Prognostic Factors and Moderators

Age at baseline as a prognostic factor for outcomes at T5 showed no statistical significance. Age at baseline, sex, educational level, ADHD subtype, comorbidities, and severity at baseline were studied as potential moderators for outcomes at T5. However, no statistically significant effects were found for any of these (data not shown).

## Discussion

To our knowledge, COMPAS is the first trial to examine long-term multimodal treatment effects on adult ADHD symptoms, following the highest methodological standards for diagnostic, therapeutic, and assessment procedures.^7,9^ Our study revealed several promising new findings. First, irrespective of combined treatment (MPH vs placebo and GPT vs CM), we found stable improvements for ADHD symptoms and general functioning 1.5 years after a structured 52-week randomized clinical trial. Second, our results show that both specific (GPT) and unspecific (CM) psychological interventions were significantly better when combined with MPH vs placebo. Third, 1.5 years after treatment finalization, we found that patients who were randomized to the MPH arm during the study period scored significantly better on the primary outcome CAARS-O:L ADHD Index compared with patients randomized to the placebo arm. This indicates the longest-lasting effect on ADHD symptoms observed for MPH after dosage discontinuation. Our results remained stable even after controlling for effects of current antidepressants and/or current MPH intake at follow-up.

To investigate long-lasting effects of MPH on ADHD symptoms, the analysis put a certain emphasis on changes of observer and self-rating scales from T1 to T5 and the stability of results from T4 to T5. Analysis of changes from T4 to T5 for the CAARS-O:L ADHD Index revealed minor but insignificant declines for all study groups, indicating stable outcomes over a 1.5-year period.

At first glance, the long-lasting effects of MPH combined with GPT or CM on ADHD symptoms might be surprising. Methylphenidate has a short half-life and, depending on formulation, a maximum effective action of 7 to 12 hours.^25^ Its clinical effectiveness is thought to stop quickly after elimination.^25^ Explanation for long-lasting effects of multimodal treatment with MPH on ADHD symptoms may lie in neuroplasticity associated with learning processes^26^ as well as in coping strategies acquired during medication that outlast discontinuation. Patients treated with MPH might benefit more from GPT and CM even if the latter does not specifically address ADHD-related issues. Given that only 78 of 251 patients (31.1%) of the present sample took MPH at T5, it is conceivable that MPH acts as a sort of catalyzer and enables individuals to acquire new skills that allow long-lasting improvements of ADHD symptoms that supersede a continued treatment.

### Explanations for Long-lasting Effects of MPH

Evidence from animal research revealed the influence of MPH on neuronal remodeling in several brain regions.^27,28,29,30^ Moreover, changes in the neuroglial network,^28^ such as an increase in dendritic spine density, have been demonstrated.^31^ In the short term, MPH led to striatal gene expression changes in adolescent rats.^27^ The dopaminergic neurotransmission pathway is regarded as being crucially associated with ADHD,^32,33,34^ and MPH leads to augmented striatal dopamine availability.^34^ Continuous dopamine transporter blockade of MPH can lead to alterations in the brain, as a 2012 meta-analysis^35^ found an increased density of striatal dopamine transporters in patients who were previously treated with MPH as well as a lower density for drug-naive patients. Moreover, MPH is supposed to downregulate dopamine turnover in children and adolescents with ADHD.^36^ Therefore, the number of individuals previously treated with MPH has to be considered as an influencing factor for subsequent medical treatments. In our follow-up sample, most (207 [82.5%]) had been untreated with MPH prior to randomization.

Potential upregulation of dopamine transporters after long-term MPH treatment is discussed to be associated with an increase in ADHD symptoms while not taking medication.^37^ However, in our study, ADHD symptoms remained improved and stable for 1.5 years after termination of controlled treatment.

Beyond upregulation or downregulation of dopamine receptors, multiple components, such as the Wnt signal transduction pathways, seem to play a role in the long-term mechanisms of MPH.^38^ However, the cellular and molecular mechanisms influenced by MPH are still not completely understood, and further research is required. Nevertheless, the perception of potential adaptive brain changes after long-term MPH treatment is in line with MPH-associated effects on brain function^39^ and structure,^40^ which might be an explanation for long-term efficacy. Meta-analyses^40,41^ and neuroimaging studies^42^ reveal the association of MPH and age with normalization of brain structure anomalies, such as volume reductions, with smaller structural brain differences between individuals with and without ADHD, in which more participants were treated with psychostimulants. An association of psychostimulants with subcortical volume normalization has not been verified by a 2017 meta-analysis of cross-sectional observational data^43^ and a 2014 longitudinal study in children.^44^ In structural magnetic resonance imaging examinations linked to COMPAS, there was no evidence of gray matter volume loss; however, a trend toward cerebellar gray volume gains after 1 year of MPH treatment was found.^45^

Furthermore, data from 2017^46^ suggest that dose optimization in stimulant medication, which is recommended by several guidelines^4,47^ and performed in COMPAS, may enhance efficacy and safety. Moreover, there is evidence of a positive association of medical treatment duration with benefit in ADHD.^48,49^ However, the direct effects of individual dosage, treatment duration, and lasting effects on ADHD symptoms need to be clarified through future studies.

### Influence of MPH Intake at T5

Unlike other studies we are aware of that also investigated long-term effects of MPH on ADHD symptoms,^17,18^ COMPAS participants were evaluated under real-life conditions. Although MPH had not been approved as standardized therapy for adult ADHD in Germany until 2014, all participants were offered the opportunity to be transferred to specialists who would prescribe MPH off-label after T4. Nevertheless, the percentage of patients taking MPH at follow-up was only one-third and balanced between the 4 treatment groups. Furthermore, the rate of patients having individual psychological interventions after the core study can be regarded as low. All these results may indicate that patients felt sufficiently treated after the core study; this agrees with the previously reported moderate to high treatment satisfaction.^50^

The significant association of MPH at T5 with the CAARS-O:L ADHD Index in the group randomized to MPH may indicate an MPH-linked long-lasting neuromodulation effect in a multimodal setting. In contrast, patients taking MPH at T5 without an MPH pretreatment period (ie, randomized to the placebo group) had improved CAARS ADHD Index scores as well but without statistical significance.

### Long-term Effects of Psychological Interventions

Current evidence on follow-up assessments of cognitive behavioral therapy (CBT) in adults with ADHD is limited in terms of sample size and shorter follow-up periods compared with COMPAS.^6,51,52,53,54,55,56^ A 2018 meta-analysis on long-term efficacy of psychosocial treatments for adults with ADHD^57^ found evidence of sustained effects of ADHD-specific psychosocial treatment approaches on ADHD symptoms for at least 12 months.

In line with the main outcome of COMPAS, GPT showed no significant improvement compared with CM regarding the primary observer-rated outcome at follow-up. The better performance of participants in the GPT arm compared with participants in the CM arm for the self-rated *DSM*-*IV* ADHD Symptoms Total Score can be interpreted to be closely linked to the result that treatment with structured GPT was generally assessed to be more effective by patients (measured with the CGI assessment of effectiveness) compared with nonspecific counseling.^50^ This agrees with results in psychotherapy research for other disorders, such as depression.^58^

At follow-up, CGI global assessment of effectiveness again favored GPT over CM. Improvements in the CGI scale were also reported in patients with ADHD as an effect of CBT in combination with medication compared with medication alone by Safren et al^59,60^ as well as in a 3-month follow-up after CBT in patients taking medication by Emilsson et al.^51^ These findings indicate that patients’ individual symptom assessment and functional improvement after psychosocial interventions might differ from observer-rated symptom severity in the long term.^57^

For reducing pure hyperactive symptoms, our long-term results indicate that GPT is associated with larger improvement than CM, which again is in line with the results of Lopez et al,^6^ who reported an increase of self-rated treatment effects on the hyperactivity/impulsivity domain at follow-up. A reason for this may be that GPT led to a higher level of self-awareness and concurrent self-control or to more acceptance of hyperactivity as a part of ADHD symptoms, which in turn facilitates the implementation of coping strategies.^61^

### Limitations

This study had limitations. Although a large sample of COMPAS participants (>250) was recruited for follow-up assessment, a considerable number of patients (177 [40.9%]) failed to participate. Nevertheless, psychosocial and clinical baseline characteristics of the subsample assessed were descriptively similar to those of the baseline participants.^8^ The differences between the 4 treatment approaches studied are rather marginal. All 4 interventions resulted in a sound improvement for both the short and long run. Daily life functioning was not measured apart from the CGI scale.^62^ Also, we compared a highly structured group (GPT) with a less specific individual therapy (CM). On the one hand, CM was chosen as an active, nonpharmacological control condition to simulate general practice, representing a so-far insufficiently investigated area. On the other hand, there is evidence that individual therapy settings are more effective than group treatment^57^; thus, the presence of nonspecific factors derived from the different modalities could be a confounding variable.

## Conclusions

In COMPAS, a 1.5-year lasting improvement of ADHD symptoms in adult patients with ADHD following 1 year of multimodal treatment was found, with a significant effect of MPH on ADHD symptoms 1.5 years after discontinuation after a 1-year controlled intake period in a multimodal setting. Our results confirm the requirement for reevaluation of MPH treatment during medication-free periods, as proposed by the National Institute for Health and Care Excellence guidelines. However, further trials are needed to investigate the causes of long-lasting effects of multimodal treatments and treatment components on ADHD symptoms. Especially adaptive brain response to MPH in randomized clinical trials with longitudinal functional imaging studies is required to clarify final brain modifications.^35^

## Notes

Supplement 1. **Trial Protocol and Statistical Analysis Plan**

Supplement 2. **eTable 1.** 2 × 2 Factorial Study Design **eTable 2.** Treatments After T4 by Former Randomized Treatment Groups **eTable 3.** Daily Dosage MPH (mg) at T5 by Former Randomized Treatment Groups **eTable 4.** Daily Dosage MPH (mg/kg) at T5 by Former Randomized Treatment Groups **eTable 5.** Primary and Secondary Outcomes at 2.5 Years (T5) **eTable 6.** Stability T5-T4 **eTable 7.** Self-Rated CAARS ADHD Index and BDI Total Score **eTable 8.** Major Secondary Outcomes in the Full Analysis Set at Follow-Up (T5) 2.5 Years After Baseline (T1)

Supplement 3. **Data Sharing Statement**
