# Supplementary material for: Long-term Effects of Multimodal Treatment on Adult Attention-Deficit/Hyperactivity Disorder Symptoms: Follow-up Analysis of the COMPAS Trial
Source: JAMA Netw Open. 2019 May 31;2(5):e194980. doi: 10.1001/jamanetworkopen.2019.4980 (PMC6547099; doi:10.1001/jamanetworkopen.2019.4980)
Supplement: Supplement 1. — Trial Protocol and Statistical Analysis Plan [file jamanetwopen-2-e194980-s001.pdf]

Trial Protocol - Synopsis (original version in German, translated for JAMA Network Open)

Title: Effects and mechanisms of psychotherapy in the treatment of attention deficit hyperactivity disorder in adults: the first randomized multicenter study

Study-No.: 070170 EudraCT Number: 2006-000222-31
Trial Registration: Current Controlled Trials: ISRCTN54096201
Test product: Methylphenidate hydrochloride (brand preparation: Medikinet retard (10mg) or Placebo capsules à 10mg
Phase: Phase III

Study centers:

- Department of Psychiatry and Psychotherapy, Medical Center - University of Freiburg, Faculty of Medicine, University of Freiburg, Freiburg, Germany. Department of Psychiatry and Psychotherapy, Central Institute of Mental Health, Clinical Faculty Mannheim
- Institute for Forensic Psychology and Psychiatry, Saarland University, Faculty of Medicine, Homburg/Saar, Germany
- Department of Psychiatry and Psychotherapy, Campus Benjamin Franklin, Charité–University Medicine Berlin, Berlin, Germany
- Department of Psychiatry and Psychotherapy, Julius-Maximilians University Hospital of Würzburg, Würzburg, Germany
- Clinic and Polyclinic for Psychiatry and Psychotherapy, University of Rostock, Rostock, Germany (this center did not recruit any patients)
- Department of Psychiatry and Psychotherapy, LVR-Hospital Essen, Faculty of Medicine, University of Duisburg-Essen, Duisburg and Essen, Germany
- Department of Psychiatry and Psychotherapy, University Medical Center Mainz, Mainz, Germany and Department of Child and Adolescent Psychiatry and Psychotherapy, University Medicine Mainz, Mainz, Germany

Objectives:

- Investigation of the efficacy of ADHD-specific psychotherapy in the treatment of adult ADHD compared to unspecific clinical management, with or without concomitant pharmacotherapy
- Investigation of the combined effects of specific psychotherapy and methylphenidate treatment in comparison to pharmacological or psychotherapeutic treatment in combination with unspecific clinical management / placebo.

Methodology:
Study Design:
Randomized controlled, placebo-controlled, double-blind, multicenter trial (4-arm, 2x2 factorial)

Criteria for evaluation:

Primary endpoint:

Change of the ADHD severity, measured by the ADHD Index of the Conners' Adult Rating Scale assessed by blind observer rating (CAARS-O:L, 66 questions) between T1 (baseline) and T2 after 13 weeks (intensive treatment period).

Secondary endpoints:

- Conners' adult ADHD rating scale, observer-rated (CAARS-O:L) at study time points T3 (26 weeks), T4 (52 weeks), and T5 (1.5 years follow-up, 130 weeks)
- Conners' adult ADHD rating scale, self-rated, long version, CAARS-S:L
- ADHD-checklist (ADHD-DC, 18 questions), observer-rated
- Symptom-checklist–SCL-90-R, self-rated
- Beck Depression Inventory (BDI-II), self-rated
- Quality of Life Enjoyment and Satisfaction Questionnaire (Q-LES-Q), self-rated
- Clinical Global Impression Scale (CGI), observer-rated
- Subjective, retrospective effectiveness (group psychotherapy vs. unspecific clinical management), self-rated
- Nicotine and caffeine consumption, observer rated based on patient's report
- Missed days of work/education/training, clinician rated (blinded for medication, not blinded for group psychotherapy versus unspecific clinical management) based on patient's report
- Non-substance-related addictions, self-rated
- Specific socio-economic questionnaire (including SF-36 V2.0, self-rated, EQ-5D-3L, observer-rated) at T5
- Identifying clinical, neurobiological or genetic predictors for a treatment outcome of the different treatment conditions through closely linked neuroimaging and genetics
- Further exploratory analysis planed

Safety:

Safety data were continuously collected, documented and coded according to the Medical Dictionary for Regulatory Activities (MedDRA®) during week 0-52.

Treatment:

Patients are randomized to ADHD-specific group psychotherapy (GPT) or unspecific clinical management (CM), in combination with methylphenidate (MPH) or placebo (Plac) in a 2x2 factorial design. Each patient is allocated to one pharmacological treatment (MPH or Plac) and one non-pharmacological treatment (GPT or CM).

The duration of the interventional treatment phase is 52 weeks. GPT/CM is performed weekly for the first 12 weeks and monthly thereafter. Efficacy measurements are performed after 13, 26 and 52 weeks.

After treatment completion at 52 weeks, patients individually choose whether to continue pharmacological treatment. A follow-up measurement will be performed 1.5 years after completion of the interventional phase.

GPT is applied in the form of a structured ADHD-specific group-therapy program according to the manual of Hesslinger, incorporating methods of dialectic behavioural therapy and cognitive behavioural therapy (Hesslinger B, Philipsen A, Richter H: Psychotherapie der ADHS im Erwachsenenalter: ein Arbeitsbuch: Hogrefe Verlag; 2004). Each session lasts 90 minutes.
CM is performed in a one-on-one setting. Patients receive individual, supportive, non-directive counselling. Sessions last a maximum of 20 minutes.
Pharmacological treatment consists of MPH (preparation: Medikinet retard) or matching placebo. Patients are individually up-titrated from a start dose of 10 mg/day.
Pharmacological dosing and surveillance is performed at the same appointments as the GPT and CM sessions.

Test product, dose and mode of administration:

a) Trade name: Medikinet retard 10mg, sustained release,
   Product name: Methylphenidathydrochlorid, capsules, oral administration.
   Starting dose: 10 mg/day.
   Individual up-titration of dosage in the first 6 weeks with 10mg/week depending on effect and adverse events.
   Further individual up-titration is possible to a daily dosage of 1.3 mg/kg body weight at maximum. MPH dosage can be adjusted during the total 52-week period of trial participation. Medication adherence is assessed by pill count, supplemented by patients' comments on lost or forgotten blisters.

b) Placebo, capsules, oral administration.

Duration of treatment:
52 weeks

Study population:
500 patients will be assessed for eligibility, and 448 will be randomized. Patients are predominantly recruited out of the clinical sample referred for diagnosis and treatment to the units of psychiatry and psychotherapy of the study centers. It is estimated that 10% will not fulfil the full inclusion criteria. Therefore 448 patients will be included in the trial after the screening procedure. A dropout rate of 20% is assumed, a sample size of 112 per arm seems reasonable.

Inclusion criteria:

- ADHD according to DSM-IV criteria
- More than 30 points on the short version of the Wender-Utah-Rating-Scale
- Chronic course of ADHD symptoms from childhood to adulthood
- Male or female
- Age 18–60 years
- No clinically significant abnormality detected on physical examination, routine blood testing (blood count, renal, hepatic and thyroid function), ECG and EEG

- Written informed consent obtained.
- Participation in the baseline assessment within 7 days after randomization and treatment has to be started not later than 14 days after randomization.

Exclusion criteria:
- Mental handicap, severe learning disability, IQ<85
- Schizophrenia, bipolar disorder, borderline personality disorder, antisocial personality disorder, suicidal or self-injurious behaviour, autism, motor tics, Tourette's syndrome
- Substance abuse/dependence within 6 months prior to screening. Episodic abuse is not an exclusion criterion. Positive drug screening
- Neurological diseases, seizures, glaucoma, uncontrolled hypertension, diabetes mellitus, stroke
- Current eating disorder (Body mass index > 19)
- Known methylphenidate intolerance
- Treatment with stimulants or ADHD-specific psychotherapy within 6 months prior the screening
- Other psychotherapeutic or psychopharmacological treatment
- Pregnancy or breast-feeding. No reliable contraception (Pearl-Index>1%)

Statistical methods:
<u>Sample size calculation:</u>
The sample size is derived assuming an effect size (Cohen's d) of 0.33 for the two primary 2×2 comparisons (GPT or MPH vs. control conditions). To achieve a power of 80% for a two-sided t-test at significance level 2.5% to adjust for multiplicity, 350 patients are needed. A target of 448 randomizations is planned to compensate for dropouts.

<u>Statistical analysis:</u>
The primary statistical analysis will be conducted according to the intention to treat principle, including all randomized patients as belonging to their randomized arm, regardless of whether they refuse or discontinue therapy, or whether other protocol deviations are known. Treatment comparisons of the primary endpoint will be performed within a linear regression model, with treatment allocation as an independent variable. Baseline measurements of the score will be included as a covariate. Other possibly relevant factors may be considered as well. In a first step, an interaction term will be tested and included in the model if significant at 10%. Then, treatment effects will be presented with 95% and 97.5% confidence intervals (tests are done with two-sided 2.5% level, to account for multiplicity. An adjustment for study center variations will be included. Secondary efficacy endpoints that consist of other scales will be analyzed with the same type of linear model. Rates of adverse events and of serious adverse events will be calculated with 95% confidence intervals. Subgroup analyses will be conducted in an exploratory manner. Before the start of the analysis, a detailed statistical analysis plan (SAP) will be prepared by the

responsible statistician. The SAP will be completed during the 'blind review' of the data, at the latest. The blind review will be performed after the end of the recruitment period and the treatment period. In case of missing values, LOCF-analysis (last observation carried forward) will be performed.

Changes in the Conduct of the study (Amendments):

Amended protocol 1, 29.11.2006: Safety-related exclusion criteria (diabetes mellitus, fasting blood glucose >110 mg/dl, hyperlipidaemia, hypertension according to the guidelines of the German hypertension league, any manifestation of a vascular disease, known intolerance to methylphenidate) and specified information on the exclusion-criterion relating to pregnancy and contraception were added. Minor adaptions were made with regard to physical and laboratory examinations.

Amendment 2, protocol version 3, 19.04.2007: The cerebral MRI scan can be performed from the date of screening. "Missed days of work" is not documented at week 0. Cocaine was added to the drug screening; Further blood samples are taken at selected centres for determination of nicotine and cotinine serum levels at T1-T4.

Amendment 3, protocol version 4, 09.07.2007: A pathological EEG was added to the exclusion criteria. The exclusion criterion of eating disorders was specified (BMI < 19). Instructions for the conduct and preferred treatment (SSRI, with the exception of fluoxetine) in case of the occurrence of a depressive episode during the study were given. Weight loss up to a BMI of <18 was added as a criterion to discontinue the study medication.

Amendment 4, protocol version 5, 17.12.2008: "Laboratory results without a clinically relevant abnormality" were added as an inclusion criterion.

Amendment 5, protocol version 6, 15.8.2009: A follow-up visit (T5), 1.5 years after T4 was added. Accordingly, the timeline of the study was extended to 5 years. A socioeconomic evaluation (SF-36, EQ5D) was added as a secondary outcome. A repetition of the laboratory screening and pregnancy test in case of randomization after the defined time windows was added to the inclusion criteria.

Changes of the planned analysis in the statistical analysis plan:

The original strategy for dealing with missing post-baseline data was changed from last observation carried forward (LOCF) to a multiple imputation method during blinded revision of the statistical analysis plan. This was done to correct for known statistical deficiencies of LOCF while it maintained the original model of a stable response after dropout. Missing post-baseline data were to be replaced using multiple imputation through last mean carried forward (LMCF) in an ANCOVA linear model, using time, treatments, center, and T1 measurement as fixed covariates (see Jama Psychiatry 72(12): 1199-1210 for details).

UNIVERSITÄTS KLINIKUM FREIBURG

ZKS Freiburg

Projekt Nr.: 070170 (BMBF-ADHD-C1)
EudraCT-Nummer: 2006-00222-31

Vergleich einer strukturierten störungsspezifischen Gruppenpsychotherapie plus Placebo oder Methylphenidat versus einer psychiatrischen Beratung plus Placebo oder Methylphenidat bei ADHS im Erwachsenenalter – eine erste randomisierte Multizenter-Studie

Interimanalyse und finale Analyse

Statistischer Analyseplan (SAP)

Datum: 13.04.2010

Finale Version 01

Funktion	Name	Datum	Unterschrift
Autor Biostatistiker	Dr. Erika Graf	13.04.10	E. Graf
Leiter der klinischen Prüfung	PD Dr. med. Alexandra Philipsen	13.4.10	[illegible]

Inhalt

1. Liste der Abkürzungen 4

2. Einleitung 6

3. Studiendesign 6

4. Zielkriterien 7

5. Gegenstand und Durchführung der Interimanalyse 9

6. Analysis Sets 9

7. Statistische Methoden 10

7.1. Deskriptive Datenanalyse 10

7.2. Behandlung von fehlenden Werten 11

7.3. Multizentrische Studie 11

7.4. Multiplizität 11

7.5. Berechnung von abgeleiteten Variablen (derived variables) 11

7.6. Anpassung der Analysestrategie vor Schließen der Datenbank 12

7.7. Software und Kodierung 13

8. Darstellung der Studienpopulation 13

8.1. Patientenrekrutierung 13

8.2. Screening 13

8.3. Verletzung von Ein- und Ausschlusskriterien 14

8.4. Therapie-Compliance 14

8.5. Compliance mit geplanten Untersuchungen 15

8.6. Patientendisposition 15

9. Analyse der Wirksamkeit 16

9.1. Analyse des primären Zielkriteriums 16

9.2. Weitere Analysen zur Wirksamkeit 17

10. Analyse der Sicherheit und Verträglichkeit 17

10.1. Unerwünschte Ereignisse 17

10.2. Vitalparameter 18

10.3. Laborparameter 18

10.4. Drogenscreening, Schwangerschaftstest, EKG 19

11. Weitere Analysen 19

1. Liste der Abkürzungen

ADHS	Aufmerksamkeitsdefizit-/Hyperaktivitätsstörung
ATC	Anatomisch-Therapeutisch-Chemischer-Code
BMBF	Bundesministerium für Bildung und Forschung
CAARS-S:L	Conners' Adult ADHD Rating Scales –Self-Report: Long Version
CAARS-O:L	Conners' Adult ADHD Rating Scales – Observer: Long Version
CRF	Case Record Form (Prüfbogen)
DMC	Data Monitoring Committee
DSM IV	Diagnostisches und Statistisches Manual Psychischer Störungen, American Psychiatric Association
EA	Endanalyse
FAS	Full Analysis Set
G/B	Gruppentherapie versus Beratung
GM/GP/BM/BP	Gruppentherapie und Methylphenidat versus Gruppentherapie und Placebo versus Beratung und Methylphenidat versus Beratung und Placebo
ITT	Intent-to-treat
LKP	Leiter der Klinischen Prüfung
LOCF	Lost observation carried forward
MedDRA	Medical Dictionary for Regulatory Activities
nvZB	Nicht-vertraulicher Zwischenbericht
PPS	Per Protocol Set
SAF	Safety Set
SCL-90-R	Symptom Checkliste 90 Items überarbeitete Version nach Derogatis
SDQ	Strengths and Difficulties Questionnaire
SKID I und II	Strukturiertes Klinisches Interview für DSM-IV (SKID-I und SKID-II) Achse I: Psychische Störungen / Achse II: Persönlichkeits-

	störungen
SOP	Standard Operating Procedures
SUE	Schwerwiegendes unerwünschtes Ereignis
vZB	vertraulicher Zwischenbericht
ZKS	Zentrum Klinische Studien

2. Einleitung

Dieser Statistische Analyseplan (SAP) basiert auf dem Prüfplan der Studie „Vergleich einer strukturierten störungsspezifischen Gruppenpsychotherapie plus Placebo oder Methylphenidat versus einer psychiatrischen Beratung plus Placebo oder Methylphenidat bei ADHS im Erwachsenenalter – eine erste randomisierte Multizenter-Studie“ in folgenden Versionen:

- Version vom 10.08.2006
- Amended protocol 1, Version 29.11.2006
- Amendment 2, Protokollversion 3, 19.04.2007
- Amendment 3, Protokollversion 4, 09.07.2007
- Amendment 4, Protokollversion 5, 17.12.2008
- Amendment 5, Protokollversion 6, 15.08.2009

und dem ‚DMC-Charter für die Klinischen Studien C1 und C2’, Version 1 vom 11.12.2006. Die statistische Analyse wird gemäß den SOPs des Zentrums Klinische Studien (ZKS) durchgeführt.

Inhalt dieses SAPs ist die Beschreibung der Interimanalyse und der finalen Analyse.

3. Studiendesign

Es handelt sich um eine kontrollierte, randomisierte, placebo-kontrollierte, doppel-blinde Multizenterstudie (4armig, 2x2 faktoriell).

Die randomisierte Interventionen sind:

- Gruppenpsychotherapie: Teilnahme an 12 wöchentlichen Sitzungen einer strukturierten störungsspezifischen Gruppentherapie (auf 12 wöchentliche 2-stündige Gruppensitzungen folgen 10 4-wöchentliche Sitzungen; Gesamtdauer: 52 Wochen).
- Kontrollgruppe: 12 wöchentliche, gefolgt von 4-wöchentlichen psychiatrischen Beratungsterminen über insgesamt 52 Wochen (Vorgehen strukturiert nach schriftlichem Leitfaden, Dauer ca. 15-20 Min./Sitzung)
- Begleitend orale Medikation mit Methylphenidat Retard Kapseln oder Placebo (Zieldosis 0,5 -1,3 mg/kg Körpergewicht) in jeder Untersuchungsbedingung (Gruppenpsychotherapie und Kontrollbedingung)

Vergleiche der randomisierten Interventionen werden primär für Gruppentherapie versus Beratung (**G**/**B**) sowie Methylphenidat versus Placebo (**M**/**P**) durchgeführt. Analysen für deren Kombinationen (**GM**/**GP**/**BM**/**BP**) werden bei Bedarf ergänzend durchgeführt.

Die wichtigsten Erhebungszeitpunkte sind T1 (Baseline, Woche 0), T2 (nach 12. Sitzung, Woche 13), T3 (Woche 26), T4 (Abschlussvisite, Woche 52), T5 (Follow-up 2.5 Jahre, Monat 30). Ausgewählte Skalen werden auch an sogenannten Minivisiten

für LOCF (last observation carried forward) erhoben: t2 (Woche 8), t3 (Woche 20), t4 (Woche 40). Näheres siehe Abschnitt 9 des Prüfplans.

4. Zielkriterien

Primäres Zielkriterium, Wirksamkeit (Abschnitte 10.1 und 13.2.1 des Prüfplans)

- **Conners Adult ADHD Rating Scale (CAARS-O:L, blind-observer rated, 66 Fragen, Conners et al. 1999):** ADHS –Symptomatik nach Fremdeinschätzung gemessen mit dem Summenscore der Conners Adult ADHD Rating Scale, Vergleich T1/T2.

Der primäre Endpunkt wird durch einen Interviewer erfasst, der blind für die Zugehörigkeit des Patienten zur Behandlungsgruppe ist.

Sekundäre Zielkriterien, Wirksamkeit (laut Prüfplan Abschnitt 10.1):

- **CAARS-O:L:** nach „blinder" Fremdeinschätzung (siehe primäres Zielkriterium). Vergleich T1/T3, T1/T4.
- **Subskalen der CAARS-O:L:** nach „blinder" Fremdeinschätzung. Vergleich T1/T2, T1/T3, T1/T4.
- **Conners Adult ADHD Rating Scale (CAARS-S:L – Self-report Scale Long Version, Conners et al. 1999):** siehe primäres Zielkriterium, nach Selbsteinschätzung, Summenscore und Subskalen Vergleich T1/T2, T1/T3, T1/T4.
- **ADHS-Checkliste (ADHS-DC**, **Rösler et al. 2004):** Validierter Fragebogen zur Fremdeinschätzung des Ausmaßes der diagnostischen Kriterien für ADHS/ADS im Erwachsenenalter nach DSM-IV. Insgesamt 18 Fragen (9 für Hyperaktivität/Impulsivität, 9 für Unaufmerksamkeit). Vergleich T1/T2, T1/T3, T1/T4.
- **Allgemeine Psychopathologie (SCL-90-R**, **Derogatis 1977):** Symptom-Checkliste, deutsch: Franke 2002. Die SCL-90-R ist ein Selbstbeurteilungsverfahren zur Erfassung körperlicher und psychischer Symptome durch körperliche und psychische Symptome zeitlich bezogen auf den Tag der Beurteilung und die zurückliegende Woche. Es exisiteren drei übergeordnete Indices zur Profilbeschreibung und 9 Syndromskalen zur Erfassung folgender Bereiche: Somatisierung, Zwanghaftigkeit, Unsicherheit im Sozialkontakt, Depressivität, Ängstlichkeit, Aggressivität/ Feindseligkeit, Phobische Angst, Paranoides Denken und Psychotizismus. Das Verfahren ist international und im deutschen Sprachraum gut eingeführt und umfassend hinsichtlich gängiger Gütekriterien untersucht). Vergleich T1/T2, T1/T3, T1/T4.
- **Beck Depressions Inventar (BDI, Beck 1961):** Das Beck-Depressions-Inventar ist ein national und international verbreitetes und in klinischen Zusammenhängen erfolgreich eingesetztes Selbstbeurteilungsinstrument zur Erfassung des Schweregrades einer depressiven Symptomatik (21 Fragen). Vergleich T1/T2, T1/T3, T1/T4.

- **Clinical Global Impression (CGI, Guy 1976):** Allgemeiner klinischer Eindruck. Erfasst wird der allgemeine Schweregrad (0-7) sowie die allgemeine Änderung nach Fremdeinschätzung (0-7). Vergleich T1/T2, T1/T3, T1/T4.
- **Lebensqualität (Quality of Life Enjoyment and Satisfaction Questionnaire, Q-LES-Q, Endicott et al. 1993) :** 91 Fragen zu Lebensqualität und –zufriedenheit. Erfasst werden die Bereiche: körperliche Gesundheit, Emotionalität, Arbeitsplatz, Haushalt/Alltag, Schule/Ausbildung, Freizeit, Soziale Beziehungen, allgemeine Zufriedenheit.). Vergleich T1/T2, T1/T3, T1/T4.
- Fehlzeiten am Arbeits- bzw. Ausbildungsplatz (in Tagen) während der Studienbehandlung in den 4 Behandlungsbedingungen. Erfassung an den Visiten wöchentlich von Woche 0-12, 4-wöchentlich von Woche 13-52.
- Falls zutreffend: Menge des Nikotin-/Koffeinkonsums in den vergangenen 7 Tagen (Zig/d; durchschnittlich ml/d), Ausmaß nicht-stoffgebundener Süchte in den vergangenen 7 Tagen (durchschnittlich Stunden/d). Vergleich T1/T2, T1/T3, T1/T4.
- nicht Gegenstand des SAPs:
  - Bestimmung neurobiologischer (cerebrale MRT) und genetischer Marker für Therapieresponse (siehe Prüfplan, Anhang Kapitel 18;).
  - Weitere explorative Datenanalyse vorgesehen.

Sekundäre Zielkriterien, Wirksamkeit (in Ergänzung zum Prüfplan Abschnitt 10.1):

- Fehltage am Arbeitsplatz/Ausbildungsplatz in den vier Behandlungs-bedingungen im Vergleich (Prüfplan Abschnitt 13.2).
- Vergleiche der sekundären Zielkriterien (Wirksamkeit) T1/T5 zur Berücksichtigung des Follow-ups (Prüfplan Abschnitt 9.3.6 in Protokollversion 6).
- Fragebogen zur Gruppenpsychotherapieevaluation (Hesslinger et al. 2002): Selbsteinschätzung der Wirksamkeit der Gruppenpsychotherapie und deren Module auf die ADHS Symptomatik durch den Patienten. Erhebung an T4. (Prüfplan Abschnitt 9.3.1)
- nicht Gegenstand des SAPs:
  - gesundheitsökonomische Daten (Prüfplan Abschnitt 9.3.6 in Protokollversion 6).

Die Herleitung der oben genannten Summenscores aus den Einzelitems erfolgt in Übereinstimmung mit den Manualen zu den jeweiligen Fragebögen.

Sicherheit und Verträglichkeit

Die Sicherheit und Verträglichkeit werden während der Studienbehandlung auf der Basis der berichteten unerwünschten Ereignisse, der Laborparameter, der Vitalparameter, des Körpergewichts und des EKGs beurteilt (Prüfplan Abschnitte 9.3, 9.4, 10.2).

5. Gegenstand und Durchführung der Interimanalyse

Eine Zwischenanalyse ist im Prüfplan (Abschnitt 13.6) vorgesehen für den Zeitpunkt, nach dem etwa die Hälfte (etwa 220) Patienten randomisiert und nach der wöchentlichen Studienbehandlung 3 Monate (T3) nachbeobachtet wurden. Die genaue Anzahl wird vorliegenden SAP mit Rücksicht auf die Tatsache, dass gruppenweise randomisiert wird, aufgrund des bisherigen Verlaufs der Rekrutierung auf die 231 bis zum 08.05.2009 randomisierten Patienten festgelegt. Gegenstand der Interimanalyse und des zugehörigen Zwischenberichts ist der Verlauf der Patientenaufnahme, die Compliance sowie Sicherheit und Verträglichkeit für das Patientenkollektiv und den Zeitraum wie oben beschrieben. Parameter für den Wirksamkeitsnachweis werden nicht ausgewertet (nur ihr Vorhandensein, siehe Abschnitt 8.5).

Nach Plausibilitätsprüfung der Daten und Auswertung erstellt die Projektstatistikerin einen nicht-vertraulichen und einen vertraulichen Zwischenbericht. Gegenstand des vertraulichen Zwischenberichts (vZB) sind Sicherheitsaspekte (laut DMC-Charter gilt: Werden Sicherheitsaspekte auf Gruppenebene diskutiert und mitgeteilt, so tagt das DMC geschlossen). Der nicht-vertrauliche Zwischenbericht (nvZB) wird dem Leiter der klinischen Prüfung vorgelegt. Dem Unabhängigen Data Monitoring Committee (DMC, siehe Abschnitt 16.1 im Prüfplan) werden der nvZB und der vZB zur Stellungnahme vorgelegt und von der Studienstatistikerin vorgetragen. Der Inhalt des nvZBs, des vZBs und der Endanalyse (EA) wird im vorliegenden statistischen Analyseplan festgelegt.

Gemäß dem DMC-Charter spricht das DMC Empfehlungen an den Leiter der klinischen Prüfung und an den Vorstand des Forschungsverbundes ‚Effects and mechanisms of psychotherapy in the treatment of attention deficit hyperactivity disorder (ADHD) in children and adults' aus. Dabei wird spätestens ab dem Zeitpunkt der Interimanalyse, was die Zusammenarbeit zwischen dem DMC und dem Vorstand des Forschungsverbundes betrifft, die Projektstatistikerin nicht mehr die Rolle des ZKS-Vertreters im Vorstand wahrnehmen.

6. Analysis Sets

Full analysis set (FAS): Das FAS schließt alle Patienten ein, bei denen Angaben zu dem Hauptzielparameter bei Baselineerhebung T1 vorliegen.

Per protocol set (PPS): Das PPS ist eine Untergruppe des FAS und ist definiert als die Gruppe von Patienten, bei denen keine ernsthaften Protokollverletzungen vorliegen. Protokollverletzungen, die zu einem Ausschluss aus der PPS führen, sind:

- Verletzte Auswahlkriterien: Keine Diagnose einer ADHS nach DSM-IV-Kriterien
- Fehlende Messung des primären Endpunktes an T2. Messungen vorgezogener Abschlussvisiten (T4) werden gegebenenfalls berücksichtigt (siehe Abschnitt 7.2).
- Mangelhafte Therapiecompliance (Definitionen siehe Abschnitt 7.5):

  - Methylphenidat- und Placebo-Arm: Mangelhafte Compliance mit der Medikation (Zwischenauswertung, es wird nicht nach Methylphenidat und Placebo unterschieden)
  - Im Methylphenidat-Arm: mangelhafte Compliance mit der Medikation (Endauswertung)
  - Im Placebo-Arm: Einnahme der Medikation nicht begonnen (Endauswertung)
  - Im Gruppentherapie-Arm: Mangelhafte Compliance mit der Gruppentherapie
  - Im Beratungs-Arm: Mangelhafte Compliance mit der psychiatrischen Beratung

  Im Placebo- und im Beratungs-Arm wird eine geringere Compliance als im Methylphenidat- und im Gruppentherapie-Arm verlangt, um im PPS die Schätzung einer oberen Grenze für den bestmöglichen Therapieeffekt zu erzielen.
- Jedoch werden Patienten im Methylphenidat- bzw. im Gruppentherapie-Arm, die die Medikation bzw. die Gruppentherapie abbrechen wegen unerwünschter Ereignisse, für die ein Zusammenhang mit der Studienmedikation bzw. der Gruppentherapie nicht ausgeschlossen werden kann, in jedem Fall im PPS ausgewertet.

Unklare Fälle, die durch diese Kriterien nicht sicher einzuordnen sind, werden von einem Studienbeteiligten, der blind für die Behandlungsbedingung ist, beurteilt. Eine Revision der Kriterienliste für den Ausschluss aus dem PPS nach der Interimanalyse und vor der finalen Analyse ist vorgesehen. Diese Untersuchungen berücksichtigen insbesondere die Begleitmedikationen und nicht-medikamentösen medizinischen / psychotherapeutischen Begleitbehandlungen und werden in der Art eines „blind review“ durchgeführt, d.h. ohne Kenntnis der Behandlungszuteilung.

Safety analysis set 1 und 2 (SAF1, SAF2): Laut Prüfplan gilt: Jeder Patient gleich welcher Gruppe, der mindestens einmal eine Intervention erhalten hat (einmalige Teilnahme an Gruppentherapie, psychiatrischer Beratung, einmalige Medikationseinnahme von Verum oder Placebo), wird in die Verträglichkeitsanalyse miteinbezogen.

Dies wird wie folgt näher spezifiziert: In den SAF1 werden alle Patienten einbezogen, die mindestens einmal an der Gruppentherapie bzw. einer psychiatrischen Beratung teilgenommen haben (G/B). In den SAF2 werden alle Patienten einbezogen, die mindestens einmal Prüfmedikation (Verum oder Placebo, M/P) einnahmen.

7. Statistische Methoden

7.1. Deskriptive Datenanalyse

In Abhängigkeit vom Messniveau der einzelnen Variablen werden geeignete deskriptive Statistiken, z.B. Häufigkeiten (absolut und/oder prozentual), Anzahl der verfügbaren bzw. nicht verfügbaren Werte (non-missing bzw. missing data),

Mittelwert, Standardabweichung, Standardfehler, Median, unteres und oberes Quartil, sowie Minimum und Maximum und Konfidenzintervalle berechnet und dargestellt. Diese Art der Darstellung wird auch für die während der Auswertung sinnvoll zu berechnenden Variablen(kombinationen) verwendet.

Die graphische Aufbereitung der wesentlichen Variablen erfolgt in Abhängigkeit vom Messniveau der Variablen mittels Histogrammen, Box-Whisker-Plots, Mittelwerts-verläufen oder anderer gebräuchlicher Graphiken.

7.2. Behandlung von fehlenden Werten

Für die Summenscores der Zielkriterien der Wirksamkeitsanalyse gilt:

- Treten bei einem Score bei nicht mehr als 10% der Einzelitems fehlende Werte auf, so wird dieser Score in die Berechnungen mit einbezogen (Missings werden durch den Itemmittelwert, d.h. durch den Mittelwert der übrigen Items des Patienten, ersetzt), es sei denn in den zugehörigen Manualen sind andere Vorgehensweisen festgelegt.
- Vorhandene Werte vorgezogener Abschlussvisiten (T4) werden entsprechend der Anzahl Wochen zwischen Randomisation und vorgezogener Erhebung den jeweils zeitlich nächstliegenden prüfplangemäßen Erhebungszeitpunkten zugeordnet.
- Andere fehlende Werte werden über LOCF in die Analyse einbezogen.
- Bei fehlenden Werten des an T2, T3, T4 durch den verblindeten Rater erhobenen CAARS-O:L werden die an den Minivisiten t2, t3, t4 erhobenen Selbstbeurteilungen (CAARS-S:L) für das LOCF mit herangezogen.

Bei den übrigen Zielkriterien werden die vorhandenen Werte analysiert und, wo zutreffend, die Anzahl fehlender Werte berichtet.

7.3. Multizentrische Studie

In den multivariaten Analysen der Wirksamkeits-Endpunkte wird der Faktor ‚Zentrum' als fester Effekt berücksichtigt.

7.4. Multiplizität

Die primäre Auswertung des primären Endpunktes erfolgt einmalig, daher ist keine Adjustierung für multiple Vergleiche erforderlich. Die Sekundäranalysen sind explorativ und werden nicht bezüglich multipler Vergleiche adjustiert.

7.5. Berechnung von abgeleiteten Variablen (derived variables)

Primäre und sekundäre Zielparameter der Wirksamkeit: werden aus den Einzelitems errechnet, wie in den jeweils zugehörigen Manualen sowie unter Zielkriterien (Abschnitt 4) und unter Behandlung fehlender Werte (Abschnitt 7.2) geschildert.

Einteilung der Diagnosen nach SKID-I (Klinische Störungen) und der Diagnosen nach SKID-II (Persönlichkeitsstörungen): Zusammenfassung nach Oberkategorien des DSM-IV

Woche X: Wenn nicht anders festgelegt, werden Datumsangaben wie folgt in Wochen ab Randomisation umgerechnet: Tage seit Randomisation, geteilt durch 7, gerundet auf eine Nachkommastelle

An T2 verordnete Dosierung: Die bei Erhebung des CAARS-O:L-Summenscores an T2 gültige verordnete Tagesdosis Prüfmedikation

Mangelhafte Compliance mit der Medikation: Mangelhafte Compliance liegt vor, wenn eins der nachfolgenden Kriterien zutrifft:

- Einnahme nicht begonnen oder
- Einnahme vor Woche 13 abgebrochen oder
- [Summe der laut CRF bis Woche 13 eingenommenen Tabletten] / [Summe der laut CRF bis Woche 13 verordneten Tabletten] ist <80% oder > 125%. Da die Compliance nur an den tatsächlich erfolgten Visiten errechnet werden kann, wird die letzte Visite vor Woche 13 herangezogen.

Non-compliance mit der Medikation zwischen T2 und T4: Non-compliance liegt vor, wenn eins der nachfolgenden Kriterien zutrifft:

- Einnahme nicht begonnen oder
- Einnahme nach Woche 13 und vor Woche 48 abgebrochen oder
- [Summe der laut CRF nach Woche 13 bis Woche 48 eingenommenen Tabletten] / [Summe der laut CRF nach Woche 13 bis Woche 48 verordneten Tabletten] ist <80% oder > 125%. Da die Compliance nur an den tatsächlich erfolgten Visiten errechnet werden kann, wird die letzte Visite vor Woche 48 herangezogen.

Mangelhafte Compliance mit der Gruppentherapie: Mangelhafte Compliance liegt vor bei Teilnahme an weniger als 8 (von den 12 prüfplangemäßen) Gruppenterminen bis Woche 13.

Non-compliance mit der Gruppentherapie zwischen T2 und T4 (im Interventionsarm): Non-compliance liegt vor bei Teilnahme an weniger als 5 (von den 7 prüfplangemäßen) Gruppenterminen nach Woche 13 bis Woche 52.

Mangelhafte Compliance mit der Beratung (im Kontrollarm): Mangelhafte Compliance liegt vor, wenn bis Woche 13 kein Beratungstermin stattfand.

7.6. Anpassung der Analysestrategie vor Schließen der Datenbank

Zur Durchführung der Interimanalyse wird die Verblindung temporär und nur für die beschriebenen Analysen und Berichte aufgehoben. Zur Endanalyse können vor Öffnung des Randomisierungscodes, Schließen der Datenbank und Durchführung der Analyse die im statistischen Analyseplan festgelegten Details der Auswertung revidiert werden.

Vor Öffnung des Randomisierungscodes zur Endanalyse wird ein „blind review“ durchgeführt, in dem die folgenden Punkte überprüft werden:

- Verteilung der primären und sekundären Zielgrößen
- Transformation erforderlich
- Streuung
- Extremwerte
- fehlende Werte
- Abbruchrate
- Protokollverletzungen
- Definition von FAS und PPS, Ausschluss von Patienten aus diesen Kollektiven.

7.7. Software und Kodierung

- Die Studie wird am ZKS mit SAS ausgewertet.
- Medikationen werden vom ZKS mit ATC kodiert.
- Unerwünschte Ereignisse werden vom ZKS mit MedDRA kodiert. Bei Angabe mehrerer Diagnosen/Symptome wird das unerwünschte Ereignis vor der Kodierung gesplittet.
- Psychische Störungen werden vom Untersucher nach DSM-IV kodiert.
- Befunde der somatischen Anamnese werden vom Untersucher nach Organsystem laut CRF gruppiert. Entsprechend dem CRF und abweichend vom Prüfplan (Abschnitt 11.3) werden Vorerkrankungen nicht mit MedDRA kodiert.

8. Darstellung der Studienpopulation

8.1. Patientenrekrutierung

Folgende Parameter werden im nvZB und in der EA ausgewertet:
- Rekrutierung (Randomisierung) nach randomisiertem Arm (G/B in der EA auch M/P und GM/GP/BM/BP)
- Rekrutierung (Randomisierung) im zeitlichen Verlauf
- Rekrutierung (Randomisierung) nach Studienzentren

8.2. Screening

Folgende Parameter aus dem Screening werden zusammenfassend ausgewertet – im nvZB für alle Patienten, in der EA in FAS+PPS gesamt sowie getrennt nach randomisiertem Arm (G/B, M/P).

- Demographische Angaben
- Mehrfachwahl-Wortschatz-Intelligenztest (MWT-B; IQ laut CRF), Wender-Utah-Rating-Scale (WURS-k; Summenscore laut CRF)
- ADHS-Anamnese
- Diagnosen nach SKID-I+II: Diagnosen nach SKID-I (Klinische Störungen; aktuelle, frühere, beides) zusammengefasst nach Oberkategorien des DSM-IV, Anzahl SKID-I-Diagnosen (aktuelle, frühere beides), aktuelle Diagnosen nach

SKID-II (Persönlichkeitsstörungen) zusammengefasst nach Oberkategorien des DSM-IV, Anzahl aktueller SKID-II-Diagnosen
- Psychiatrische Vorbehandlung, frühere Psychopharmakotherapie
- Aktuelle Medikation nach ATC-Kategorie
- Somatische Anamnese: Befunde je Organsystem zurückliegend und aktuell
- Körperliche Untersuchung (Befunde je Organsystem)
- Vitalzeichen, EKG, EEG

Weitere Auswertungen von Befunden der Screening- und Baselineuntersuchung sind bei der Analyse der Wirksamkeit (Abschnitt 9) und bei der Analyse der Sicherheit und Verträglichkeit (Abschnitt 10) dargestellt.

Folgende Daten werden patientenweise gelistet – im nvZB für alle randomisierten Patienten, in der EA für alle randomisierten Patienten (einschließlich Angabe Zugehörigkeit FAS/PPS/SAF1/SAF2) getrennt nach randomisierter Gruppe (GM/GP/BM/BP):

- Alter, Geschlecht, Staatsangehörigkeit, ethnische Zugehörigkeit
- Diagnosen nach SKID-I+II mit DSM-IV-Kategorie
- Somatische Anamnese
- Psychiatrische / psychotherapeutische Behandlung in der Vorgeschichte
- Aktuelle Medikation mit ATC-Code(s)
- Körperliche Untersuchung

8.3. Verletzung von Ein- und Ausschlusskriterien

Folgende Parameter werden zusammenfassend ausgewertet – im nvZB für alle randomisierten Patienten, in der EA im FAS nach randomisiertem Arm (G/B und M/P).

- Verletzungen der Ein- und Ausschlusskriterien je Kriterium
- Anzahl mindestens ein Einschlusskriterium verletzt oder ein Ausschlusskriterium erfüllt

8.4. Therapie-Compliance

Folgende Parameter werden zusammenfassend ausgewertet – im nvZB für alle randomisierten Patienten, in der EA im FAS+PPS (Definitionen siehe Abschnitt 7.5).

- Einnahme der Medikation nicht begonnen (nvZB: gesamt und nach Studienzentrum, EA: M/P und nach Studienzentrum)
- Medikation vor Woche 13 abgebrochen (nvZB: gesamt und nach Studienzentrum, EA: M/P und nach Studienzentrum)
- Mangelhafte Compliance mit der Medikation (nvZB: gesamt und nach Studienzentrum, EA: M/P und nach Studienzentrum)
- Patienten, die die Medikation begonnen und nicht vor Woche 13 abgebrochen haben: An T2 verordnete Dosierung in mg und mg/kg Körpergewicht (nvZB: gesamt und nach Studienzentrum, EA: M/P und nach Studienzentrum)

- Nur EA: Non-compliance mit der Medikation zwischen T2 und T4 (M/P)
- Im Gruppentherapie-Arm:
  - Mangelhafte Compliance mit der Gruppentherapie, gesamt und nach Studienzentrum
  - Nur EA: Non-compliance mit der Gruppentherapie zwischen T2 und T4
  - Nur EA: Anzahl wahrgenommener, entschuldigt und unentschuldigt versäumter Gruppentermine, Anzahl wahrgenommener Gruppentermine mit Hauptinhalt laut CRF (12 Kategorien, Anzahl je Kategorie), Anzahl, Hauptinhalte und Gründe für die bis zu drei zusätzlich zur Gruppentherapie wahrgenommenen Einzelpsychotherapiesitzungen
- Im Beratungs-Arm:
  - Mangelhafte Compliance mit der Beratung, gesamt und nach Studienzentrum
  - Nur EA: Anzahl wahrgenommener, entschuldigt und unentschuldigt versäumter Beratungstermine

8.5. Compliance mit geplanten Untersuchungen

Folgende Parameter werden zusammenfassend ausgewertet – im nvZB für alle randomisierten Patienten, in der EA im FAS nach randomisiertem Arm G/B und M/P.

- Vorliegen (ja/nein) und Erhebungswoche (Definition siehe Abschnitt 7.5) des CAARS-O:L-Summenscores an den Zeitpunkten T1, T2, T3, in der EA außerdem an T4 und T5
- Teilnahme an Zusatzuntersuchungen (Genetik und zerebrale Bildgebung)

Für weitere Parameter geht die Anzahl vorliegender/fehlender Befunde aus der Analyse der Wirksamkeit (Abschnitt 9) und der Sicherheit und Verträglichkeit (Abschnitt 10) hervor.

8.6. Patientendisposition

Folgende Parameter werden zusammenfassend ausgewertet:

- Anzahl Screening Failures (unter Studienbedingungen gescreente Patienten-Paare, die nicht randomisiert wurden) - nvZB und EA
- Nur EA: Gründe für Drop-out vor Randomisierung bei Screening Failures
- Anzahl Patienten in den Analyse Sets FAS/PPS/SAF1/SAF2 unter allen randomisierten Patienten (im nvZB: vorläufige Zuordnung zum PPS), mit Kombinationen – im nvZB nach randomisiertem Arm G/B, in der EA nach randomisiertem Arm M/P und G/B.
- In der EA im FAS+PPS: Woche und wichtigster Grund für
  - Letzte Einnahme der Medikation (M/P)
  - Letzte Teilnahme an der Gruppenpsychotherapie (Interventionsarm G)
  - Letzte Teilnahme an der Beratung (Kontrollarm B)
  - Letzte studienbedingte Untersuchung in der einjährigen Studienphase (M/P, G/B)

Folgende Daten werden in der EA patientenweise gelistet, getrennt nach randomisiertem Arm G/B und M/P:

- Zugehörigkeit FAS/PPS/SAF1/SAF2, Datum Randomisation, Erhebungswoche des CAARS-O:L-Summenscores an T1, Woche und wichtigster Grund für
  - Letzte Einnahme der Medikation
  - Letzte Teilnahme an der Gruppenpsychotherapie (Interventionsarm G)
  - Letzte Teilnahme an der Beratung (Kontrollarm B)
  - Letzte studienbedingte Untersuchung in der einjährigen Studienphase
- Aus PPS ausgeschlossene Patienten: Gründe für den Ausschluss, demographische Variablen, primäre und sekundäre Zielparameter

9. Analyse der Wirksamkeit

Die in Abschnitt 9 beschriebenen Auswertungen erfolgen ausschließlich in der EA.

Diese Studie wird nach dem "Intention-to-treat" Prinzip ausgewertet, d.h. dass die zu den Behandlungsarmen randomisierten Patienten als zum jeweiligen Arm zugehörig analysiert werden, unabhängig davon, ob sie die Therapie verweigert, abgebrochen haben oder andere Protokollverletzungen bekannt werden. Die primäre ITT-Analyse der Studie wird auf der Basis des Full Analysis Sets (FAS) durchgeführt. Die Auswertung des Per Protocol Sets (PPS) erfolgt als Sensitivitätsanalyse.

Generell kann angenommen werden, dass mögliche Studienabbrüche eher wegen fehlender Wirksamkeit (und damit häufiger unter Placebo bzw. in der Kontrollgruppe) beobachtet werden als wegen unerwünschter Ereignisse (und damit häufiger unter Methylphenidat bzw. unter Gruppenpsychotherapie). Die primäre Auswertungsstrategie mit LOCF im FAS wird voraussichtlich die Kontroll-Arme begünstigen, da für Patienten nach Studienabbruch ein konstanter weiterer Verlauf angenommen wird, und kann daher als konservativ angesehen werden. Die Sensitivitätsanalyse im PPS begünstigt eher die Interventionen, da vorwiegend Patienten mit schlechter Compliance ausgeschlossen werden.

9.1. Analyse des primären Zielkriteriums

Der Vergleich der Behandlungen in Bezug auf die primäre Zielgröße (Veränderung des von T1 nach T2) erfolgt innerhalb eines linearen Regressionsmodells, in dem die Behandlungen als 2 Faktoren mit jeweils 2 Kategorien (Psychotherapie ja/nein bzw. Medikation mit Methylphenidat ja/nein) formuliert werden.

Baselinewerte des CAARS Scores werden als Kovariable im Modell berücksichtigt. Weiterhin wird im Modell für Studienzentren adjustiert. Die Festlegung weiterer relevanter Kovariablen erfolgt im Rahmen eines Statistischen Analyseplans (SAP) vor Beginn der Auswertung.

Im ersten Schritt der Analyse wird überprüft, ob eine Interaktion vorliegt, indem ein geeigneter Interaktionsterm im Modell formuliert und auf Signifikanz geprüft wird. Sollte der Interaktionsterm signifikant sein zum Niveau 10%, so wird er beibehalten,

wenn das nicht der Fall ist, werden die Behandlungseffekte auf der Basis von 2 Haupteffekten ohne Wechselwirkung geschätzt.

Behandlungseffekte werden mit einem zweiseitigen 95%- Konfidenzintervall berichtet. Der Test auf Signifikanz der Behandlungseffekte wird jeweils zum zweiseitigen Niveau 2.5% durchgeführt, um zu berücksichtigen, dass 2 Haupthypothesen getestet werden.

Die weitere Untersuchung der Behandlungseffekte erfolgt hierarchisch, d.h. sie wird nur dann durchgeführt, wenn sich einer der beiden Haupteffekte als signifikant erwiesen hat. Daher ist hierfür keine weitere Adjustierung des Signifikanzniveaus erforderlich.

Die primäre Analyse erfolgt auf der Basis des oben definierten FAS.

9.2. Weitere Analysen zur Wirksamkeit

Weitere Analysen zur Wirksamkeit umfassen:

- eine längerfristige Bewertung des Therapie-Effekte durch Erhebung des primären Endpunktes zu späteren Zeitpunkten (T3, T4);

- eine Bewertung der Therapie-Effekte an Hand der sekundären Wirksamkeitsparameter zu den Zeitpunkten T2, T3, T4.

Hierzu werden jeweils zur oben beschriebenen primären Analyse analoge Berechnungen durchgeführt, d.h. lineare Regressionsmodelle mit den Veränderungen seit T1 als Zielvariablen unter Einbeziehung der entsprechenden Baseline-Messungen, der Studienzentren, einer möglichen Interaktion sowie eventueller weiterer Kovariablen.

10. Analyse der Sicherheit und Verträglichkeit

Analysen zu Sicherheit und Verträglichkeit erfolgen im SAF1 und SAF2 (siehe Abschnitt 6) nach erhaltener Therapie (SAF1: G/B, SAF2: M/P) und werden im vZB und in der EA berichtet.

10.1. Unerwünschte Ereignisse

Im vZB werden nur unerwünschte Ereignisse (UEs) berücksichtigt, deren Anfang vor Woche 26 liegt. In der EA werden alle unerwünschten Ereignisse berücksichtigt. Sollten UEs mit Anfangsdatum vor dem Randomisationsdatum berichtet worden sein, so gehen diese nicht in zusammenfassende Auswertungen ein, sondern werden nur in einem separaten Listing aufgeführt. Über SUSARs (suspected unexpected serious adverse reactions) wird im Rahmen der Annual Safety Reports berichtet.

Raten beziehen sich jeweils auf die Anzahl Patienten mit mindestens einem UE, nicht auf die Anzahl UEs. Folgendes wird getrennt nach Behandlungsgruppe ausgewertet, wobei Raten mit exaktem zweiseitigem 95%-Konfidenzintervall berichtet werden:

- Anzahl Patienten mit mindestens einem UE, mit UE-Rate; Gesamtzahl UEs, minimale, maximale, mittlere Anzahl UEs je Patient (G/B, M/P)
- Die gleichen Parameter für SUEs (G/B, M/P)
- Die gleichen Parameter für (S)UEs, für die ein Zusammenhang mit der Studienmedikation nicht ausgeschlossen werden kann (M/P)
- Die gleichen Parameter für (S)UEs, für die ein Zusammenhang mit der Gruppentherapie / mit dem Clinical Management (Beratung) nicht ausgeschlossen werden kann (G/B)
- Anzahl Patienten mit mindestens einem (S)UE mit (S)UE-Rate je Organsystem (SOC) und Preferred Term (PT) nach MedDRA (G/B, M/P)
- Anzahl Patienten mit mindestens einem (S)UE mit (S)UE-Rate je Organsystem (SOC) und Preferred Term (PT) nach MedDRA für (S)UEs, für die ein Zusammenhang mit der Studienmedikation nicht ausgeschlossen werden kann (M/P)
- Anzahl Patienten mit mindestens einem (S)UE mit (S)UE-Rate je Organsystem (SOC) und Preferred Term (PT) nach MedDRA für (S)UEs, für die ein Zusammenhang mit mit der Gruppentherapie / mit dem Clinical Management (Beratung) nicht ausgeschlossen werden kann (G/B)

Folgende Daten werden patientenweise gelistet – im vZB und der EA im SAF1 (G/B) und im SAF2 (M/P), getrennt nach randomisierter Gruppe:

- Listing der UEs, deren Anfangsdatum nicht vor dem Randomisationsdatum liegt pro Patient, getrennt nach Behandlungsgruppe, mit folgenden Angaben: Beschreibung des UEs – MedDRA preferred term – MedDRA SOC – Datum Randomisation – Datum Beginn des UE – Datum Ende des UE – Intensität – Vorliegen eines SUE – Zusammenhang mit Studieninterventionen – Maßnahmen – Ausgang des UE
- SUE's werden zusätzlich in einer separaten Liste in der gleichen Weise aufgeführt.

Die UEs aller randomisierten Patienten, deren UE-Anfangsdatum vor dem Randomisationsdatum liegt, werden gegebenenfalls zusätzlich in einer separaten Liste in der gleichen Weise aufgeführt.

10.2. Vitalparameter

Vitalparameter werden deskriptiv getrennt nach Zeitpunkten und Behandlungsgruppe (SAF1: G/B, SAF2: M/P) dargestellt, in Woche 12 (vor T2) und Woche 24 (vor T3) sowie in der EA auch zur Abschlussuntersuchung zusätzlich Veränderung gegenüber Baseline.

10.3. Laborparameter

Labordaten werden in der EA im SAF2 (M/P) zusammenfassend ausgewertet. Hierfür werden Shifttabellen errechnet, d.h. es wird nur qualitativ unterschieden, ob die Werte außerhalb der lokalen Normbereiche lagen und ob Werte außerhalb des Normbereiches als klinisch relevant eingestuft wurden. Zusätzlich werden die

Labordaten zum Zeitpunkt der Eingangsuntersuchung qualitativ wie eben beschrieben ausgewertet. Numerische Analysen je Zeitpunkt werden zusätzlich nur für diejenigen Proben vorgenommen, die zentral im Labor des Freiburger Universitätsklinikums befundet wurden.

10.4. Drogenscreening, Schwangerschaftstest, EKG

Drogenscreening, Schwangerschaftstest und EKG werden deskriptiv getrennt nach Behandlungsgruppe (SAF1: G/B, SAF2: M/P) ausgewertet, im vZB zur Eingangsdiagnostik und zur Woche 24, in der EA auch zur Woche 52.

11. Weitere Analysen

- nvZB: Listing der Begleitmedikation mit Angaben laut CRF, ergänzt um den/die ATC-Code(s)
- nvZB: Listing der nicht-medikamentösen medizinischen / psychotherapeutischen Begleitbehandlungen mit Angaben laut CRF
- EA: Zusammende Auswertung der Begleitmedikation nach gruppierten ATC-Levels, im FAS+PPS nach Therapiearm (G/B, M/P)
- EA: Listings wie oben getrennt nach Behandlungsgruppe (SAF1: G/B, SAF2: M/P)

UNIVERSITÄTS KLINIKUM FREIBURG
Studienzentrum

Projekt Nr.: 070170 (BMBF-ADHD-C1)
EudraCT-Nummer: 2006-00222-31

Vergleich einer strukturierten störungsspezifischen Gruppenpsychotherapie plus Placebo oder Methylphenidat versus einer psychiatrischen Beratung plus Placebo oder Methylphenidat bei ADHS im Erwachsenenalter – eine erste randomisierte Multizenter-Studie

Interimanalyse und finale Analyse

Statistischer Analyseplan (SAP)

Datum: 02.04.2012

Finale Version 02

Funktion	Name	Datum	Unterschrift
Autor Biostatistiker	Dr. Erika Graf	03.04.2012	E. Graf
Leiter der klinischen Prüfung	PD Dr. med. Alexandra Philipsen	02.04.12	[illegible]

Inhalt

1. Liste der Abkürzungen ... 4
2. Einleitung ... 6
3. Studiendesign ... 6
4. Zielkriterien ... 7
5. Gegenstand und Durchführung der Interimanalyse ... 9
6. Analysis Sets ... 9
7. Statistische Methoden ... 11
7.1. Deskriptive Datenanalyse ... 11
7.2. Behandlung von fehlenden Werten ... 11
7.3. Multizentrische Studie ... 12
7.4. Multiplizität ... 12
7.5. Berechnung von abgeleiteten Variablen (derived variables) ... 12
7.6. Anpassung der Analysestrategie vor Schließen der Datenbank ... 13
7.7. Software und Kodierung ... 14
8. Darstellung der Studienpopulation ... 14
8.1. Patientenrekrutierung ... 14
8.2. Screening ... 14
8.3. Verletzung von Ein- und Ausschlusskriterien ... 15

8.4. Therapie-Compliance ... 15

8.5. Compliance mit geplanten Untersuchungen ... 16

8.6. Patientendisposition ... 16

9. Analyse der Wirksamkeit ... 17

9.1. Analyse des primären Zielkriteriums ... 17

9.2. Weitere Analysen zur Wirksamkeit ... 18

10. Analyse der Sicherheit und Verträglichkeit ... 20

10.1. Unerwünschte Ereignisse ... 20

10.2. Vitalparameter ... 21

10.3. Laborparameter ... 21

10.4. Drogenscreening, Schwangerschaftstest, EKG ... 22

11. Weitere Analysen ... 22

1. Liste der Abkürzungen

ADHS	Aufmerksamkeitsdefizit-/Hyperaktivitätsstörung
ATC	Anatomisch-Therapeutisch-Chemischer-Code
BMBF	Bundesministerium für Bildung und Forschung
CAARS-S:L	Conners' Adult ADHD Rating Scales –Self-Report: Long Version
CAARS-O:L	Conners' Adult ADHD Rating Scales – Observer: Long Version
CRF	Case Record Form (Prüfbogen)
DMC	Data Monitoring Committee
DSM IV	Diagnostisches und Statistisches Manual Psychischer Störungen, American Psychiatric Association
EA	Endanalyse
FAS	Full Analysis Set
G/B	Gruppentherapie versus Beratung
GM/GP/BM/BP	Gruppentherapie und Methylphenidat versus Gruppentherapie und Placebo versus Beratung und Methylphenidat versus Beratung und Placebo
ITT	Intent-to-treat
LKP	Leiter der Klinischen Prüfung
LOCF	Last observation carried forward
LMCF	Last mean carried forward
MedDRA	Medical Dictionary for Regulatory Activities
nvZB	Nicht-vertraulicher Zwischenbericht
PPS	Per Protocol Set
SAF	Safety Set
SCL-90-R	Symptom Checkliste 90 Items überarbeitete Version nach Derogatis
SDQ	Strengths and Difficulties Questionnaire

SKID I und II	Strukturiertes Klinisches Interview für DSM-IV (SKID-I und SKID-II) Achse I: Psychische Störungen / Achse II: Persönlichkeits-störungen
SOP	Standard Operating Procedures
SUE	Schwerwiegendes unerwünschtes Ereignis
vZB	vertraulicher Zwischenbericht
ZKS	Zentrum Klinische Studien (inzwischen umbenannt in „Studienzentrum des Universitätsklinikums Freiburg; der alte Name wird in Version 02 des Analyseplans beibehalten)

2. Einleitung

Dieser Statistische Analyseplan (SAP) basiert auf dem Prüfplan der Studie „Vergleich einer strukturierten störungsspezifischen Gruppenpsychotherapie plus Placebo oder Methylphenidat versus einer psychiatrischen Beratung plus Placebo oder Methylphenidat bei ADHS im Erwachsenenalter – eine erste randomisierte Multizenter-Studie“ in folgenden Versionen:

- Version vom 10.08.2006
- Amended protocol 1, Version 29.11.2006
- Amendment 2, Protokollversion 3, 19.04.2007
- Amendment 3, Protokollversion 4, 09.07.2007
- Amendment 4, Protokollversion 5, 17.12.2008
- Amendment 5, Protokollversion 6, 15.08.2009

und dem ‚DMC-Charter für die Klinischen Studien C1 und C2’, Version 1 vom 11.12.2006. Die statistische Analyse wird gemäß den SOPs des Zentrums Klinische Studien (ZKS) durchgeführt.

Inhalt dieses SAPs ist die Beschreibung der Interimanalyse und der finalen Analyse.

3. Studiendesign

Es handelt sich um eine kontrollierte, randomisierte, placebo-kontrollierte, doppelblinde Multizenterstudie (4armig, 2x2 faktoriell).

Die randomisierten Interventionen sind:

- Gruppenpsychotherapie: Teilnahme an 12 wöchentlichen Sitzungen einer strukturierten störungsspezifischen Gruppentherapie (auf 12 wöchentliche 2-stündige Gruppensitzungen folgen 10 4-wöchentliche Sitzungen; Gesamtdauer: 52 Wochen).
- Kontrollgruppe: 12 wöchentliche, gefolgt von 4-wöchentlichen psychiatrischen Beratungsterminen über insgesamt 52 Wochen (Vorgehen strukturiert nach schriftlichem Leitfaden, Dauer ca. 15-20 Min./Sitzung)
- Begleitend orale Medikation mit Methylphenidat Retard Kapseln oder Placebo (Zieldosis 0,5 -1,3 mg/kg Körpergewicht) in jeder Untersuchungsbedingung (Gruppenpsychotherapie und Kontrollbedingung)

Vergleiche der randomisierten Interventionen werden primär für Gruppentherapie versus Beratung (**G**/**B**) sowie Methylphenidat versus Placebo (**M**/**P**) durchgeführt. Analysen für deren Kombinationen (**GM**/**GP**/**BM**/**BP**) werden bei Bedarf ergänzend durchgeführt.

Die wichtigsten Erhebungszeitpunkte sind T1 (Baseline, Woche 0), T2 (nach 12. Sitzung, Woche 13), T3 (Woche 26), T4 (Abschlussvisite, Woche 52), T5 (Follow-up 2.5 Jahre, Monat 30). Ausgewählte Skalen werden auch an sogenannten Minivisiten

für LMCF (last mean carried forward) erhoben: t2 (Woche 8), t3 (Woche 20), t4 (Woche 40). Näheres siehe Abschnitt 9 des Prüfplans.

4. Zielkriterien

Primäres Zielkriterium, Wirksamkeit (Abschnitte 10.1 und 13.2.1 des Prüfplans)

- **Conners Adult ADHD Rating Scale (CAARS-O:L, blind-observer rated, 66 Fragen, Conners et al. 1999):** ADHS –Symptomatik nach Fremdeinschätzung gemessen mit dem Summenscore (ADHD-Index) der Conners Adult ADHD Rating Scale, (ADHD Index) Vergleich T1/T2.

Der primäre Endpunkt wird durch einen Interviewer erfasst, der blind für die Zugehörigkeit des Patienten zur Behandlungsgruppe ist.

Sekundäre Zielkriterien, Wirksamkeit (laut Prüfplan Abschnitt 10.1):

- **CAARS-O:L:** nach „blinder" Fremdeinschätzung (siehe primäres Zielkriterium). Vergleich T1/T3, T1/T4.
- **Subskalen der CAARS-O:L:** nach „blinder" Fremdeinschätzung. Vergleich T1/T2, T1/T3, T1/T4.
- **Conners Adult ADHD Rating Scale (CAARS-S:L – Self-report Scale Long Version, Conners et al. 1999):** siehe primäres Zielkriterium, nach Selbsteinschätzung, Summenscore und Subskalen Vergleich T1/T2, T1/T3, T1/T4.
- **ADHS-Checkliste (ADHS-DC**, **Rösler et al. 2004):** Validierter Fragebogen zur Fremdeinschätzung des Ausmaßes der diagnostischen Kriterien für ADHS/ADS im Erwachsenenalter nach DSM-IV. Insgesamt 18 Fragen (9 für Hyperaktivität/Impulsivität, 9 für Unaufmerksamkeit). Vergleich T1/T2, T1/T3, T1/T4.
- **Allgemeine Psychopathologie (SCL-90-R**, **Derogatis 1977):** Symptom-Checkliste, deutsch: Franke 2002. Die SCL-90-R ist ein Selbstbeurteilungsverfahren zur Erfassung körperlicher und psychischer Symptome durch körperliche und psychische Symptome zeitlich bezogen auf den Tag der Beurteilung und die zurückliegende Woche. Es existieren drei übergeordnete Indices zur Profilbeschreibung und 9 Syndromskalen zur Erfassung folgender Bereiche: Somatisierung, Zwanghaftigkeit, Unsicherheit im Sozialkontakt, Depressivität, Ängstlichkeit, Aggressivität/ Feindseligkeit, Phobische Angst, Paranoides Denken und Psychotizismus. Das Verfahren ist international und im deutschen Sprachraum gut eingeführt und umfassend hinsichtlich gängiger Gütekriterien untersucht). Vergleich T1/T2, T1/T3, T1/T4.
- **Beck Depressions Inventar (BDI, Beck 1961):** Das Beck-Depressions-Inventar ist ein national und international verbreitetes und in klinischen Zusammenhängen erfolgreich eingesetztes Selbstbeurteilungsinstrument zur Erfassung des Schweregrades einer depressiven Symptomatik (21 Fragen). Vergleich T1/T2, T1/T3, T1/T4.

- **Clinical Global Impression (CGI, Guy 1976):** Allgemeiner klinischer Eindruck. Erfasst wird der allgemeine Schweregrad (1-7), die allgemeine Änderung (1-7) und der Efficacy-Index (1-4; Efficacy-Index: Ergänzung des Prüfplans) nach Fremdeinschätzung. Schweregrad: Vergleich T1/T2, T1/T3, T1/T4, Änderung und Efficacy-Index: Auswertung an T2, T3, T4 (Korrektur und Ergänzung des Prüfplans).
- **Lebensqualität (Quality of Life Enjoyment and Satisfaction Questionnaire, Q-LES-Q, Endicott et al. 1993) :** 91 Fragen zu Lebensqualität und –zufriedenheit. Erfasst werden die Bereiche: körperliche Gesundheit, Emotionalität, Arbeitsplatz, Haushalt/Alltag, Schule/Ausbildung, Freizeit, Soziale Beziehungen, allgemeine Zufriedenheit.). Vergleich T1/T2, T1/T3, T1/T4.
- **Fehlzeiten am Arbeits- bzw. Ausbildungsplatz (in Tagen)** während der Studienbehandlung in den 4 Behandlungsbedingungen. Erfassung an den Visiten wöchentlich von Woche 0-12, 4-wöchentlich von Woche 13-52.
- Falls zutreffend: **Menge des Nikotin-/Koffeinkonsums** in den vergangenen 7 Tagen (Zig/d; durchschnittlich ml/d), Ausmaß **nicht-stoffgebundener Süchte** in den vergangenen 7 Tagen (durchschnittlich Stunden/d). Vergleich T1/T2, T1/T3, T1/T4. Nikotin/Koffeinkonsum wurden abweichend vom Prüfplan Abschnitt 9.4 nur an T1, T2, T3, T4, T5 erfasst.
- nicht Gegenstand des SAPs:
  - Bestimmung neurobiologischer (cerebrale MRT) und genetischer Marker für Therapieresponse (siehe Prüfplan, Anhang Kapitel 18;).
  - Weitere explorative Datenanalyse vorgesehen.

Sekundäre Zielkriterien, Wirksamkeit (in Ergänzung zum Prüfplan Abschnitt 10.1):

- **Therapieresponder:** 30-prozentige Reduktion des primären Zielkriteriums (dichotom)
- Vergleiche der **sekundären Zielkriterien (Wirksamkeit) T1/T5** zur Berücksichtigung des Follow-ups (Prüfplan Abschnitt 9.3.6 in Protokollversion 6).
- **Fragebogen zur Gruppenpsychotherapieevaluation** (Hesslinger et al. 2002): Selbsteinschätzung der Wirksamkeit der Gruppenpsychotherapie und deren Module auf die ADHS Symptomatik durch den Patienten. Erhebung an T4. (Prüfplan Abschnitt 9.3.1)
- **Clinical Global Impression (CGI, Guy 1976):** Der Efficacy-Index (siehe oben, sekundäre Zielkriterien laut Prüfplan) ist im Prüfplan nicht erwähnt.
- **Nikotin-/Koffeinkonsum** wurden nur an T1, T2, T3, T4, T5 erfasst (Abweichung vom Prüfplan Abschnitt 9.4)
- nicht Gegenstand des SAPs:
  - gesundheitsökonomische Daten (Prüfplan Abschnitt 9.3.6 in Protokollversion 6).

Die Herleitung der oben genannten Summenscores aus den Einzelitems erfolgt in Übereinstimmung mit den Manualen zu den jeweiligen Fragebögen.

Sicherheit und Verträglichkeit

Die Sicherheit und Verträglichkeit werden während der Studienbehandlung auf der Basis der berichteten unerwünschten Ereignisse, der Laborparameter, der Vitalparameter, des Körpergewichts und des EKGs beurteilt (Prüfplan Abschnitte 9.3, 9.4, 10.2).

5. Gegenstand und Durchführung der Interimanalyse

Der nachfolgende Abschnitt bleibt in Version 02 des SAPs, die nach der Interimanalyse erstellt wurde, unverändert.

Eine Zwischenanalyse ist im Prüfplan (Abschnitt 13.6) vorgesehen für den Zeitpunkt, nach dem etwa die Hälfte (etwa 220) Patienten randomisiert und nach der wöchentlichen Studienbehandlung 3 Monate (T3) nachbeobachtet wurden. Die genaue Anzahl wird vorliegenden SAP mit Rücksicht auf die Tatsache, dass gruppenweise randomisiert wird, aufgrund des bisherigen Verlaufs der Rekrutierung auf die 231 bis zum 08.05.2009 randomisierten Patienten festgelegt. Gegenstand der Interimanalyse und des zugehörigen Zwischenberichts ist der Verlauf der Patientenaufnahme, die Compliance sowie Sicherheit und Verträglichkeit für das Patientenkollektiv und den Zeitraum wie oben beschrieben. Parameter für den Wirksamkeitsnachweis werden nicht ausgewertet (nur ihr Vorhandensein, siehe Abschnitt 8.5).

Nach Plausibilitätsprüfung der Daten und Auswertung erstellt die Projektstatistikerin einen nicht-vertraulichen und einen vertraulichen Zwischenbericht. Gegenstand des vertraulichen Zwischenberichts (vZB) sind Sicherheitsaspekte (laut DMC-Charter gilt: Werden Sicherheitsaspekte auf Gruppenebene diskutiert und mitgeteilt, so tagt das DMC geschlossen). Der nicht-vertrauliche Zwischenbericht (nvZB) wird dem Leiter der klinischen Prüfung vorgelegt. Dem Unabhängigen Data Monitoring Committee (DMC, siehe Abschnitt 16.1 im Prüfplan) werden der nvZB und der vZB zur Stellungnahme vorgelegt und von der Studienstatistikerin vorgetragen. Der Inhalt des nvZBs, des vZBs und der Endanalyse (EA) wird im vorliegenden statistischen Analyseplan festgelegt.

Gemäß dem DMC-Charter spricht das DMC Empfehlungen an den Leiter der klinischen Prüfung und an den Vorstand des Forschungsverbundes ‚Effects and mechanisms of psychotherapy in the treatment of attention deficit hyperactivity disorder (ADHD) in children and adults' aus. Dabei wird spätestens ab dem Zeitpunkt der Interimanalyse, was die Zusammenarbeit zwischen dem DMC und dem Vorstand des Forschungsverbundes betrifft, die Projektstatistikerin nicht mehr die Rolle des ZKS-Vertreters im Vorstand wahrnehmen.

6. Analysis Sets

Full analysis set (FAS): Das FAS schließt alle Patienten ein, bei denen Angaben zu dem Hauptzielparameter bei Baselineerhebung T1 vorliegen.

Per protocol set (PPS): Das PPS ist eine Untergruppe des FAS und ist definiert als die Gruppe von Patienten, bei denen keine ernsthaften Protokollverletzungen vorliegen. Protokollverletzungen, die zu einem Ausschluss aus der PPS führen, sind:

- Verletzte Auswahlkriterien: Keine Diagnose einer ADHS nach DSM-IV-Kriterien
- Fehlende Messung des primären Endpunktes an T2. Messungen vorgezogener Abschlussvisiten (T4) werden gegebenenfalls berücksichtigt (siehe Abschnitt 7.2).
- Mangelhafte Therapiecompliance (Definitionen siehe Abschnitt 7.5):
    - Methylphenidat- und Placebo-Arm: Mangelhafte Compliance mit der Medikation (Zwischenauswertung, es wird nicht nach Methylphenidat und Placebo unterschieden)
    - Im Methylphenidat-Arm: mangelhafte Compliance mit der Medikation (Endauswertung)
    - Im Placebo-Arm: Einnahme der Medikation nicht begonnen (Endauswertung)
    - Im Gruppentherapie-Arm: Mangelhafte Compliance mit der Gruppentherapie
    - Im Beratungs-Arm: Mangelhafte Compliance mit der psychiatrischen Beratung

  Im Placebo- und im Beratungs-Arm wird eine geringere Compliance als im Methylphenidat- und im Gruppentherapie-Arm verlangt, um im PPS die Schätzung einer oberen Grenze für den bestmöglichen Therapieeffekt zu erzielen.
- Jedoch werden Patienten im Methylphenidat- bzw. im Gruppentherapie-Arm, die die Medikation bzw. die Gruppentherapie abbrechen wegen unerwünschter Ereignisse, für die ein Zusammenhang mit der Studienmedikation bzw. der Gruppentherapie nicht ausgeschlossen werden kann, in jedem Fall im PPS ausgewertet.

Unklare Fälle, die durch diese Kriterien nicht sicher einzuordnen sind, werden von einem Studienbeteiligten, der blind für die Behandlungsbedingung ist, beurteilt. Eine Revision der Kriterienliste für den Ausschluss aus dem PPS nach der Interimanalyse und vor der finalen Analyse wurde wie vorgesehen (Version 01 des SAP) durchgeführt. Diese Untersuchungen berücksichtigen insbesondere die Begleitmedikationen und nicht-medikamentösen medizinischen / psychotherapeutischen Begleitbehandlungen und wurden in der Art eines „blind review“ durchgeführt, d.h. ohne Kenntnis der Behandlungszuteilung. Im Ergebnis wurde beschlossen, über die vorgenannten Fälle hinaus primär drei Patienten wegen nicht erlaubter Komedikation und weitere drei wegen nicht erlaubter Begleitbehandlung bis T2 auszuschließen (Mitteilung durch die Leiterin der klinischen Prüfung am 29. und 30.03.2012). Explorative Auswertungen zu weiteren, auch im Blind Review genannten Protokollverstößen können folgen.

Safety analysis set 1 und 2 (SAF1, SAF2): Laut Prüfplan gilt: Jeder Patient gleich welcher Gruppe, der mindestens einmal eine Intervention erhalten hat (einmalige

Teilnahme an Gruppentherapie, psychiatrischer Beratung, einmalige Medikationseinnahme von Verum oder Placebo), wird in die Verträglichkeitsanalyse miteinbezogen.

Dies wird wie folgt näher spezifiziert: In den SAF1 werden alle Patienten einbezogen, die mindestens einmal an der Gruppentherapie bzw. einer psychiatrischen Beratung teilgenommen haben (G/B). In den SAF2 werden alle Patienten einbezogen, die mindestens einmal Prüfmedikation (Verum oder Placebo, M/P) einnahmen.

7. Statistische Methoden

7.1. Deskriptive Datenanalyse

In Abhängigkeit vom Messniveau der einzelnen Variablen werden geeignete deskriptive Statistiken, z.B. Häufigkeiten (absolut und/oder prozentual), Anzahl der verfügbaren bzw. nicht verfügbaren Werte (non-missing bzw. missing data), Mittelwert, Standardabweichung, Standardfehler, Median, unteres und oberes Quartil, sowie Minimum und Maximum und Konfidenzintervalle berechnet und dargestellt. Diese Art der Darstellung wird auch für die während der Auswertung sinnvoll zu berechnenden Variablen(kombinationen) verwendet.

Die graphische Aufbereitung der wesentlichen Variablen erfolgt in Abhängigkeit vom Messniveau der Variablen mittels Histogrammen, Box-Whisker-Plots, Mittelwerts-verläufen oder anderer gebräuchlicher Graphiken.

7.2. Behandlung von fehlenden Werten

Für die Summenscores der Zielkriterien der Wirksamkeitsanalyse gilt:

- Treten bei einem Score bei nicht mehr als 10% der Einzelitems fehlende Werte auf, so wird dieser Score in die Berechnungen mit einbezogen (Missings werden durch den Itemmittelwert, d.h. durch den Mittelwert der übrigen Items des Patienten, ersetzt), es sei denn in den zugehörigen Manualen sind andere Vorgehensweisen festgelegt.
- Vorhandene Werte einschließlich vorgezogener Abschlussvisiten (T4) werden entsprechend der Anzahl Wochen zwischen Randomisation und vorgezogener Erhebung den jeweils passendsten prüfplangemäßen Erhebungszeitpunkten zugeordnet.
  Für die Fremdbeurteilungen und die CAARS-Bögen erfolgt die Zuordnung folgendermaßen: Als Erhebung in Woche 8 gelten Messungen aus Woche >6-10.5, Woche 13: >10.5-18, Woche 20: >18-23, Woche 26: >23-33, Woche 40: > 33-46, Woche 52: >46-Ende (ohne T5). Bei mehreren Erhebungen je Zeitfenster gilt in der Primäranalyse nur die jeweils letzte.
- Andere fehlende Werte werden über multiple Imputation mit dem LMCF-Verfahren nach Carpenter, JR, Roger, JH and Kenward MG (2012; Analysis of longitudinal trials with protocol deviation: a framework for relevant, accessible assumptions and inference via multiple imputation. Revision submitted to

Journal of Biopharmaceutical Statistics) in die Analyse einbezogen. Dies ist eine Änderung im Vergleich zu der im Prüfplan vorgesehenen Auswertung (dort: LOCF). Grund: LMCF garantiert im Gegensatz zu LOCF korrekt geschätzte Standardfehler. Die Änderung ist formal, nicht inhaltlich, denn beide Verfahren unterstellen eine im Mittel stabile Response ab dem Ausscheiden. Die Entscheidung wurde vor Entblindung getroffen.
Bei der Implementierung der multiplen Imputation via LMCF wird primär eine unstrukturierte Kovarianzmatrix für wiederholte Messungen innerhalb eines Patienten unterstellt. Sollten sich Konvergenzprobleme ergeben, so werden nach einem prä-spezifizierten Schema sukzessive alternative Kovarianzmatrizen verwendet, bis sich Konvergenz ergibt.

- Bei fehlenden Werten des an T2, T3, T4 durch den verblindeten Rater erhobenen CAARS-O:L werden die an den Minivisiten t2, t3, t4 erhobenen Selbstbeurteilungen (CAARS-S:L) für das LMCF mit herangezogen.

Bei den übrigen Zielkriterien werden die vorhandenen Werte analysiert und, wo zutreffend, die Anzahl fehlender Werte berichtet.

7.3. Multizentrische Studie

In den multivariaten Analysen der Wirksamkeits-Endpunkte wird der Faktor ‚Zentrum' als fester Effekt berücksichtigt.

7.4. Multiplizität

Die primäre Auswertung des primären Endpunktes erfolgt einmalig, daher ist keine Adjustierung für multiple Vergleiche im zeitlichen Verlauf erforderlich. Die Tatsache, dass zwei Haupthypothesen simultan gestestet werden, wird durch Festlegung des Testniveaus für die zwei Hypothesen auf 2.5% berücksichtigt, sodass das multiple Testniveau von 5% eingehalten wird. Die Sekundäranalysen sind explorativ und werden nicht bezüglich multipler Vergleiche adjustiert.

7.5. Berechnung von abgeleiteten Variablen (derived variables)

Primäre und sekundäre Zielparameter der Wirksamkeit: werden aus den Einzelitems errechnet, wie in den jeweils zugehörigen Manualen sowie unter Zielkriterien (Abschnitt 4) und unter Behandlung fehlender Werte (Abschnitt 7.2) geschildert.

Einteilung der Diagnosen nach SKID-I (Klinische Störungen) und der Diagnosen nach SKID-II (Persönlichkeitsstörungen): Zusammenfassung nach Oberkategorien des DSM-IV

Woche X: Wenn nicht anders festgelegt, werden Datumsangaben wie folgt in Wochen ab Randomisation umgerechnet: Tage seit Randomisation, geteilt durch 7, gerundet auf eine Nachkommastelle

An T2 verordnete Dosierung: Die bei Erhebung des CAARS-O:L-Summenscores (ADHD-Index) an T2 gültige verordnete Tagesdosis Prüfmedikation

Mangelhafte Compliance mit der Medikation: Mangelhafte Compliance liegt vor, wenn eins der nachfolgenden Kriterien zutrifft:

- Einnahme nicht begonnen oder
- Einnahme vor Woche 13 abgebrochen oder
- [Summe der laut CRF bis Woche 13 eingenommenen Tabletten] / [Summe der laut CRF bis Woche 13 verordneten Tabletten] ist <80% oder > 125%. Da die Compliance nur an den tatsächlich erfolgten Visiten errechnet werden kann, wird die letzte Visite vor Woche 13 herangezogen.

Non-compliance mit der Medikation zwischen T2 und T4: Non-compliance liegt vor, wenn eins der nachfolgenden Kriterien zutrifft:

- Einnahme nicht begonnen oder
- Einnahme nach Woche 13 und vor Woche 48 abgebrochen oder
- [Summe der laut CRF nach Woche 13 bis Woche 48 eingenommenen Tabletten] / [Summe der laut CRF nach Woche 13 bis Woche 48 verordneten Tabletten] ist <80% oder > 125%. Da die Compliance nur an den tatsächlich erfolgten Visiten errechnet werden kann, wird die letzte Visite vor Woche 48 herangezogen.

Mangelhafte Compliance mit der Gruppentherapie: Mangelhafte Compliance liegt vor bei Teilnahme an weniger als 8 (von den 12 prüfplangemäßen) Gruppenterminen bis Woche 13.

Non-compliance mit der Gruppentherapie zwischen T2 und T4 (im Interventionsarm): Non-compliance liegt vor bei Teilnahme an weniger als 7 (von den 10 prüfplangemäßen) Gruppenterminen nach Woche 13 bis Woche 52.

Mangelhafte Compliance mit der Beratung (im Kontrollarm): Mangelhafte Compliance liegt vor, wenn bis Woche 13 kein Beratungstermin stattfand.

7.6. Anpassung der Analysestrategie vor Schließen der Datenbank

Zur Durchführung der Interimanalyse wird die Verblindung temporär und nur für die beschriebenen Analysen und Berichte aufgehoben. Zur Endanalyse können vor Öffnung des Randomisierungscodes, Schließen der Datenbank und Durchführung der Analyse die im statistischen Analyseplan festgelegten Details der Auswertung revidiert werden.

Vor Öffnung des Randomisierungscodes zur Endanalyse wird ein „blind review“ durchgeführt, in dem die folgenden Punkte überprüft werden:

- Verteilung der primären und sekundären Zielgrößen
- Transformation erforderlich
- Streuung
- Extremwerte
- fehlende Werte
- Abbruchrate
- Protokollverletzungen
- Definition von FAS und PPS, Ausschluss von Patienten aus diesen Kollektiven.

7.7. Software und Kodierung

- Die Studie wird am ZKS mit SAS ausgewertet.
- Medikationen werden vom ZKS mit ATC kodiert.
- Unerwünschte Ereignisse werden vom ZKS mit MedDRA kodiert. Bei Angabe mehrerer Diagnosen/Symptome wird das unerwünschte Ereignis vor der Kodierung gesplittet.
- Psychische Störungen werden vom Untersucher nach DSM-IV kodiert.
- Befunde der somatischen Anamnese werden vom Untersucher nach Organsystem laut CRF gruppiert. Entsprechend dem CRF und abweichend vom Prüfplan (Abschnitt 11.3) werden Vorerkrankungen nicht mit MedDRA kodiert.

8. Darstellung der Studienpopulation

8.1. Patientenrekrutierung

Folgende Parameter werden im nvZB und in der EA ausgewertet:
- Rekrutierung (Randomisierung) nach randomisiertem Arm (G/B in der EA auch M/P und GM/GP/BM/BP)
- EA: Datum der ersten und der letzten Randomisierung
- Rekrutierung (Randomisierung) im zeitlichen Verlauf
- Rekrutierung (Randomisierung) nach Studienzentren

8.2. Screening

Folgende Parameter aus dem Screening werden zusammenfassend ausgewertet – im nvZB für alle Patienten, in der EA in FAS+PPS gesamt sowie getrennt nach randomisiertem Arm (G/B, M/P, GM/GP/BM/BP).

- Demographische Angaben
- Mehrfachwahl-Wortschatz-Intelligenztest (MWT-B; IQ laut CRF), Wender-Utah-Rating-Scale (WURS-k; Summenscore laut CRF)
- ADHS-Anamnese
- Diagnosen nach SKID-I+II: Diagnosen nach SKID-I (Klinische Störungen; aktuelle, frühere, beides) zusammengefasst nach Oberkategorien des DSM-IV, Anzahl SKID-I-Diagnosen (aktuelle, frühere beides), aktuelle Diagnosen nach SKID-II (Persönlichkeitsstörungen) zusammengefasst nach Oberkategorien des DSM-IV, Anzahl aktueller SKID-II-Diagnosen, numerische Werte des SKID-II-Ergebnisblattes
- Psychiatrische Vorbehandlung, frühere Psychopharmakotherapie
- Aktuelle Medikation nach ATC-Kategorie
- Somatische Anamnese: Befunde je Organsystem zurückliegend und aktuell
- Körperliche Untersuchung (Befunde je Organsystem)

- Vitalzeichen, EKG, EEG

Weitere Auswertungen von Befunden der Screening- und Baselineuntersuchung sind bei der Analyse der Wirksamkeit (Abschnitt 9) und bei der Analyse der Sicherheit und Verträglichkeit (Abschnitt 10) dargestellt.

Folgende Daten werden patientenweise gelistet – im nvZB für alle randomisierten Patienten, in der EA für alle randomisierten Patienten einschließlich Angabe Zugehörigkeit FAS/PPS/SAF1/SAF2 und randomisierter Gruppe (GM/GP/BM/BP):

- Alter, Geschlecht, Staatsangehörigkeit, ethnische Zugehörigkeit
- Diagnosen nach SKID-I+II mit DSM-IV-Kategorie
- Somatische Anamnese
- Psychiatrische / psychotherapeutische Behandlung in der Vorgeschichte
- Aktuelle Medikation mit ATC-Code(s)
- Körperliche Untersuchung

8.3. Verletzung von Ein- und Ausschlusskriterien

Folgende Parameter werden zusammenfassend ausgewertet – im nvZB für alle randomisierten Patienten, in der EA im FAS nach randomisiertem Arm (G/B, M/P, GM/GP/BM/BP).

- Verletzungen der Ein- und Ausschlusskriterien je Kriterium
- Anzahl mindestens ein Einschlusskriterium verletzt oder ein Ausschlusskriterium erfüllt

8.4. Therapie-Compliance

Folgende Parameter werden zusammenfassend ausgewertet – im nvZB für alle randomisierten Patienten, in der EA im FAS+PPS (Definitionen siehe Abschnitt 7.5).

- Einnahme der Medikation nicht begonnen (nvZB: gesamt und nach Studienzentrum, EA: M/P und nach Studienzentrum)
- Medikation vor Woche 13 abgebrochen (nvZB: gesamt und nach Studienzentrum, EA: M/P und nach Studienzentrum)
- Mangelhafte Compliance mit der Medikation (nvZB: gesamt und nach Studienzentrum, EA: M/P und nach Studienzentrum)
- Patienten, die die Medikation begonnen und nicht vor Woche 13 abgebrochen haben: An T2 verordnete Dosierung in mg und mg/kg Körpergewicht (nvZB: gesamt und nach Studienzentrum, EA: M/P und nach Studienzentrum)
- Nur EA: Non-compliance mit der Medikation zwischen T2 und T4 (M/P)
- Im Gruppentherapie-Arm:
  - Mangelhafte Compliance mit der Gruppentherapie, gesamt und nach Studienzentrum
  - Nur EA: Non-compliance mit der Gruppentherapie zwischen T2 und T4
  - Nur EA: Anzahl wahrgenommener, entschuldigt und unentschuldigt versäumter Gruppentermine, Anzahl wahrgenommener Gruppentermine

mit Hauptinhalt laut CRF (12 Kategorien, Anzahl je Kategorie), Anzahl, Hauptinhalte und Gründe für die bis zu drei zusätzlich zur Gruppentherapie wahrgenommenen Einzelpsychotherapiesitzungen

- Im Beratungs-Arm:
  - Mangelhafte Compliance mit der Beratung, gesamt und nach Studienzentrum
  - Nur EA: Anzahl wahrgenommener, entschuldigt und unentschuldigt versäumter Beratungstermine

8.5. Compliance mit geplanten Untersuchungen

Folgende Parameter werden zusammenfassend ausgewertet – im nvZB für alle randomisierten Patienten, in der EA im FAS nach randomisiertem Arm G/B, M/P und GM/GP/BM/BP.

- Vorliegen (ja/nein) und Erhebungswoche (Definition siehe Abschnitt 7.5) des CAARS-O:L-Summenscores (ADHD-Index) an den Zeitpunkten T1, T2, T3, in der EA außerdem an T4 und T5
- Teilnahme an Zusatzuntersuchungen (Genetik und zerebrale Bildgebung)

Für weitere Parameter geht die Anzahl vorliegender/fehlender Befunde aus der Analyse der Wirksamkeit (Abschnitt 9) und der Sicherheit und Verträglichkeit (Abschnitt 10) hervor.

8.6. Patientendisposition

Folgende Parameter werden zusammenfassend ausgewertet:

- Anzahl Screening Failures (unter Studienbedingungen gescreente Patienten-Paare, die nicht randomisiert wurden) - nvZB und EA
- Nur EA: Gründe für Drop-out vor Randomisierung bei Screening Failures
- Anzahl Patienten in den Analyse Sets FAS/PPS/SAF1/SAF2 unter allen randomisierten Patienten (im nvZB: vorläufige Zuordnung zum PPS), mit Kombinationen – im nvZB nach randomisiertem Arm G/B, in der EA nach randomisiertem Arm M/P, G/B und GM/GP/BM/BP.
- In der EA im FAS+PPS: Woche und wichtigster Grund für
  - Letzte Einnahme der Medikation (M/P)
  - Letzte Teilnahme an der Gruppenpsychotherapie (Interventionsarm G)
  - Letzte Teilnahme an der Beratung (Kontrollarm B)
  - Letzte studienbedingte Untersuchung in der einjährigen Studienphase (M/P, G/B)

Folgende Daten werden in der EA patientenweise gelistet, einschließlich Angabe der randomisierten Gruppe (GM/GP/BM/BP):

- Zugehörigkeit FAS/PPS/SAF1/SAF2, Datum Randomisation, Erhebungswoche des CAARS-O:L-Summenscores (ADHD-Index) an T1, Woche und wichtigster Grund für

- Letzte Einnahme der Medikation
- Letzte Teilnahme an der Gruppenpsychotherapie (Interventionsarm G)
- Letzte Teilnahme an der Beratung (Kontrollarm B)
- Letzte studienbedingte Untersuchung in der einjährigen Studienphase

- Aus PPS ausgeschlossene Patienten: Gründe für den Ausschluss, demographische Variablen, primäre und sekundäre Zielparameter

9. Analyse der Wirksamkeit

Die in Abschnitt 9 beschriebenen Auswertungen erfolgen ausschließlich in der EA.

Diese Studie wird nach dem "Intention-to-treat" Prinzip ausgewertet, d.h. dass die zu den Behandlungsarmen randomisierten Patienten als zum jeweiligen Arm zugehörig analysiert werden, unabhängig davon, ob sie die Therapie verweigert, abgebrochen haben oder andere Protokollverletzungen bekannt werden. Die primäre ITT-Analyse der Studie wird auf der Basis des Full Analysis Sets (FAS) durchgeführt. Die Auswertung des Per Protocol Sets (PPS) erfolgt als Sensitivitätsanalyse.

Generell kann angenommen werden, dass mögliche Studienabbrüche eher wegen fehlender Wirksamkeit (und damit häufiger unter Placebo bzw. in der Kontrollgruppe) beobachtet werden als wegen unerwünschter Ereignisse (und damit häufiger unter Methylphenidat bzw. unter Gruppenpsychotherapie). Die primäre Auswertungsstrategie mit LOCF im FAS wird voraussichtlich die Kontroll-Arme begünstigen, da für Patienten nach Studienabbruch ein konstanter weiterer Verlauf angenommen wird, und kann daher als konservativ angesehen werden. Die Sensitivitätsanalyse im PPS begünstigt eher die Interventionen, da vorwiegend Patienten mit schlechter Compliance ausgeschlossen werden.

9.1. Primäre Analyse des primären Zielkriteriums

Primäres Zielkriterium ist die Veränderung von T1 nach T2 in der Conners Adult ADHD Rating Scale (ADHD-Index aus CAARS-O:L, blind-observer rated, 66 Fragen, Conners et al. 1999). Die primäre Analyse erfolgt auf der Basis des oben definierten FAS.

Der Vergleich der Behandlungen in Bezug auf die primäre Zielgröße (Veränderung von T1 nach T2) erfolgt innerhalb eines linearen Regressionsmodells, in dem die Behandlungen als 2 Faktoren mit jeweils 2 Kategorien (Psychotherapie ja/nein bzw. Medikation mit Methylphenidat ja/nein) formuliert werden.

Baselinewerte des CAARS Scores werden als Kovariable im Modell berücksichtigt. Die Berücksichtigung der Baselinewerte steht in Einklang mit der EMEA Richtlinie „Points to Consider on Adjustment for Baseline Covariates“ (CPMP/EWP/2863/99) und führt dazu, dass Ergebnisse des Behandlungsvergleichs unabhängig davon sind, ob Veränderungswerte (von T1 nach T2) oder Outcomes nach Beendigung der

intensiven Therapiephase (T2) analysiert werden. Weiterhin wird im Modell für Studienzentren adjustiert.

Im ersten Schritt der Analyse wird überprüft, ob eine Interaktion vorliegt, indem ein geeigneter Interaktionsterm im Modell formuliert und auf Signifikanz geprüft wird. Sollte der Interaktionsterm (G/B*M/P) signifikant sein zum Niveau 10%, so wird er beibehalten, wenn das nicht der Fall ist, werden die Behandlungseffekte auf der Basis von 2 Haupteffekten ohne Wechselwirkung geschätzt.

Behandlungseffekte G/B und M/P (Haupteffekte) werden mit zweiseitigen 95%- und 97.5%-Konfidenzintervallen berichtet (explorativ und konfirmatorisch). Der Haupteffekt G/B wird unter Annahme einer gleichmäßigen 1:1-Verteilung (M/P) geschätzt, ebenso wird der Haupteffekteffekt von M/P unter Annahme einer gleichmäßigen 1:1-Verteilung (G/B) geschätzt. Die erste Haupthypothese $H_{0,1}$: ‚Es besteht kein Unterschied in Bezug auf das primäre Zielkriterium zwischen den Gruppen, die Psychotherapie erhalten, und den Gruppen, die Clinical Management erhalten' wird getestet gegen die erste Hauptalternative $H_{A,1}$: ‚Die Durchführung von Psychotherapie führt zu einem Unterschied in Bezug auf das primäre Zielkriterium'. Die zweite Haupthypothese $H_{0,2}$ lautet: ‚Es besteht kein Unterschied in Bezug auf das primäre Zielkriterium zwischen den Gruppen, die Medikation erhalten, und den Gruppen, die Placebo erhalten' und wird getestet gegen die zweite Hauptalternative $H_{A,2}$: ‚Die Einnahme von Medikation führt zu einem Unterschied in Bezug auf das primäre Zielkriterium'. Der Test auf Signifikanz der Behandlungseffekte G/B und M/P wird jeweils zum zweiseitigen Niveau 2.5% durchgeführt, um zu berücksichtigen, dass 2 Haupthypothesen getestet werden.

Die weitere konfirmatorische Untersuchung der Behandlungseffekte erfolgt hierarchisch, d.h. sie wird nur dann durchgeführt, wenn sich einer der beiden Haupteffekte als signifikant erwiesen hat. Daher ist hierfür keine weitere Adjustierung des Signifikanzniveaus von 5% erforderlich. Konfirmatorische Tests zusätzlicher Hypothesen werden bis zum ersten nicht-signifikanten Ergebnis in folgender Reihenfolge durchgeführt: 1) GM vs. BM, 2) GM vs. GP, 3) BM vs. BP, 4) GM vs. BP.

Die primäre Analyse erfolgt auf der Basis des oben definierten FAS.

9.2. <u>Primäres Zielkriterium, ergänzende Analyse</u>

- Explorative Durchführung der unter 9.1 geschilderten hierarchischen Analysen auch im Falle nicht-signifikanter Ergebnisse
- Durchführung der primären Analyse im PPS
- Die Verteilung des ADHD-Index (CAARS-O:L) wird im FAS deskriptiv ausgewertet

- für alle Erhebungszeitpunkte und für die Veränderungen T1/T2, T1/T3, T1/T4 und T1/T5.
- jeweils ohne LMCF sowie über Least Squares Means mit LMCF (siehe Abschnitt 7.2)
- jeweils getrennt nach randomisierter Gruppe M/P, G/B und GM/GP/BM/BP
- Sollten nach Durchsicht der Ergebnisse ergänzende explorative Auswertungen sinnvoll scheinen, so werden diese im FAS mit linearen Modellen an den vorhandenen Daten durchgeführt.

- Die Untersuchung von Prädiktoren des Therapieerfolgs erfolgt, indem diese Variablen als Kovariablen (Haupteffekt und Interaktionseffekte mit den Behandlungen G/B, M/P) in das oben beschriebene Modell für die primäre Analyse aufgenommen werden, jedoch nicht via multipler Imputation mit LMCF, sondern an den vorhandenen Daten. Mit Prädiktoren sind dabei Faktoren gemeint, anhand derer Subgruppen identifiziert werden sollen, die besonders von der Intervention profitieren. Zu untersuchende Prädiktoren sind:
  - Primär: Komorbiditäten, psychosozialer Status, Alter, Geschlecht
  - Sekundär: Die Liste der untersuchten Prädiktoren kann noch ergänzt werden, sie muss jedoch stets vollständig berichtet werden.
  - Die Kodierung der Variablen im Regressionsmodell kann datengesteuert in Abhängigkeit von der Verteilung eines Faktors, jedoch nicht in Abhängigkeit seines geschätzten Einflusses auf die primäre Zielgröße festgelegt werden. Die Auswahl der relevanten Prädiktoren erfolgt über ein geeignetes statistisches Selektionsverfahren (z.B. forward selection oder backward elimination), dessen Selektionskriterien nach Erstellung des SAP, aber vor Durchführung der Analyse spezifiert werden.

9.3. Sekundäre Zielkriterien

- Ab Baseline (T1) erhobene psychometrische Zielgrößen (Skalen und Unterskalen, siehe Abschnitt 4) werden mit dem linearen Modell analysiert, das auch für die primäre Analyse der primären Zielgröße angewendet wird.
- Das Therapieresponse-Kriterium wird mit einem logistischen Regressionsmodell untersucht, wobei der Behandlungseffekt als geschätztes Odds Ratio beschrieben wird.
- Deskriptive Auswertung: Die Verteilung der ab Baseline (T1) erhobenen psychometrischen Zielgrößen (Skalen und Unterskalen, siehe Abschnitt 4) nach LMCF wird im FAS via Least Squares Means deskriptiv ausgewertet
  - für alle Erhebungszeitpunkte
  - für die Veränderungen von T1 zu T2, T3, T4 und T5
  - jeweils getrennt nach randomisierter Gruppe G/B, M/P, GM/GP/BM/BP
  - die Anzahl fehlender Werte vor LMCF wird dabei angegeben
- Die deskriptive Auswertung folgender Zielgrößen erfolgt ohne LMCF im FAS: CGI-Allgemeine Änderung, CGI-Efficacy-Index, Fragebogen zur

Psychotherapieevaluation, Fehlzeiten am Arbeitsplatz/Ausbildungsplatz, Nikotin-/Koffeinkonsum und nicht-stoffgebundene Süchte

- für alle Erhebungszeitpunkte
- jeweils getrennt nach randomisierter Gruppe G/B, M/P, GM/GP/BM/BP
- Missing-Patterns über die Zeit hinweg getrennt nach randomisierter Gruppe G/B, M/P, GM/GP/BM/BP
- Veränderungen von T1 zu T2, T3, T4 und T5 bezüglich Fehlzeiten am Arbeitsplatz/Ausbildungsplatz, Nikotin-/Koffeinkonsum und nicht-stoffgebundenen Süchten werden erst nach Durchsicht der vorgenannten deskriptiven Auswertungen nach Bedarf deskriptiv ausgewertet.

- Explorative Regressionmodelle für CGI-Allgemeine Änderung und CGI-Efficacy-Index (erhoben nur an T2, T3, T4, T5) werden an die vorhandenen Daten angepasst. Falls es aufgrund differentieller Missingpatterns nötig erscheint, auch durch Berücksichtigung von Kovariablen, die Einfluss auf das Fehlen zeigen, mit Methoden nach Carpenter und Kenward (Missing data in randomised controlled trials – a practical guide, www.missingdata.org.uk).
- Explorative Regressionmodelle für den Fragebogen zur Psychotherapieevaluation, für die Fehlzeiten am Arbeitsplatz/Ausbildungsplatz, Nikotin-/Koffeinkonsum und nicht-stoffgebundene Süchte werden erst nach Durchsicht der deskriptiven Datenanalyse und der Missing-Patterns bei Bedarf festgelegt.

10. Analyse der Sicherheit und Verträglichkeit

Analysen zu Sicherheit und Verträglichkeit erfolgen im SAF1 und SAF2 (siehe Abschnitt 6) nach erhaltener Therapie (SAF1: G/B, SAF2: M/P) und werden im vZB und in der EA berichtet.

10.1. Unerwünschte Ereignisse

Im vZB werden nur unerwünschte Ereignisse (UEs) berücksichtigt, deren Anfang vor Woche 26 liegt. In der EA werden alle unerwünschten Ereignisse berücksichtigt. Sollten UEs mit Anfangsdatum vor dem Randomisationsdatum berichtet worden sein, so gehen diese nicht in zusammenfassende Auswertungen ein, sondern werden nur in einem separaten Listing aufgeführt. Über SUSARs (suspected unexpected serious adverse reactions) wird im Rahmen der Annual Safety Reports berichtet.

Raten beziehen sich jeweils auf die Anzahl Patienten mit mindestens einem UE, nicht auf die Anzahl UEs. Folgendes wird getrennt nach Behandlungsgruppe ausgewertet, wobei Raten mit exaktem zweiseitigem 95%-Konfidenzintervall berichtet werden:

- Anzahl Patienten mit mindestens einem UE, mit UE-Rate; Gesamtzahl UEs, minimale, maximale, mittlere Anzahl UEs je Patient (G/B, M/P)
- Die gleichen Parameter für SUEs (G/B, M/P)

- Die gleichen Parameter für (S)UEs, für die ein Zusammenhang mit der Studienmedikation nicht ausgeschlossen werden kann (M/P)
- Die gleichen Parameter für (S)UEs, für die ein Zusammenhang mit der Gruppentherapie / mit dem Clinical Management (Beratung) nicht ausgeschlossen werden kann (G/B)
- Anzahl Patienten mit mindestens einem (S)UE mit (S)UE-Rate je Organsystem (SOC) und Preferred Term (PT) nach MedDRA (G/B, M/P)
- Anzahl Patienten mit mindestens einem (S)UE mit (S)UE-Rate je Organsystem (SOC) und Preferred Term (PT) nach MedDRA für (S)UEs, für die ein Zusammenhang mit der Studienmedikation nicht ausgeschlossen werden kann (M/P)
- Anzahl Patienten mit mindestens einem (S)UE mit (S)UE-Rate je Organsystem (SOC) und Preferred Term (PT) nach MedDRA für (S)UEs, für die ein Zusammenhang mit mit der Gruppentherapie / mit dem Clinical Management (Beratung) nicht ausgeschlossen werden kann (G/B)

Folgende Daten werden patientenweise gelistet – im vZB und der EA im SAF1 (G/B) und im SAF2 (M/P), getrennt nach randomisierter Gruppe:

- Listing der UEs, deren Anfangsdatum nicht vor dem Randomisationsdatum liegt pro Patient, getrennt nach Behandlungsgruppe, mit folgenden Angaben: Beschreibung des UEs – MedDRA preferred term – MedDRA SOC – Datum Randomisation – Datum Beginn des UE – Datum Ende des UE – Intensität – Vorliegen eines SUE – Zusammenhang mit Studieninterventionen – Maßnahmen – Ausgang des UE
- SUE's werden zusätzlich in einer separaten Liste in der gleichen Weise aufgeführt.

Die UEs aller randomisierten Patienten, deren UE-Anfangsdatum vor dem Randomisationsdatum liegt, werden gegebenenfalls zusätzlich in einer separaten Liste in der gleichen Weise aufgeführt.

10.2. Vitalparameter

Vitalparameter werden deskriptiv getrennt nach Zeitpunkten und Behandlungsgruppe (SAF1: G/B, SAF2: M/P) dargestellt, in Woche 12 (vor T2) und Woche 24 (vor T3) sowie in der EA auch zur Abschlussuntersuchung zusätzlich Veränderung gegenüber Baseline.

10.3. Laborparameter

Labordaten werden in der EA im SAF2 (M/P) zusammenfassend ausgewertet. Hierfür werden Shifttabellen errechnet, d.h. es wird nur qualitativ unterschieden, ob die Werte außerhalb der lokalen Normbereiche lagen und ob Werte außerhalb des Normbereiches als klinisch relevant eingestuft wurden. Zusätzlich werden die Labordaten zum Zeitpunkt der Eingangsuntersuchung qualitativ wie eben beschrieben ausgewertet. Numerische Analysen je Zeitpunkt werden zusätzlich nur

für diejenigen Proben vorgenommen, die zentral im Labor des Freiburger Universitätsklinikums befundet wurden.

10.4. Drogenscreening, Schwangerschaftstest, EKG

Drogenscreening, Schwangerschaftstest und EKG werden deskriptiv getrennt nach Behandlungsgruppe (SAF1: G/B, SAF2: M/P) ausgewertet, im vZB zur Eingangsdiagnostik und zur Woche 24, in der EA auch zur Woche 52.

11. Weitere Analysen

- nvZB: Listing der Begleitmedikation bis T4 mit Angaben laut CRF, ergänzt um den/die ATC-Code(s)
- nvZB: Listing der nicht-medikamentösen medizinischen / psychotherapeutischen Begleitbehandlungen bis T4 mit Angaben laut CRF
- Blind review: Listings wie oben (im nvZB), jedoch zwecks Verblindung ohne Patientennummer
- EA: Zusammenfassende Auswertung der Begleitmedikation bis T4, nach gruppierten ATC-Levels, im FAS+PPS nach Therapiearm (G/B, M/P, GM/GP/BM/BP)
- EA: Zusammenfassende Auswertung der Einschätzung (an T4) der Mitarbeit des Patienten bei Gruppenpsychotherapie einschließlich Auswertung der Erhebungswoche ab Randomisation, im FAS+PPS nach Therapiearm (G/B, M/P, GM/GP/BM/BP)
- EA: Zusammenfassende Auswertung der Frage (an T4), ob der Notfallumschlag geöffnet wurde, im FAS+PPS nach Therapiearm (G/B, M/P, GM/GP/BM/BP)
- EA: Zusammenfassende Auswertung der Begleitmedikation von T4 bis T5, nach gruppierten ATC-Levels, im FAS nach Therapiearm (G/B, M/P, GM/GP/BM/BP)
- EA: Listings wie oben (im nvZB) getrennt nach Behandlungsgruppe (SAF1: G/B, SAF2: M/P)
- EA: Listings wie oben (im nvZB) für den Zeitraum von T4 bis T5 und getrennt nach Behandlungsgruppe (SAF1: G/B, SAF2: M/P)

12. Historie der Änderungen

Änderungen von Version 01 zu Version 02:

Durchweg wurden Ergänzungen zur Klarstellung wo nötig sowie die Unterteilung in die Therapiearme G/B, M/P, GM/GP/BM/BP eingefügt.

6. Die Definition des PPS wurde nach Blind Review ergänzt.

7.2: Das geplante Verfahren zur Berücksichtigung fehlender Werte wurde von LOCF geändert zu LMCF.

9.1: Die im Prüfplan vorgesehene hiearchische Testung der Behandlungseffekte wurde festgelegt. Abschnitt 9.2 wurde neu eingefügt. 9.3 wurde überarbeitet.

In der Zeit zwischen Erstellung von Version 01 zu Version 02 wurde das ZKS umbenannt. Der neue Name lautet „Studienzentrum des Universitätsklinikums Freiburg“. Der Einfachheit halber wurde dies nur hier und im Abkürzungsverzeichnis angemerkt und nicht in das gesamte Dokument (Version 02) eingefügt.

Universitätsklinik der Albert-Ludwigs-Universität Freiburg
Abteilung für Psychiatrie und Psychotherapie mit Poliklinik
Ärztlicher Direktor: Prof. Dr. Mathias Berger

P R Ü F P L A N

Projektnummer: 070170
EudraCT Nummer: 2006-000222-31

Vergleich einer strukturierten störungsspezifischen Gruppenpsychotherapie plus Placebo oder Methylphenidat versus einer psychiatrischen Beratung plus Placebo oder Methylphenidat bei ADHS im Erwachsenenalter – eine erste randomisierte Multizenter-Studie (BMBF-ADHD-C1)

Amendment 4

Protokollversion 5, 17.12.2008

Leiter der Klinischen Prüfung und Projektkoordination:
Dr. med. A. Philipsen
Abteilung für Psychiatrie und Psychotherapie
Hauptstraße 5, D-79104 Freiburg
0761-270-6931/6501
alexandra.philipsen@uniklinik-freiburg.de

Sponsor:
Universitätsklinikum der Medizinischen Fakultät Freiburg
vertreten durch
Herrn Prof. Mathias Berger
Ärztlicher Direktor der Abteilung für Psychiatrie und Psychotherapie
Hauptstrasse 5, D-79104 Freiburg
0761-270-6506/6505
mathias.berger@uniklinik-freiburg.de

Dieser Prüfplan enthält Informationen, die vertraulich sind. Eine Weitergabe an Personen, die nicht an der Durchführung der Klinischen Studie beteiligt sind und jegliche Publikation bedarf der Zustimmung des Leiters der Klinischen Prüfung (LKPs) oder Sponsors. Diese Beschränkungen beziehen sich gleichermaßen auf alle vertraulichen Informationen und Daten, die Ihnen zukünftig mitgeteilt werden.

Inhaltsverzeichnis

1 Übersicht 5
1.1 Liste der im Prüfplan verwendeten Abkürzungen 5
1.2 Synopsis 6
2 Verantwortung und Zuständigkeiten 9
2.1 Leiter der klinischen Prüfung 9
2.2 Biometrie 10
2.3 Prüfärzte 11
2.4 Sponsor 12
2.5 Weitere Veranwortlichkeiten 13
3 Einleitung 14
3.1 Hintergrund 14
3.2 Rationale 14
4 Studienmedikation 15
4.1 Prüfsubstanz Methylphenidat (Medikinet retard® Kapseln à 10mg) 15
4.1.1 Rezeptur 15
4.1.2 Potentielle Nebenwirkungen 16
4.1.3 Nutzen/Risiko Abwägung 16
4.2 Vergleichsmedikation Placebo 17
4.2.1 Rezeptur der Placebo Kapseln 17
4.2.2 Potentielle Nebenwirkungen 17
4.3 Verpackung und Etikettierung 17
4.4 Dosierungsschema 18
4.5 Komedikation 18
4.6 Lagerung, Verteilung und Bereitstellung der Medikation 18
4.7 Notfallmaßnahmen und Entblindung 19
4.8 Drug Accountability 19
5 Ziele der Studie 19
5.1 Primäre Studienziele 19
5.2 Sekundäre Studienziele 19
6 Studienplan 20
6.1 Studiendesign 20
6.2 Teilnehmende Zentren 20
6.3 Anzahl aufzunehmender Patienten 21
7 Studienpopulation 22
7.1 Charakterisierung der Studienpopulation 22
7.2 Einschlusskriterien 22
7.3 Ausschlusskriterien 22
8 Aufnahme in die Studie 23
8.1 Zeitpunkt der Aufnahme 24
8.2 Modus der Aufnahme in die Studie 24
9 Methode 26
9.1 Zeitplan der Studie 26
9.2 Behandlung 26
9.2.1 Studienbehandlung 26
9.2.2 Definition der Begleitmedikation/zusätzliche Behandlungsmethoden 27
9.3 Untersuchungen 27
9.3.1 Eingangsdiagnostik (Screening) 27
9.3.2 Zeitliche Abfolge der Untersuchungen in der Studienbehandlungsphase 28

9.3.3 Nachuntersuchungen 30
9.3.4 Abschlussuntersuchung 30
9.3.5 Videoaufzeichnungen 30
9.4 Flussdiagramm Studienablauf 31
10 Zielkriterien zur Beurteilung von Wirksamkeit und Verträglichkeit 32
10.1 Wirksamkeit 32
10.2 Verträglichkeit 33
10.2.1 Definition und Dokumentation unerwünschter Ereignisse (UE) 33
10.2.2 Definition und Dokumentation schwerwiegender unerwünschter Ereignisse (SUE) 34
10.2.3 Meldepflichten von SUE 34
10.2.4 Klassifizierung unerwünschter Ereignisse 35
11 Dokumentation der Ergebnisse, Datenmanagement 35
11.1 Dokumentation und Korrektur der Daten 35
11.2 Datenmanagement 36
11.3 Kodierung der Daten 36
12 Qualtätssicherungssystem 36
12.1 Qualitätskontrolle (Monitoring) 36
12.2 Quelldatenvergleich (Source Data Verification [SDV]) 37
12.2.1 Daten, die einer 100%igen SDV unterliegen 37
12.2.2 Daten, die einer 20%igen SDV unterliegen 37
13 Biometrische Planung und Auswertung 37
13.1 Studiendesign 37
13.2 Zielkriterien 37
13.3 Fallzahlkalkulation 38
13.4 Definition der in die Analysen eingehenden Kollektive 39
13.5 Zur Auswertung 39
13.5.1 Darstellung der Daten 39
13.5.2 Analyse des primären Endpunktes 40
13.5.3 Weitere Analysen zur Wirksamkeit 40
13.5.4 Analyse der unerwünschten Ereignisse 40
13.5.5 Auswertung der Laborparameter 41
13.5.6 Auswertung der Vitalparameter 41
13.5.7 Erstellung eines Statistischen Analyseplans 41
13.6 Zwischenanalyse und Abbruchkriterien 41
14 Bedingungen für Protokolländerungen 42
14.1 Data Monitoring Committee (DMC) 42
14.2 Protokolländerungen 42
15 Ethische und rechtliche Grundlagen 42
15.1 Aufklärung und Einwilligungserklärung des Patienten 42
15.2 Behandlungsabbruch/ Studienabbruch 43
15.2.1 Vorzeitiger Studienabbruch durch den Patienten 43
15.2.2 Therapieabbruch durch den Prüfarzt beim einzelnen Patienten 43
15.2.3 Abbruchkriterien für einzelne Teile der Studie oder die gesamte Studie: 44
15.2.4 Studienabbruch in einem Prüfzentrum 44
15.2.5 Vorzeitige Beendigung der gesamten Studie 44
15.3 Datenschutz und Schweigepflicht 45
15.4 Einreichung bei Ethikkommissionen 45
15.5 Genehmigung 45
15.6 Allgemeine Anzeigepflicht 46
15.7 Patienten-/Probandenversicherung 46
16 Studiendokumente und Archivierung 46
16.1 Prüfarzt-Ordner 46
16.2 Dokumentation der Studiendaten 46
16.2.1 Patientenerhebungsbogen 46

16.2.2 Dokumentation der Studiendaten in der Krankenakte 47
16.2.3 Patientenidentifikationsliste 47
16.3 Archivierung 47
17 Vereinbarungen 48
17.1 Studienfinanzierung 48
17.2 Probandengelder 47
17.3 Berichte 48
17.4 Publikation 48
18 Studienbegleitende neurobiologische Untersuchungen 49
18.1 Projekt D1: In-Vivo-MR-Bildgebung 49
18.1.1 Hintergrund und wissenschaftliche Fragestellung der MR-Studie 49
18.1.2 Studieneinschluss 50
18.1.3 Ein- und Ausschlusskriterien 50
18.1.4 Untersuchungsprogramm 50
18.1.5 Auswertung der Daten 50
18.1.6 Ethische Erwägungen 50
18.2 Projekt D2: Genetik 51
18.2.1 Hintergrund und wissenschaftliche Fragestellung der Studie 51
18.2.2 Studieneinschluss 51
18.2.3 Ein- und Ausschlusskriterien 51
18.2.4 Arbeitsprogramm 52
19 Literaturangaben 53
19.1 Literatur C1 53
19.2 Literatur D1 55
20 Genehmigung des Prüfplans 56
21 Anhang 57
21.1 Liste der teilnehmenden Prüfzentren 57
21.2 Verpflichtung des Prüfers zur Prüfplaneinhaltung am Zentrum 58
22 Maßgebliche Richtlinien und Gesetze 59

1 Übersicht

1.1 Liste der im Prüfplan verwendeten Abkürzungen

ADHS	Aufmerksamkeitsdefizit-/Hyperaktivitätsstörung
AEL	Adverse Event Liste
ADHS-DC	ADHS- Checkliste
AMDP	Arbeitsgemeinschaft für Methodik und Dokumentation in der Psychiatrie
AMG	Arzneimittelgesetz
APA	American Psychiatric Organization
ATC	Anatomisch-Therapeutisch-Chemischer-Code
BfArM	Bundesinstitut für Arzneimittel und Medizinprodukte
BMBF	Bundesministerium für Bildung und Forschung
CAARS-S:L	Conners' Adult ADHD Rating Scales –Self-Report: Long Version
CAARS-O-SV	Conners' Adult ADHD Rating Scales – Observer: Short-Version
CGI	Clinical Global Impressions
CONSORT	Consolidated Standard of Reporting Trials
CRF	Case Record Form (Prüfbogen)
DMC	Data Monitoring Committee
DSM IV	Diagnostisches und Statistisches Manual Psychischer Störungen, American Psychiatric Association
EK	Ethikkommission
EMEA	The European Agency for the Evaluation of Medical Products
EU	Europäische Union
GCP	Good Clinical Practice
ICD-10	International Classification of Diseases 10th revision
ICH	International Conference of Harmonization
ITT	Intent-to-treat
kgKG	Kilogramm Körpergewicht
LKP	Leiter der klinischen Prüfung
LOCF	Lost observation carried forward
PI	Principal Investigator
pp	Per-protocol
Q-LES-Q	Quality of Life Enjoyment and Satisfaction Questionnaire
SCL-90-R	Symptom Checkliste 90 Items, überarbeitete Version
SDV	Source Data Verification
SKID I und II	Strukturiertes Klinisches Interview für DSM-IV (SKID-I und SKID-II) Achse I: Psychische Störungen / Achse II: Persönlichkeitsstörungen
SUE	Schwerwiegendes unerwünschtes Ereignis
T4	Tetrajodthyronin [Thyroxin]
TSH	thyreoidstimulierendes Hormon [Thyreotropin]
UAW	Unerwünschte Arzneimittelwirkungen
UE	Unerwünschtes Ereignis
uUE	Unerwartetes unerwünschtes Ereignis
WHO	World Health Organization
WURS-k	Wender-Utah-Rating-Scale – deutsche Kurzform
ZKS	Zentrum klinische Studien

1.2 Synopsis

**Titel**	Vergleich einer strukturierten störungsspezifischen Gruppenpsychotherapie plus Placebo oder Methylphenidat versus einer psychiatrischen Beratung plus Placebo oder Methylphenidat bei ADHS im Erwachsenenalter – eine erste randomisierte Multizenter-Studie
**Kurztitel:**	Wirksamkeit von Psychotherapie bei ADHS im Erwachsenenalter
**Studien-Nr.:**	070170<br>EudraCT Number: 2006-000222-31
**Prüfmedikation:**	**Methylphenidat Retard Kapseln (Medikinet retard®) oder Placebo Kapseln à 10mg**
**Phase**:	Phase III/IV

Ziele der Studie:
Überprüfung der Wirksamkeit und Wirkfaktoren einer störungsspezifischen Gruppenpsychotherapie im Vergleich zu einer medikamentösen Behandlung mit Methylphenidat und Placebo bei ADHS im Erwachsenenalter.

Studiendesign:
Zielkriterien:
Primäres Zielkriterium: Wirksamkeit
ADHS –Symptomatik nach Fremdeinschätzung gemessen mit der Conners Adult ADHD Rating Scale (CAARS-O, blind-observer rated, 66 Fragen Version, Summenscore); Vergleich T1/T2.
Sekundäre Zielkriterien: ADHS –Symptomatik nach Fremdeinschätzung gemessen mit der Conners Adult ADHD Rating Scale (CAARS-O, blind-observer rated, 66 Fragen Version, Summenscore); Vergleich T1/T3, T1/T4 sowie der ADHS-Checkliste (18 Fragen) Vergleich T1/T2, T1/T3, T1/T4.
ADHS-Symptomatik nach Selbsteinschätzung gemessen mit der Conners Adult ADHD Rating Scale (CAARS-S, 66 Fragen);
Allgemeine Psychopathologie (SCL-90-R), allgemeiner klinischer Eindruck (Clinical Global Impression (CGI)), Lebensqualität (Q-LES-Q nach Endicott); Depressivität (Beck Depressions Inventar), Fehlzeiten am Arbeits- bzw. Ausbildungsplatz; falls zutreffend: Menge des Nikotinkonsums, Koffeinkonsums, Ausmaß nicht-stoffgebundener Süchte.
Bestimmung neurobiologischer (cerebrale MRT) und genetischer Marker für Therapieresponse.
Weitere explorative Datenanalyse vorgesehen.
Studiendesign:
kontrollierte, randomisierte, placebo-kontrollierte, doppel-blinde Multizenterstudie (4armig, 2x2 faktoriell)
Behandlung:

- Gruppenpsychotherapie: Teilnahme an 12 wöchentlichen Sitzungen einer strukturierten störungsspezifischen Gruppentherapie (auf 12 wöchentliche 2-stündige Gruppensitzungen folgen 10 4-wöchentliche Sitzungen; Gesamtdauer: 52 Wochen).
- Kontrollgruppe: 12 wöchentliche, gefolgt von 4-wöchentlichen psychiatrischen Beratungsterminen über insgesamt 52 Wochen (Vorgehen strukturiert nach schriftlichem Leitfaden, Dauer ca. 15-20 Min./Sitzung)
- Begleitend orale Medikation mit Methylphenidat Retard Kapseln oder Placebo (Zieldosis 0,5 -1,3 mg/kg Körpergewicht) in jeder Untersuchungsbedingung (Gruppenpsychotherapie und Kontrollbedingung)

Beschreibung der Prüfmedikation:
Retardierte Methylphenidat-Kapseln (Medikinet retard®) à 10mg versus Placebo-Kapseln.
Nutzen: Erhöhung von Konzentrations-, Aufmerksamkeits- und Organisationsfähigkeit, Reduktion von Impulsivität; innerer und motorischer Unruhe.
Risiken: Schlafstörungen, Appetitminderung, nach Studienlage nur milde Blutdruck- und Herzfrequenzerhöhung bei nicht vorbestehender arterieller Hypertonie unter Methylphenidat (Wilens TE et al. 2005)
Prüfmedikation/ Placebo – Dosis und Verabreichungsart:
Die psychopharmakologische Behandlung folgt den Empfehlungen der deutschen Leitlinien

(Ebert et al 2003) mit Methylphenidat als Medikation 1. Wahl. Die Aufdosierung der oralen Medikation (Kapseln) erfolgt in den ersten 6 Wochen schrittweise in 10mg Schritten wöchentlich, dem Körpergewicht entsprechend liegt die Zieldosis bei 0.5-1,3 mg/kgKG täglich, die Dosisfindung erfolgt abhängig von Wirkung und UEW. Bei maximaler Dosierung werden in der 6. Woche 60mg Methylphenidat retard täglich erreicht. Eine weitere Dosiserhöhung kann bei unzureichender Wirkung körpergewichtsangepasst bis zur Woche 12 erfolgen. Eine Dosisanpassung ist auch noch während des gesamten Studienverlaufs möglich. Die Zieldosis von maximal 1,3mg/kgKG darf dabei nicht überschritten werden.

Auswahl der Patienten:
Studienpopulation:
- Rekrutierung von 500 Probanden nach Vorscreening in den Spezialambulanzen für ADHS im Erwachsensenalter der beteiligten Prüfzentren (100 Probanden in jedem Prüfzentrum)
  448 Probanden werden randomisiert

Einschlusskriterien:
- Männer und Frauen.
- Probanden sprechen fließend Deutsch
- Alter 18 bis einschl. 60 Jahre.
- Diagnosestellung der ADHS nach DSM-IV Kriterien.
- Mehr als 30 Punkte auf der Kurzversion der Wender-Utah-Rating-Scale (Retz-Junginger P et al 2003).
- Retrospektiv persistierende ADHS Symptomatik seit Kindheit.
- Schriftliches Einverständnis der Probanden entsprechend internationalen Guidelines und lokaler Gesetzgebung.
- Unauffällige körperliche Untersuchung (einschl. Blutdruck/Herzfrequenz) ohne schwer-wiegenden oder unkontrollierten Befund.
- Laborwerte ohne klinisch relevanten Befund (z.B. Blutbild, Nierenretentionswerte, Leberwerte, Schilddrüsenparameter). EKG and EEG ohne pathologisch relevanten Befund).
- Das Screening wurde vollständig durchgeführt, Laborbefunde liegen nicht länger als 6 Wochen und ggf. Schwangerschaftstest nicht länger als 2 Wochen zurück zum Zeitpunkt der Randomisierung.
- Baselinediagnostik innerhalb von maximal 7 Tagen und Therapiebeginn innerhalb von maximal 14 Tagen nach Randomisierung sind möglich.

Ausschlusskriterien:
- IQ < 85 entsprechend einem Punktwert von < 17 im Mehrfachwahl Wortschatz Intelligenztest (MWT-B)
- Schizophrenie, bipolar affektive Störung, Borderline Persönlichkeitsstörung, antisoziale Persönlichkeitsstörung, Suizidalität oder Selbstverletzungen, Autismus, motorische Tics, Tourette Syndrom.
- Substanzabusus/-abhängigkeit in den vergangenen 6 Monaten vor Screening. Episodischer Konsum ist kein Ausschlusskriterium. Positiver Drogentest im Screening.
- Neurologische Erkrankungen, Krampfanfälle, pathologischer EEG-Befund (Seitendifferenzen, Herdbefund, epilepsietypische Potentiale, siehe auch Kap.7.3), Glaukom, Diabetes mellitus, Nüchtern-Blutzuckerwert >110mg/dl, Hyperlipidämie, nicht eingestellte arterielle Hypertonie (nach Leitlinien der Deutschen Hochdruckliga), Angina pectoris, bekannte arterielle Verschlusskrankheit oder eine andere Manifestation einer Gefäßbeteiligung, bekannte tachykarde Arrhythmien.
- Schlaganfall in der Vorgeschichte.
- Bekannte vergrößerte Prostata.
- Aktuelle Essstörung (Bulimia nervosa, Anorexia nervosa, Body Mass Index < 19).
- Aktuelle oder geplante Schwangerschaft, Stillzeit. Positiver Schwangerschaftstest im Screening,
- Teilnahme an einer klinischen Prüfung innerhalb der letzten 3 Monate vor Beginn dieser Studie, bzw. gleichzeitige Teilnahme an einer anderen klinischen Prüfung, vorherige Teilnahme an dieser Studie.
- Medikation mit Stimulanzien oder ADHS-spezifische Psychotherapie innerhalb der letzten 6 Monate vor Beginn der Studie.
- Bekannte Überempflindlichkeit gegenüber Methylphenidat, anderen sympathomimetischen Arzneimitteln oder einem der sonstigen Bestandteile
- Widerwille oder Unfähigkeit, sich an die Vorgaben des Studienprotokolls zu halten.
- Geschäftsunfähiger Patient, der nicht in der Lage ist, Wesen, Bedeutung und Tragweite der Studie zu verstehen.
- Aktuelle oder geplante Schwangerschaft, keine Anwendung der definierten

Kontrazeptionsmethoden (siehe Kapitel 7.3); Stillzeit<br>• Neben dem Prüfpräparat gemäß Behandlungsbedingung besteht eine andere psychopharmakologische Medikation vor Behandlungsbeginn oder während der Studienteilnahme (Definition nicht zulässiger Medikation und des geforderten Zeitpunkts des Absetzens vor Behandlungsbeginn, siehe Kapitel 4.5).<br>• Reguläre ambulante Teilnahme an anderer Psychotherapie während der Studienteilnahme
**Methodik:**<br>Diagnostische Interviews (für Untersuchungsbedingung blinder Interviewer bei T1, T2, T3, T4), Selbsteinschätzung der Symptomatik mit validierten Fragebögen (siehe Zielkriterien).<br>**Erfassung der Wirksamkeit:**<br>Unterschiede zwischen den Behandlungsgruppen im Ausmaß der Veränderung der primären und sekundären Zielkriterien zwischen T1 und T2, T3, T4.<br>**Erfassung der Sicherheit und Verträglichkeit:**<br>UEs, Labor- und Vitalparameter, Drogenscreening, EKG, Körpergewicht, Schwangerschaftstest (siehe Kapitel 10.2)
**Statistische Analyse (Fallzahlkalkulation siehe 12.3)**<br>**Analyse der Wirksamkeit:**<br>Die Studie wird nach dem ITT-Prinzip ausgewertet. Die Schätzung der Behandlungseffekte erfolgt im Rahmen eines linearen Regressionsmodells zur Analyse der Veränderungen des primären Endpunktes von T1 nach T2.<br>**Analyse der Sicherheit:**<br>Raten von (schwerwiegenden) unerwünschten Ereignissen berichtet mit 95%-Konfidenzintervall.. (Vitalparameter und Labor siehe 9.3.1.1 und 9.3.1.2.)
**Zeitplan:**<br>Erster Einschluss: 01.01.2007<br>Letzte Abschlussvisite: 30.06.2009<br>Dauer der Rekrutierungsphase: 18 Monate<br>Abschluss von Auswertung / Berichterstellung: Dezember 2009<br>Studienbericht: Die Studien- und Projektleitung vefasst gemeinsam mit dem ZKS den integrierten medizinisch-biometrischen Abschlussbericht.

2 Verantwortung und Zuständigkeiten

2.1 Leiter der klinischen Prüfung

Diese klinische Prüfung wird in Übereinstimmung mit der ICH-GCP, dem AMG und der Deklaration von Helsinki durchgeführt. Es wird bestätigt, dass der Prüfplan, der Prüfbogen und die Anlagen alle zur Durchführung der Studie notwendigen Informationen und Bestimmungen enthalten, dass die Studie vollinhaltlich gemäß diesem Prüfplan durchgeführt und dokumentiert wird, und dass die gesetzlichen Bestimmungen und beschriebenen Vereinbarungen eingehalten werden. Das zustimmende Votum der Ethikkommission liegt dem Prüfarztordner bei.

Leiter der klinischen Prüfung (gemäß § 40 Abs. 4 AMG)

Dr. med. Alexandra Philipsen
Abteilung für Psychiatrie und Psychotherapie
Universitätsklinik der Albert-Ludwigs-Universität Freiburg
Hauptstraße 5, 79104 Freiburg
Tel: 0761-270-6931/6501
Fax: 0761-270-6526/6619
alexandra.philipsen@uniklinik-freiburg.de

____________________________ ________________________
Unterschrift Ort, Datum

2.2 Biometrie

Diese klinische Prüfung wird in Übereinstimmung mit der ICH-GCP, dem AMG und der Deklaration von Helsinki durchgeführt. Es wird bestätigt, dass der Prüfplan, der Prüfbogen und die Anlagen alle zur Durchführung der Studie notwendigen Informationen und Bestimmungen enthalten, dass die Studie vollinhaltlich gemäß diesem Prüfplan durchgeführt und dokumentiert wird, und dass die gesetzlichen Bestimmungen und beschriebenen Vereinbarungen eingehalten werden. Das zustimmende Votum der Ethikkommission liegt dem Prüfarztordner bei.

Biometrie

Dr. rer. nat. Erika Graf
Universitätsklinikum Freiburg - Zentrum Klinische Studien ZKS
Elsässer Straße 2, D-79110 Freiburg
phone: +49-761-203 5000, fax: +49-761-203-6677
erika.graf@uniklinik-freiburg.de

Unterschrift Ort, Datum

2.3 Prüfärzte

Diese klinische Prüfung wird in Übereinstimmung mit der ICH-GCP, dem AMG und der Deklaration von Helsinki durchgeführt. Es wird bestätigt, dass der Prüfplan, der Prüfbogen und die Anlagen alle zur Durchführung der Studie notwendigen Informationen und Bestimmungen enthalten, dass die Studie vollinhaltlich gemäß diesem Prüfplan durchgeführt und dokumentiert wird, und dass die gesetzlichen Bestimmungen und beschriebenen Vereinbarungen eingehalten werden. Das zustimmende Votum der Ethikkommission liegt dem Prüfarztordner bei.
Die Prüfer an den beteiligten Studienzentren werden im Prüfplan nicht aufgeführt. Eine Liste der Prüfer wird den an der Genehmigung der Prüfung beteiligten Kommissionen und Behörden den rechtlichen Bestimmungen entsprechend vorgelegt werden.

Die von den Prüfern zu leistende Verpflichtung zur Prüfplaneinhaltung findet sich in Kapitel 21.

2.4 Sponsor

Diese klinische Prüfung wird in Übereinstimmung mit der ICH-GCP, dem AMG und der Deklaration von Helsinki durchgeführt. Es wird bestätigt, dass der Prüfplan, der Prüfbogen und die Anlagen alle zur Durchführung der Studie notwendigen Informationen und Bestimmungen enthalten, dass die Studie vollinhaltlich gemäß diesem Prüfplan durchgeführt und dokumentiert wird, und dass die gesetzlichen Bestimmungen und beschriebenen Vereinbarungen eingehalten werden. Das zustimmende Votum der Ethikkommission liegt dem Prüfarztordner bei.

Sponsor

Universitätsklinikum der Medizinischen Fakultät
der Albert-Ludwigs-Universität Freiburg
vertreten durch
Herrn Prof. Mathias Berger
Ärztlicher Direktor der Abteilung für Psychiatrie und Psychotherapie
Hauptstraße 5
79014 Freiburg
Tel. 0761-270-6506/6505, Fax: 0761- 270-6523
mathias.berger@uniklinik-freiburg.de

____________________________ ____________________

Unterschrift Ort, Datum

2.5 Weitere Veranwortlichkeiten

Projektleitung D1 (In-Vivo-MR-Bildgebung)
siehe Prüfplan Kapitel 18.1

PD Dr. L. Tebartz van Elst,
Universitätsklinikum Freiburg,
Abteilung für Psychiatrie und Psychotherapie,
Hauptstr. 5, D-79104 Freiburg;
Tel: +49-761-270-6501; Fax: +49-761-270-6619;
Email: tebartzvanelst@uniklinik-freiburg.de

Projektleitung D2 (Genetik)
siehe Prüfplan Kapitel 18.2

Prof. Dr. med. Klaus-Peter Lesch,
Klinik für Psychiatrie und Psychotherapie der Universität Würzburg,
Füchsleinstraße 15, D-97080 Würzburg,
Tel: +49-931-201-77600, Fax: +49-931-201-77620,
E-mail: kplesch@mail.uni-wuerzburg.de

Monitoring, Datenmanagement, Randomisierungsstelle:

Universitätsklinikum Freiburg - Zentrum Klinische Studien ZKS
Elsässer Straße 2, D-79110 Freiburg

Unabhängiges Data Monitoring Commitee (DMC)

Die Zusammensetzung des DMC wird neben dessen Verantwortlichkeiten und Verfahrensweisen werden in einem DMC-Charter festgelegt.

Klinisches Labor

Laboruntersuchungen erfolgen in den Labors der beteiligten Prüfzentren an den Universitätskliniken in Würzburg, Freiburg, Mannheim, Berlin und Homburg/Saar.

Prüfmedikation

Bundesministerium für Bildung und Forschung (BMBF),
MEDICE Arzneimittel Pütter GmbH & Co. KG (Prüfmedikation)
Kuhloweg 37, 58638 Iserlohn
phone: +49-2371-937-357, fax: +49-2371-937-360,
r.fischer@medice.de

3 Einleitung

3.1 Hintergrund

Die Aufmerksamkeitsdefizit-/Hyperativitätsstörung (ADHS) im Erwachsenenalter war im deutschsprachigen Raum bis Ende der 90er Jahre nahezu unbekannt. Sie wurde bis dahin meist als eine mit zunehmendem Alter „ausheilende“ Störung des Kindes- und Jugendalters aufgefasst. Studien haben jedoch verdeutlicht, dass sich die ADHS nicht regelhaft mit dem 18. Lebensjahr „auswächst“ (Faraone et al. 2006). Die Routinebehandlung für Erwachsene mit ADHS ist in Deutschland bei weitem nicht suffizient.
Gekennzeichnet ist die ADHS durch die Kernsymptome motorische Unruhe, Impulsivität und Unaufmerksamkeit. Bezüglich der Kernsymptomatik zeigt sich im Erwachsenenalter zwar häufig eine Reduktion der motorischen Hyperaktivität, eine innere Unruhe, Impulsivität und Aufmerksamkeitsdefizite bleiben aber oft bestehen. Eine neueste Meta-Analyse ergab eine Persistenzrate von ca. 40-60 % einschließlich derer mit Residualsymptomen. 15 % der Betroffenen erfüllten die für die Diagnosestellung einer ADHS geforderten diagnostischen Kriterien auch noch vollständig im Erwachsenalter (Faraone et al 2006). Somit wird die Prävalenz der ADHS im Erwachsenenalter auf 1-4 % geschätzt (Wilens et al. 2002, Steinhausen et al. 2003, Faraone et al 2006, Kessler et al. 2006). Dabei werden doppelt so viele Männer im Erwachsenenalter als erkrankt diagnostiziert im Vergleich zu Frauen. Im Bereich der Erwachsenenpsychiatrie und -psychotherapie ist die hohe Rate an komorbiden Störungen bei der ADHS von besonderer klinischer Bedeutung (z.B. Sucht, affektive Störungen, Angst- und Persönlichkeitsstörungen (Biederman et al. 2004). Auch die Scheidungs-, Arbeitslosigkeits- und Inhaftierungsraten sind erhöht. So wurde etwa bei 30% männlicher Gefängnisinsassen eine ADHS diagnostiziert (Rösler et al. 2004). ADHS kann daher auch als ein „Risikofaktor“ für andere psychische Störungen und psychosoziale Probleme angesehen werden (Brown 2000).
In Deutschland ist gegenwärtig kein Medikament zur Behandlung der ADHS im Erwachsenenalter zugelassen („off-label-use“). Nach den deutschsprachigen Leitlinien gilt die Behandlung mit Methylphenidat aber als Medikation 1. Wahl bei ADHS im Erwachsenenalter (Expertenkonsensus, unterstützt von der DGPPN: Ebert et al. 2003). Eine 2004 erschienene Metaanalyse aller doppelblinden, placebokontrollierten Untersuchungen zur Wirksamkeit von Methylphenidat bei ADHS im Erwachsenenalter ergab eine sehr gute Wirksamkeit vergleichbar mit der im Kindesalter (Faraone et al. 2004).
Zur Wirksamkeit einer psychotherapeutischen Behandlung der ADHS im Erwachsenenalter gab es bis 2002 keine veröffentlichten kontrollierten Studien. Erste Pilotuntersuchungen zu störungsspezifischen Psychotherapieansätzen mit kleinen Fallzahlen (Stevenson et al. 2002, Hesslinger et al. 2002, Safren et al. 2005) zeigten positive Effekte. Insofern besteht ein hoher Bedarf an kontrollierten Studien zur Wirksamkeit von Psychotherapie bei ADHS im Erwachsenenalter. Aufgrund überlappender Symptome der ADHS im Erwachsenenalter und der Borderline-Persönlichkeitsstörung (BPS) lehnt sich das Freiburger Konzept an das Fertigkeitentraining der BPS nach M. Linehan an und wird in ambulanten, wöchentlichen Gruppensitzungen durchgeführt (Zielsymptome: emotionale Instabilität, Impulsivität, Desorganisiertheit, süchtiges Verhalten, gestörte Selbstachtung (Hesslinger et al. 2004). Gerade das Setting in der Gruppe wurde von bisherigen Teilnehmers als wirksam angegeben. Die Auswertung der Multizenter-Anwendungsbeobachtung zur Wirksamkeit des Gruppenpsychotherapieprogramms ergab eine gute Durchführbarkeit auch in anderen Zentren und signifikante Verbesserungen der ADHS Symptomatik nach Beendigung der Therapie (Philipsen et al. submitted).

3.2 Rationale

Ziel des Netzwerkes ist es, die Wirksamkeit einer störungsspezifischen Gruppenpsychotherapie für Erwachsene mit ADHS in einem größeren Patientenkollektiv unter kontrollierten Bedingungen zu untersuchen und mit der Wirksamkeit psychiatrischer Gespräche, einer leitlinienorientierten medikamentösen Behandlung mit Stimulanzien (Methylphenidat) und Placebo zu vergleichen (4-armiges Studiendesign). Darüberhinaus sollen Praediktoren für das Ansprechen auf die jeweilige Behandlung (z.B. psychopathologische, psychosoziale, neurobiologische) identifiziert werden. Das Projekt ist dabei eng verwoben mit den Projekten BMBF-ADHD-D1 und D2 (Kapitel 18).

4 Studienmedikation

Eine 2004 erschienene Metaanalyse aller doppelblinden, placebokontrollierten Untersuchungen zur Wirksamkeit von Methylphenidat bei ADHS im Erwachsenenalter ergab Effektstärken von 0,9 (bzw. 1,3 bei höherer Dosierung) (Faraone et al. 2004). Die Wirksamkeit ist damit als sehr gut zu beurteilen und vergleichbar mit der im Kindesalter.
Weiterhin liegen kontrollierte Wirksamkeitsnachweise zur medikamentösen Behandlung der ADHS bei Erwachsenen für Pharmaka vor, die nicht aus der Gruppe der Stimulanzien sind: z.B. zum Trizyklikum Desipramin (Wilens et al. 1996), zum Noradrenalin- und Dopaminwiederaufnahmehemmer Bupropion (Wilens et al. 2005) und zur Wirksamkeit von Atomoxetin (Spencer et al. 1998; Michelson et al. 2003, Adler et al. 2005) und zum Nikotinrezeptoragonisten ABT-418 (Wilens et al. 1999b).
Kontrollierte Wirksamkeitsnachweise gibt es weiterhin für d-Amphetamin bzw. Amphetaminmischpräparate (Paterson et al. 1999; Spencer et al. 2001; Taylor and Russo 2000) und auch für Pemolin (Wender et al. 1981; Wilens et al. 1999a). Amphetamin ist als Fertigarznei in Deutschland nicht verfügbar. Pemolin ist wegen seines komplexen hepatischen Metabolismus und der Gefahr hepatotoxischer Nebenwirkungen kein Mittel der ersten Wahl. Vergleichsuntersuchungen zur Wirksamkeit von Stimulanzien und den anderen Präparaten gibt es bisher nicht.
Die Behandlung mit Stimulanzien bei der adulten ADHS ist jedoch die mit Abstand am besten untersuchte Behandlungsform. Dabei ergeben sich überzeugende Hinweise, dass eine Behandlung mit Stimulanzien bei Erwachsenen mit ADHS wirksam sein dürfte. Von allen verfügbaren Stimulanzien ist Methylphenidat am besten untersucht.

4.1 Prüfsubstanz Methylphenidat (Medikinet retard® Kapseln à 10mg) MA-Nr. 54569.00.01

4.1.1 Rezeptur

Zusammensetzung der Prüfmedikation
10mg Methylphenidat HCL; Sucrose, Gelatine, Maisstärke, Methacrylsäure-Ethylacrylat-Copolymer (1:1), Talkum, Triethylcitrat, Titandioxid, Polysorbat 80, Natriumhydroxid, Natriumdodecylsulfat, Simeticon, hochdisperses Siliciumdioxid, Poly(vinylalkohol) und Macrogol 3350, Indigocarmin, Aluminiumsalz, Erythrosin, Methylcellulose, Sorbinsäure, gereinigtes Wasser. KH: 1 Kps. 10 mg/20 mg entspr. 0,013 BE/0,012BE.
Klinische Pharmakologie und Pharmakodynamik
Chemisch gesehen stellt Methylphenidat einen basischen Ester der Phenylessigsäure dar. Das Molekül enthält das Phenylethylaminskelett, das für die amphetaminähnlichen Wirkungen verantwortlich gemacht wird. Tierexperimentell wirkt Methylphenidat indirekt sympathomimetisch durch Freisetzung von Noradrenalin und Hemmung der Wiederaufnahme. Dosisabhängig, d. h. mit steigender Konzentration im Zentralnervensystem, setzt Methylphenidat auch Dopamin frei und hemmt dessen Wiederaufnahme (Wilens und Biederman 1992). Der Wirkmechanismus beim Menschen ist nicht vollständig geklärt; es wird jedoch vermutet, dass der Effekt auf eine kortikale Stimulierung und möglicherweise auch auf eine Aktivierung des retikulären Systems zurückzuführen ist.
Pharmakokinetik
Die pharmakokinetischen Eigenschaften von Methylphenidat sind gründlich untersucht worden (Gualtieri et al. 1984; Winsberg et al. 1982, Elia 1991, Greenhill 1995). Danach werden nach üblichen therapeutischen Dosierungen von unretardiertem Methylphenidat nach 1,5 bis 2,5 Stunden (T_{max}) Serumpegel von 6-15 ng/ml (C_{max}) erreicht. Die Halbwertszeit ($T_{1/2}$) liegt bei 2 – 3,5 Stunden. Das Verteilungsvolumen beträgt 1.1 – 3.1 l/kg. Die pharmakokinetischen Grundparameter von Kindern und Erwachsenen scheinen vergleichbar zu sein (Greenhill 1995). Bei unretardierten galenischen Aufbereitungen ist wegen der kurzen Halbwertszeit meist eine Mehrfachgabe am Tag erforderlich d.h. 2-4 Gaben täglich. Die häufige Einnahme kann zu Complianceproblemen führen.
Bei Medikinet retard® ergibt sich durch die veränderte Galenik eine längere Wirkdauer, so dass eine ein- bis zweimalige Gabe täglich ausreicht.
Eine Bioäquivalenzprüfung (N=12) von Methylphenidat retard (1-mal täglich) vs. Methylphenidat nichtretardiert (2-mal täglich) im cross-over design zeigte eine vergleichbare Bioverfügbarkeit. In beiden Formulierungen lag die Konzentration nach 7 Stunden noch bei 4 ng/ml (C_{max}=5.84 (retard) bzw. 5.30 (nichtretardiert). Dann fiel sie ab und lag nach 12 Stunden bei 2 ± 1 ng/ml (mean ± SD). Nach 24 Stunden lag diese bei allen 12 Patienten bei 0 ng/nl. Beim nichtretardierten Präparat fiel ein geringfügig schnellerer

Konzentrationsanstieg nach Einnahme auf und ein stärkerer Abfall bereits 2 Stunden nach Einnahme der Tablette. (Spiss 2000).
Vor der Einnahme des retardierten Präparates sollen die Probanden essen, da ansonsten die Konzentration im Blut schneller ansteigt und erheblich schneller wieder abfällt.

4.1.2 Potentielle Nebenwirkungen

Methylphenidat ist ein zentralnervöses Stimulans mit zentral erregender Wirkung. Unter der Einnahme werden das medulläre respiratorische Zentrum, das retikuläre Aktivierungssystem (führt teils zu Schlafstörungen) und das laterale hypothalamische Zentrum (führt teils zu Appetitminderung) erregt. Nach oraler Verabreichung wird die Wirksubstanz rasch und nahezu vollständig resorbiert. Von den Nebenwirkungen sind alle Altersstufen betroffen.
Gesehen werden vor allem diastolische und systolische Blutdrucksteigerungen und Pulsbeschleunigungen. Diese Symptome wird man vor allem bei hypertoniegefährdeten Erwachsenen kontrollieren müssen (siehe Herz-Kreislaufsystem im folgenden Abschnitt).

Häufigkeit von Nebenwirkungen

Sehr häufig > 10 %, häufig 1-10%, gelegentlich 0.1-1 %, selten 0.01-0.1%, sehr selten < 0.01 %

- Generell sehr häufig: Schlafstörungen,vermehrte Reizbarkeit, Magen-Darm-Beschwerden, Appetitlosigkeit.
- Zentrales und peripheres Nervensystem: Sehr häufig: Kopfschmerzen, Schwindel, Agitation. Häufig: Schläfrigkeit, Dyskinesien. Selten: Akkomodationsstörungen. Sehr selten: Verstärkung von Tics, Auslösen von Tics. Erhöhte Neigung zu Krampfanfällen, psychotische Symptome (z.B. optische Halluzinationen), Depressivität.
- Herz-Kreislauf-System: Häufig: Veränderung der Herzfrequenz (meist Tachykardie). Veränderung des Blutdrucks (meist Erhöhung), Arrythmien, Palpitationen, Selten: Angina pectoris.
  Nach neuerster Studienlage bei nicht vorbestehender arterieller Hypertonie milde Blutdruckerhöhung (Wilens et al. 2005)
- Magen-Darm-Trakt: Häufig: Übelkeit, Erbrechen, Mundtrockenheit. Sehr selten: Leberfunktionsstörung (Transaminasenerhöhung), Diarrhoe oder Obstipation möglich.
- Haut: Häufig: Überempfindlichkeitsreaktion, Konjunktivitis, allergische Hautreaktion.
- Blut: Sehr selten: Leukopenie, Thrombozytopenie.
- Sonstiges: Häufig: Fieber, Arthralgien. Gelegentlich: Gewichtsabnahme, bei plötzlichen absetzen Reboundphänomen möglich.

Abhängigkeits- und Mißbrauchspotential

Manche Stimulanzien sind gebräuchliche Suchtmittel, die vor allem nasal appliziert oder intravenös injiziert werden. Methylphenidat kann in hohen – übertherapeutischen - Dosen und über längere Zeit eingenommen zu Abhängigkeitsentwicklungen führen. Allerdings sind Mitteilungen, wonach Patienten mit ADHS spezifische Abhängigkeitsstörungen von Methylphenidat entwickeln, selten (Goldman et al. 1998, Fulton und Yates 1988). Die verwendeten maximalen therapeutischen Tagesdosierungen (90 mg/die) liegen weit unterhalb der Gebrauchsmuster Stimulanzienabhängiger (Tagesrationen können, nasal oder iv. appliziert, 1000 - 5000 mg/die, in manchen Fällen bis 10g/die betragen). Bei bestimmungsgemäßen Gebrauch (orale Einnahme) ist kein Abhängigkeitspotential bekannt, es werden in den therapeutischen oralen Dosierungen werden keine Euphorie und nur selten eine Tendenz zum Wirkungsverlust mit der Notwendigkeit der Dosisanpassung gesehen. Toleranzbildung ist eine seltene Ausnahme (Wender 1995). Körperliche Abhängigkeitsphänomene treten praktisch nicht auf.
Studien zum Einsatz von Stimulanzien bei Jugendlichen ergaben ein um 85% reduziertes Risiko später eine Substanzabhängigkeit zu entwickeln (Wilens et al. 2003).

Methylphenidat: Überdosierung und Intoxikation

Die Symptome einer akuten Überdosierung sind hauptsächlich auf die Übererregung des Zentralnervensystems und auf übermäßig starke sympathomimetische Effekte zurückzuführen. Die Überdosierungseffekte von Methylphenidat sind typisch für Stimulanzien und betreffen Erwachsene und Kinder in gleicher Weise. Bei zu hohen Tagesdosierungen treten Agitation, manchmal aber auch Sedierung und Somnolenz auf. Psychopathologisches Leitsymptom der Intoxikation ist die Bewusstseinsstörung.

4.1.3 Nutzen/Risiko Abwägung

Die klinische Prüfung wird GCP-konform durchgeführt. Die Studienteilnahme bietet den Patienten die Möglichkeit einer differenzierten Diagnostik und intensiven Therapie mit engmaschiger und langfristig dokumentierter Verlaufskontrolle, wie dies in diesem Umfang in der klinischen Regelversorgung nicht

implementiert ist. Die Höhe der verabreichten Methylphenidat-Dosis entspricht der in wissenschaftlichen Untersuchungen üblichen Tagesdosis von 0.5 bis 1.3 mg/kgKG (mögliche Risiken der Medikation siehe Kapitel 4.1.2). Die Teilnahme an der Gruppenpsychotherapie geht für die Patienten mit keinem bislang bekannten Risiko einher.
Über Nutzen und mögliche Risiken werden die Studienteilnehmer mündlich und schriftlich ausführlich aufgeklärt.

4.2 Vergleichsmedikation Placebo

4.2.1 Rezeptur der Placebo Kapseln

Sucrose, Gelatine, Maisstärke, Methacrylsäure-Ethylacrylat-Copolymer (1:1), Talkum, Triethylcitrat, Titandioxid, Polysorbat 80, Natriumhydroxid, Natriumdodecylsulfat, Simeticon, hochdisperses Siliciumdioxid, Indigocarmin, Aluminiumsalz, Erythrosin, Methylcellulose, Poly(vinylalkohol), Macrogol 3350, Sorbinsäure, gereinigtes Wasser. **KH:** 1 Kps. 10 mg/20 mg entspr. 0,013 BE/0,012 BE.

4.2.2 Potentielle Nebenwirkungen:

Unverträglichkeitsreaktionen auf Zusatzstoffe sind möglich und werden vor Aufnahme in die Studie erfragt.

4.3 Verpackung und Etikettierung

Verpackung:
Methylphenidat-HCL Retard-Kapseln 10 mg oder Placebokapseln zur oralen Einnahme,
Inhalt : XXX Kapseln
Medikationsnummer XXX
Ch.-B.: PL xxx Verwendbar bis xxx
Studien-Nr.: 2006-000222-31, Prüfplancode 070170
Zur klinischen Prüfung bestimmt!
Dosierung nach Anweisung des Prüfarztes
Patient: XXX-XXXX
Sponsor: Universitätsklinikum der Medizinischen Fakultät Freiburg i.Br., LKP: Dr. med. A. Philipsen, Tel.: 0761-270-6501/6931
Hersteller: MEDICE, 58638 Iserlohn
Lagerung nicht über 30°C
Nicht verbrauchte Kapseln wieder zurückgeben
Für Kinder unzugänglich aufzubewahren!

Etikettierung:
Methylphenidat-HCL Retard-Kapseln 10 mg oder Placebokapseln zur oralen Einnahme,
Inhalt : XXX Kapseln
Medikationsnummer XXX
Ch.-B.: PL xxx Verwendbar bis xxx
Studien-Nr.: 2006-000222-31, Prüfplancode 070170
Zur klinischen Prüfung bestimmt!
Dosierung nach Anweisung des Prüfarztes
Patient: XXX-XXXX
Sponsor: Universitätsklinikum der Medizinischen Fakultät Freiburg i.Br., LKP: Dr. med. A. Philipsen, Tel.: 0761-270-6501/6931
Hersteller: MEDICE, 58638 Iserlohn
Lagerung nicht über 30°C
Nicht verbrauchte Kapseln wieder zurückgeben
Für Kinder unzugänglich aufzubewahren!

Beschriftung der Blister:
Methylphenidat-HCL Retard-Kapseln 10 mg oder Placebokapseln zur oralen Einnahme
Sponsor: Universitätsklinikum der Medizinischen Fakultät Freiburg i.Br.
Hersteller: MEDICE, 58638 Iserlohn
Medikationsnummer: XXX

Ch.-B.: PL xxx Verwendbar bis xxx
Studien-Nr.: 2006-000222-31, Prüfplancode 070170

4.4 Dosierungsschema

Zieldosis Methylphenidat 0.5mg bis 1.3 mg/kg KG.

1. Woche 10 mg Methylphenidat retard-Kapseln: 1-0-0 (Tagesdosis 10 mg)
2. Woche 10 mg Methylphenidat retard-Kapseln: 1-1-0 (Tagesdosis 20 mg)
3. Woche 10 mg Methylphenidat retard-Kapseln: 2-1-0 (Tagesdosis 30 mg)
4. Woche 10 mg Methylphenidat retard-Kapseln: 2-2-0 (Tagesdosis 40 mg)
5. Woche 10 mg Methylphenidat retard-Kapseln: 3-2-0 (Tagesdosis 50 mg)
6. Woche 10 mg Methylphenidat retard-Kapseln: 3-3-0 (Tagesdosis 60 mg)
7. Woche bis 12. Woche: Körpergewichtsangepasste weitere Aufdosierung möglich, falls keine hinreichende Wirkung besteht und die Medikation vertragen wird.

Die Einnahme der Kapseln erfolgt oral morgens nach dem Frühstück. Die Einnahme der 2.Kapsel sollte frühestens 6 Stunden später z.B. nach dem Mittagessen erfolgen.
Die Studiendauer umfasst insgesamt 52 Wochen für den einzelnen Patienten. Eine Dosisanpassung ist auch während der gesamten Studiendauer möglich. Eine maximale Dosierung von 1,3mg/kg KG darf nicht überschritten werden.

4.5 Komedikation

Ab 2 Wochen vor der Baseline Untersuchung (T1) ist die Behandlung mit:
- Antidepressiva (z.B. Serotonin-Wiederaufnahemhemmer, Trizyklika)
- Noradrenalin-Wiederaufnahmehemmer (Atomoxetin)
- Bupropion
- Neuroleptika
- Theophylline
- Amantadin
- Antikoagulanzien des Cumarintyps
- Phenylbutazon
- Antazida
- Alpha-Rezeptorantagonisten wie Clonidin

nicht zulässig.

Sollte es während der Studie zum Auftreten einer medikamentös behandlungsbedürftigen depressiven Episode kommen und die Beibehaltung der Studienmedikation klinisch sinnvoll erscheinen, dann sollten vorzugsweise Serotonin-Wiederaufnahmehemmer (mit Ausnahme von Fluoxetin) zur antidepressiven Behandlung eingesetzt werden und entsprechend im CRF dokumentiert werden.

Folgende Medikamente sollten sofern gegeben und medizinisch vertretbar 4 Wochen vor der Baseline Untersuchung (T1) ausgeschlichen werden:
- Fluoxetin
- MAO-Hemmern

Sofern eine Behandlung mit diesen Medikamenten im Rahmen dieser Studie notwendig wird, darf sie dem Patienten nicht vorenthalten werden. Die Studie wird dann für diesen Patienten abgebrochen und die Notwendigkeit der Medikamentengabe dokumentiert.

4.6 Lagerung, Verteilung und Bereitstellung der Medikation

Der Prüfarzt ist verantwortlich dafür, daß die Studienmedikation an einem ordnungsgemäßen Platz gelagert wird (nicht über 30° C), der unzugänglich für Unbefugte ist, so daß nur der Prüfarzt und berechtigte

Personen Zugang zur Studienmedikation haben. Die Lagerung der Studienmedikation erfolgt nach den Vorschriften der Bundesopiumstelle.
Die Lieferung der Studienmedikation seitens des Sponsors wird vom Prüfarzt schriftlich durch Unterzeichnung der entsprechenden Empfangsbestätigung bescheinigt.

4.7 Notfallmaßnahmen und Entblindung

Der Prüfarzt erhält mit der Prüfsubstanz für jede Medikationsnummer je einen Notfallumschlag. Dieser Notfallumschlag wird im Prüfarztordner aufbewahrt. Nach Randomisierung eines Patienten und Mitteilung der Medikationsnummer vermerkt der Prüfarzt unverzüglich die Patientenidentifikationsnummer (Zentrums- und Patientennummer) außen auf dem Notfallumschlag, mit der zugehörigen Medikationsnummer. Der Notfallumschlag darf nur geöffnet werden, wenn im Notfall eine Identifizierung der Prüfmedikation erforderlich ist. Dies ist dann im CRF mit Angabe des Grundes für die Entblindung und mit dem jeweiligen Datum zu dokumentieren. Tritt dies ein, müssen Studienleitung (LKP) und Monitor umgehend informiert werden. Der Patient erhält keine weitere Prüfmedikation, sollte aber entsprechend der Intention-to-Treat Analyse gemäß Prüfplan weiter beobachtet werden. Die Teilnahme an der Gruppenpsychotherapie/Kontrollbehandlung sollte -das Einverständnis des Patienten - vorausgesetzt fortgeführt werden.
Am Ende der Studie sammeln die verantwortlichen Monitore alle Notfallumschläge wieder ein. Es wird darauf geachtet, dass nicht benutzte Notfallumschläge verschlossen sind. Empfang und Rücknahme wird auf einem Formblatt dokumentiert.

Der Notfallumschlag enthält die Angabe der individuellen, zur Medikationsnummer gehörigen Prüfmedikation (Methylphenidat oder Placebo) einschließlich der Zuordnung zur Gruppentherapie bzw. psychiatrischen Einzelgesprächen. Falls er geöffnet wird, sind Datum, Grund und Unterschrift auf dem Formular einzutragen.

4.8 Drug Accountability

Der Prüfarzt darf die Studienmedikation nur an Patienten ausgeben, die in die Studie aufgenommen worden sind. Die Ausgabe der Studienmedikation an Patienten außerhalb dieser klinischen Studie ist nicht gestattet. Die Ausgabe der Studienmedikation durch den Prüfarzt sowie die Rückgabe unverbrauchter Studienmedikation durch den Patienten sind auf dem „Drug Accountability Form“ genauestens vom Prüfarzt zu dokumentieren. Eine lückenlose Dokumentation über den Verbleib der Studienmedikation ist zwingend vorgeschrieben. Fehlbestände sind zu begründen. Jegliche nicht verbrauchte Studienmedikation ist am Ende der Studie an den Sponsor der Prüfmedikation d.h. die Firma Medice zurückzugeben.

5 Ziele der Studie

5.1 Primäre Studienziele

Die Studie dient dazu, folgende Hypothesen bei ADHS im Erwachsenenalter zu überprüfen:

1. Eine störungsspezifische kognitiv-behaviorale psychotherapeutische Behandlung ist einer üblichen Behandlung mit psychiatrischen Gesprächen („Clinical Management“) überlegen (Reduktion der ADHS-Symptomatik gemessen mit standardisiertem Interview, CAARS-O).
2. Die Kombinationsbehandlung aus Methylphenidat und störungsspezifischer psychotherapeutischer Behandlung ist einer alleinigen medikamentösen Behandlung mit Methylphenidat und störungsspezifischen kognitiv-behavioralen psychotherapeutischen Behandlung bei ADHS im Erwachsenenalter überlegen (Reduktion der ADHS-Symptomatik gemessen mit standardisiertem Interview, CAARS-O).

5.2 Sekundäre Studienziele

Wirksamkeit von Methylphenidat und störungsspezifischer psychotherapeutischer Behandlung sowie der Kontrollbedingung mit „Clinical Management“ auf die allgemeine Psychopathologie, allgemeines Funktionsniveau, Depressivität, Lebensqualität, und Fehlzeiten am Arbeitsplatz. Die detaillierte psychometrische Diagnostik der Patienten (z.B. SKID I und II) dient dazu, mögliche Praediktoren für ein

Ansprechen auf die jeweilige Behandung zu untersuchen. Anhand der assozierten Studien zur cerebralen Bildgebung (MR-Spectroskopie und Morphometrie) und genetischen Untersuchungen sollen ebenfalls Praediktoren für ein Ansprechen auf die jeweilige Behandlungsbedingung untersucht werden. Mit der cerebralen Bildgebung werden zudem neurobiologische Korrelate für den Behandlungsverlauf untersucht.

6 Studienplan

6.1 Studiendesign

Studiendesign:
randomisierte, placebo-kontrollierte, doppel-blinde Multizenterstudie.
Die Prüfmedikation (Methylphenidat/Placebo) ist verblindet, der Prüfarzt kennt nur die psychotherapeutische bzw. psychiatrische Behandlungsbedingung (störungsspezifische Gruppenpsychotherapie versus psychiatrische Einzelgespräche). Der Interviewer ist an den Messzeitpunkten T1, T2, T3, T4 auch „blind“ für diese Behandlungsbedingungen.

4 Behandlungsarme:

Untersuchungsbedingungen:

a. **Behandlungsgruppe: Psychotherapie und Methylphenidat**
Teilnahme an 12 wöchentlichen 2-stündigen Sitzungen einer störungsspezifischen kognitiv-verhaltenstherapeutischen Gruppentherapie; auf die 12 wöchentlichen Gruppensitzungen folgen 10 4-wöchentlichen Sitzungen; Gesamtdauer: 52 Wochen (zusätzlich erfolgt die oben beschriebene Medikation mit Methylphenidat).

b. **Behandlungsgruppe: Psychotherapie und Placebo**
Teilnahme an 12 wöchentlichen 2-stündigen Sitzungen einer störungsspezifischen kognitiv-verhaltenstherapeutischen Gruppentherapie; auf die 12 wöchentlichen Gruppensitzungen folgen 4-wöchentlichen Sitzungen Sitzungen; Gesamtdauer: 52 Wochen (zusätzlich erfolgt die eine Medikation mit Placebo).

Kontrollbedingungen:

c. **Kontrollgruppe: Psychiatrische Gespräche und Methylphenidat**
12 wöchentliche, gefolgt von 4-wöchentlichen psychiatrischen Beratungsterminen über insgesamt 52 Wochen (Vorgehen strukturiert nach schriftlichem Leitfaden, Dauer 15-20 Min./Sitzung) und Medikation mit Methylphenidat.

d. **Kontrollgruppe: Psychiatrische Gespräche und Placebo**
12 wöchentliche, gefolgt von 4-wöchentlichen psychiatrischen Beratungsterminen über insgesamt 52 Wochen (Vorgehen strukturiert nach schriftlichem Leitfaden, Dauer 15-20 Min./Sitzung) und Medikation mit Placebo.

Beginn der Studie: 01.10.2006
Ende der Rekrutierungsphase (voraussichtlich): 30.06.2008
Ende der Nachbeobachtungsphase (voraussichtlich): 30.06.2009
Dauer der Studie pro Patient (d.h. voraussichtliche maximale Behandlungs- und Nachbeobachtungsdauer): 52 Wochen.
Beendigung der Studie: 30.06.2009
Verfügbarkeit der Daten: 31.12.2009
Report/ Publikation: Dezember 2009 ff.

Abbildung 1: Zeitplan der Studie

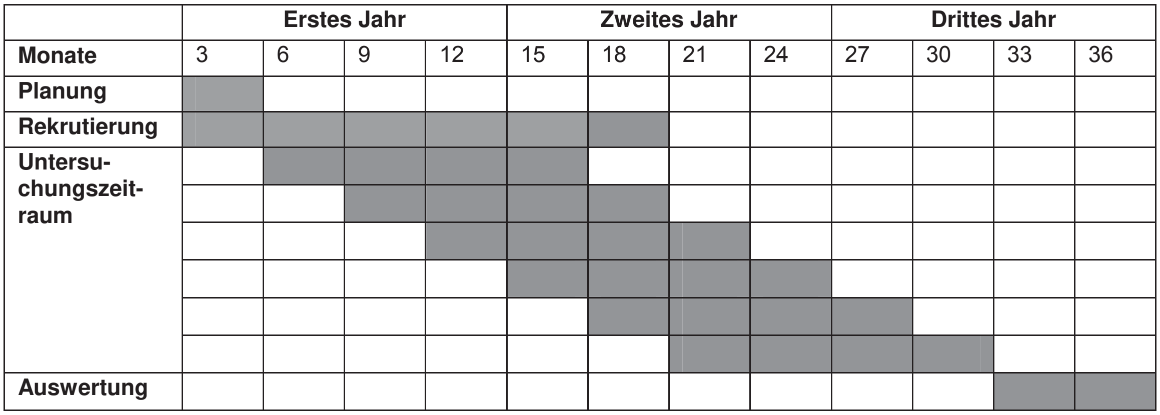

Abbildung 2: Ablauf der Studie

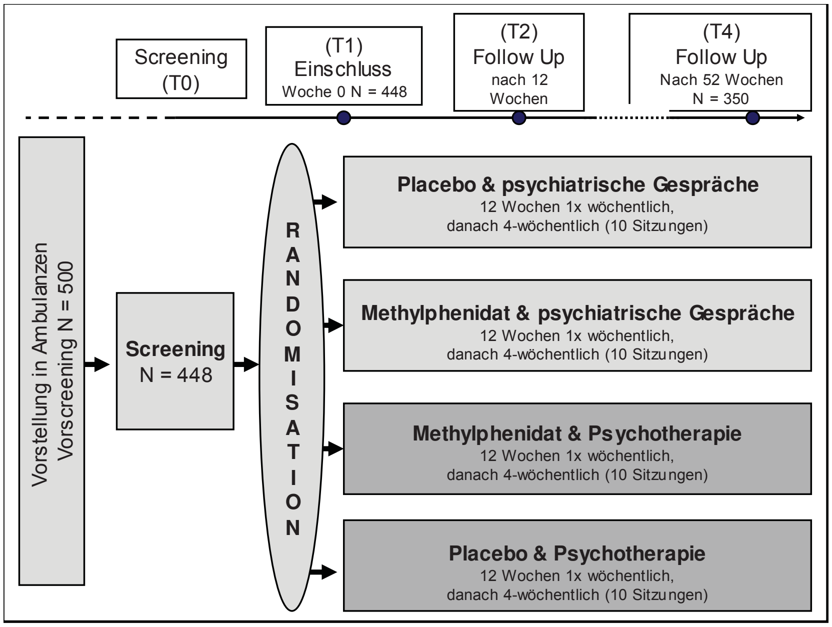

Definition der Abbruchkriterien siehe Kapitel 15.2.

6.2. Teilnehmende Zentren

Es handelt sich um eine Multizenterstudie, in die fünf kooperierende universitäre psychiatrische Kliniken/Institutionen einbezogen sind (**Freiburg, Würzburg, Mannheim, Berlin, Homburg**/**Saar**, siehe Anhang).

6.3 Anzahl aufzunehmender Patienten

Es werden 448 Patienten in die Studie aufgenommen und randomisiert (112 Patienten pro Arm).

7 Studienpopulation

7.1 Charakterisierung der Studienpopulation

Die Patienten müssen die im folgenden aufgeführten Einschlusskriterien aufweisen und dürfen keine der aufgelisteten Ausschlusskriterien zeigen. Die Patienten müssen vor Einschluss in die Studie adäquat über Wesen, Bedeutung und Tragweite der Studie informiert sein und ihr Einverständnis zur Teilnahme erteilt haben.

7.2 Einschlusskriterien

- Männer und Frauen.
- Probanden sprechen fließend Deutsch
- Alter 18 bis einschl. 60 Jahre.
- Diagnosestellung der ADHS nach DSM-IV Kriterien*. 6 von 9 DSM-IV-Kriterien müssen für den unaufmerksamen bzw. hyperaktiv-impulsiven Subtyp erfüllt sein. Für den kombinierten Subtyp sind jeweils 6 von 9 der DSM-IV Kriterien für den unaufmerksamen und hyperaktiv-impulsiven Subtyp gefordert.
- Mehr als 30 Punkte auf der Kurzversion der Wender-Utah-Rating-Scale (Retz-Junginger P et al 2003).
- Retrospektiv persistierende ADHS Symptomatik seit Kindheit.
- Schriftliches Einverständnis der Probanden entsprechend internationalen Guidelines und lokaler Gesetzgebung.
- Unauffällige körperliche Untersuchung (einschl. Blutdruck/Herzfrequenz) ohne schwerwiegenden oder unkontrollierten Befund, Laborwerte im Normbereich (z.B. Blutbild, Nierenretentionswerte, Leberwerte, Schilddrüsenparameter). EKG and EEG ohne pathologisch relevanten Befund).
- Das Screening wurde vollständig durchgeführt, Laborbefunde (einschl. Drogenscreening) liegen nicht länger als 6 Wochen, ggf. Schwangerschaftstest nicht länger als 2 Wochen zurück zum Zeitpunkt der Randomisierung.
- Baselinediagnostik innerhalb von maximal 7 Tagen und Therapiebeginn innerhalb von maximal 14 Tagen nach Randomisierung sind möglich.

*Anmerkung: Die diagnostischen DSM-IV-Kriterien beschreiben die ADHS Symptomatik im Kindesalter. Spezifische diagnostische Kriterien nach DSM-IV oder ICD-10 für ADHS im Erwachsenenalter fehlen bislang. Die klinische Ausprägung der Symptomatik wandelt sich häufig vom Kindes- bis zum Erwachsenenalter (z.B. innere statt zu beobachtende motorische Unruhe). Zur Beurteilung der diagnostischen Kriterien im Erwachsenenalter wird deshalb auch auf die „Stellungnahme zur ADHS–Langfassung" der Bundesärztekammer vom 26.08.2005 auf den Seiten 62 und 63 verwiesen (http://www.bundesaerztekammer.de/30/Richtlinien/ Empfidx/ADHS/Lang/ADHSLang.pdf).

7.3 Ausschlusskriterien

- IQ < 85 entsprechend einem Punktwert von < 17 im Mehrfachwahl Wortschatz Intelligenztest (MWT-B)
- Schizophrenie, bipolar affektive Störung, Borderline Persönlichkeitsstörung, antisoziale Persönlichkeitsstörung, Suizidalität oder Selbstverletzungen, Autismus, motorische Tics, Tourette Syndrom.
- Substanzabusus/-abhängigkeit in den vergangenen 6 Monaten vor Screening. Episodischer Konsum ist kein Ausschlusskriterium. Positiver Drogentest im Screening.
- Neurologische Erkrankungen, Krampfanfälle, pathologischer EEG-Befund (Seitendifferenzen, Herdbefund, epilepsietypische Potentiale), leichtgradige frontotemporale Allgemeinveränderungen sind kein Ausschlusskriteritерium, Glaukom, Diabetes mellitus, Hyperlipidämie (nach Ref.bereichen der

bestimmenden Labors), nicht eingestellte arterielle Hypertonie entsprechend den Empfehlungen der Deutschen Hochdruckliga e.V. DHL® - Deutsche Hypertoniegesellschaft[1] (siehe auch 9.3.1.1), Angina pectoris, bekannte arterielle Verschlusskrankheit oder eine andere Manifestation einer Gefäßbeteiligung, bekannte tachykarde Arrhythmien.

- Schlaganfall in der Vorgeschichte.
- Bekannte vergrößerte Prostata.
- Aktuelle Essstörung (Bulimia nervosa, Anorexia nervosa, Body Mass Index < 19).
- Aktuelle oder geplante Schwangerschaft, Stillzeit. Positiver Schwangerschaftstest im Screening,
- Teilnahme an einer klinischen Prüfung innerhalb der letzten 3 Monate vor Beginn dieser Studie, bzw. gleichzeitige Teilnahme an einer anderen klinischen Prüfung, vorherige Teilnahme an dieser Studie.
- Medikation mit Stimulanzien oder ADHS-spezifische Psychotherapie innerhalb der letzten 6 Monate vor Beginn dieser Studie.
- Bekannte Überempflindlichkeit gegenüber Methylphenidat, anderen sympathomimetischen Arzneimitteln oder einem der sonstigen Bestandteile
- Widerwille oder Unfähigkeit, sich an die Vorgaben des Studienprotokolls zu halten.
- Geschäftsunfähiger Patient, der nicht in der Lage ist, Wesen, Bedeutung und Tragweite der Studie zu verstehen.
- Neben dem Prüfpräparat gemäß Behandlungsbedingung besteht eine andere psychopharmakologische Medikation vor Behandlungsbeginn oder während der Studienteilnahme (Definition nicht zulässiger Medikation und des geforderten Zeitpunkts des Absetzens vor Behandlungsbeginn siehe 4.5).
- Reguläre ambulante Teilnahme an anderer Psychotherapie während der Studienteilnahme
- Aktuelle oder geplante Schwangerschaft, keine Anwendung der definierten Kontrazeptionsmethoden; Stillzeit[2].

[1]Entsprechend den Empfehlungen der Deutschen Hochdruckliga e.V. DHL® - Deutsche Hypertoniegesellschaft gelten Blutdruckwerte bis 129/84 mmHG im Sitzen als normale Blutdruckwerte. Sollte der RR-Wert > 129/84mmHG im Sitzen sein, so erfolgt eine Nachmessung im Sitzen nach 10-15 Minuten. Der RR-Wert wird dann aus dem Mittelwert der beiden RR-Werte im Sitzen ermittelt.
Hochnormale Blutdruckwerte bis 139/89 mmHG können in der Studie nur dann toleriert werden, wenn über die bereits genannten Ausschlusskriterien hinaus auch keine weiteren kardiovaskulären Risikofaktoren vorliegen (Männer > 55 Jahre, Rauchen, Familienanamnese mit frühzeitigen kardiovaskulären Erkrankungen (Männer < 55 Jahre, Frauen < 65 Jahre), Bauchfettleibigkeit Männer ≥ 102 cm, Frauen ≥ 88 cm). RR-Werte ≥ 140/90mmHG sind ein Ausschlusskriterium.

[2]Folgende Frauen können teilnehmen:

- postmenopausale Frauen mit seit mindestens 2 Jahren ausbleibender Menstruation;
- chirurgisch sterilisierte Frauen (Tubenligation, Hysterektomie);
- Frauen mit hoch effektiver Kontrazeption während der gesamten Studiendauer (d.h. von schriftlicher Einverständniserklärung an bis 4 Wochen nach letzter Einnahme der Studienmedikation) und negativem Schwangerschaftstest 2 Wochen vor Randomisierung.

Im Sinne der „note for guidance on non-clinical safety studies for the conduct of clinical trials for pharmaceuticals (CPMP/ICH/286/95 mod)“ werden in der Studie Verhütungsmethoden als hoch effektiv betrachtet, wenn deren Versagerquote bei kontinuierlicher und korrekter Anwendung weniger als 1 % beträgt, wie z.B. Implantate, Injektate, kombinierte orale Kontrazeptiva, gestagenhaltige Intrauterinpessare, sexuelle Abstinenz oder Vasektomie beim Partner.
Eine Interaktion von Medikinet retard® und einer oralen Kontrazeption ist nicht bekannt.

Gebährfähige Frauen müssen darüber aufgeklärt werden, dass sie während der Studie (d.h. vom schriftlichen Einverständnis an bis 4 Wochen nach letzter Einnahme der Studienmedikation) nicht schwanger werden sollten.

8 Aufnahme in die Studie

Studienteilnehmer werden aus dem Patientenstamm der beteiligten Kliniken rekrutiert. Vorgesehen sind 89 bzw. 90 Patienten pro Zentrum zur Randomisierung (112 pro Behandlungsbedingung; Gesamtstichprobe: 448 Patienten). Es sollten demnach pro Zentrum während des Rekrutierungszeitraums 15 Patienten im Quartal in die Studie eingeschlossen werden (siehe auch Flussdiagramm Kapitel 9).

8.1 Zeitpunkt der Aufnahme

Vorgaben: Die Eingangsdiagnostik muss bei Randomisierung abgeschlossen sein, zwischen Randomisierung und Baselinediagnostik (T1) dürfen maximal 7 Tage liegen und zwischen Randomisierung und Behandlungsbeginn maximal 14 Tage. Beträgt der Zeitraum zwischen Screening und Randomisierung mehr als 6 Wochen, müssen vor der Randomisierung Blutentnahme und Drogenscreening innerhalb dieser Frist wiederholt werden (siehe Abbildung). Beträgt der Zeitraum zwischen Schwangerschaftstest und Randomisierung mehr als 2 Wochen, muss dieser vor der Randomisierung innerhalb dieser Frist wiederholt werden (siehe Abbildung). Sollte im Falle einer Protokollverletzung die Frist zwischen Schwangerschaftstest und Behandlungsbeginn mehr als 4 Wochen betragen, so muss der Schwangerschaftstest wiederholt werden (Abbildung 3).

Abbildung 3: Ablauf der Aufnahme in die Studie

Informed consent	Screening	Randomisierung	Baseline (T1)	Behandlungsbeginn
	Laborbefunde (einschl. Drogenscreening) bis maximal 6 Wochen vor Randomisierung, ansonsten Wiederholung<br>Schwangerschaftstest bis maximal 2 Wochen vor Randomisierung, ansonsten Wiederholung		Innerhalb von 7 Tagen nach Randomisierung	Innerhalb von 14 Tagen nach Randomisierung

Der Zeitpunkt des Screenings richtet sich demnach nach dem geplanten Zeitpunkt des Behandlungsbeginns. Dieser ist wegen der geplanten Gruppentherapien davon abhängig, ob am jeweiligen Zentrum die Anzahl der als Probanden in Frage kommenden Patienten groß genug ist, um nach der Randomisierung mit angemessener Gruppengröße (6 bis 9 Probanden) die Therapie beginnen zu können.
Zwischen dem Vorstellungszeitpunkt am jeweiligen Zentrum und dem Einholen der Einwilligung nach Aufklärung bzw. der Eingangsdiagnostik wird kein Zeitintervall vorgeschrieben, da in die Studie nicht nur sich neu vorstellende Patienten eingeschlossen werden dürfen, sondern auch bei Studieninitiierung bekannte Patienten für eine Aufnahme in die Studie in Frage kommen.

8.2 Modus der Aufnahme in die Studie

Für die Teilnahme an der Studie in Frage kommende Patienten werden vom Prüfarzt rekrutiert. Hier erfolgt keine Auswahl bzw. Selektion, d.h. es ist Ziel, möglichst viele Patienten für die Studienteilnahme zu gewinnen.

Die Patienten müssen die im nächsten Kapitel (7.2) aufgeführten Einschlusskriterien aufweisen und dürfen keine der aufgelisteten Ausschlusskriterien zeigen. Die Patienten müssen vor Beginn der Eingangsdiagnostik adäquat über Wesen, Bedeutung und Tragweite der Studie informiert sein und ihr Einverständnis zur Teilnahme erteilt haben.

Es muss das schriftliche Einverständnis von dem Patienten vorliegen, bevor studienspezifische Untersuchungen stattfinden. Liegt die Einwilligung nach Aufklärung vor, wird der Studienteilnehmer auf einer Patientenidentifikationsliste am jeweiligen Prüfzentrum gelistet.

Über diese Liste wird dem Studienteilnehmer konsekutiv eine **Identifikationsnummer** zugewiesen. Diese besteht aus 7 Zeichen:

Die ersten drei Zeichen kodieren das Prüfzentrum.
Die nächsten vier Zeichen, getrennt durch einen Bindestrich, stehen für sukzessiv untersuchte Patienten (Eingangsdiagnostik).

Schlüssel:
Stelle 1 - 3: 001: Würzburg, 002: Freiburg; 003: Mannheim, 004: Homburg, 005: Berlin

Beispiel: 003-0025:. der 25. in Mannheim untersuchte Patient.

Folgende Informationen müssen über den Patienten auf der **Patientenidentifikationliste** festgehalten werden:

- Patientenidentifikationsnummer (Nummer des Patienten)
- Name und Adresse einschließlich Telefonnummer (falls vorhanden)
- Geburtsdatum der Patienten
- Geschlecht des Patienten
- Klinikspezifische Aufnahmenummer des Patienten (optional)
- Patient in Studie randomisiert: ja /nein
- Behandlungsbedingung und Medikationsnummer (Eintrag nach Randomisierung)

Nicht randomisierte Patienten werden in eine **Liste der Screening-Failures** eingetragen. Diese enthält:

- Patientenidentifikationsnummer (Nummer des Patienten)
- Angaben über verletzte Einschlusskriterien
- Angaben über erfüllte Ausschlusskriterien
- Mögliche andere Gründe gegen Randomisierung

Sind die Einschlusskriterien erfüllt, wird ein **Registrierungsformblatt** per FAX an das Zentrum Klinische Studien ZKS Freiburg gesendet.
Die Patientenidentifikationsliste verbleibt im Studienzentrum, die Screening-Failure Liste geht nach Studienende an den Monitor.

Auf diesem Registrierungsformblatt sind folgende Informationen aufgeführt:

- Patientenidentifikationsnummer (Nummer des Patienten)
- Geburtsjahr des Patienten
- Angabe über die Erfüllung der Ein-/Ausschlusskriterien.
- Ist der Patient weiblich und im gebährfähigen Alter ja/nein
- Falls ja, Datum des Schwangerschaftstest

Randomisierungsstelle:

Zentrum Klinische Studien, Universitätsklinik Freiburg
Elsässer Str. 2, D-79110 Freiburg
Fax: 0761-203-6677

Die Randomisierungsstelle am ZKS Freiburg prüft dann die Informationen des Registrierungsformblatts. Dem Patienten wird die Behandlungsbedingung zugewiesen. Die Randomisierungsstelle wird das Prüfzentrum über FAX über die Randomisierung informieren.

Dieses **Randomisierungsfax** enthält:

- Patientenidentifikationsnummer (Nummer des Patienten)
- Geburtsjahr des Patienten
- Behandlungsbedingung: Gruppenpsychotherapie oder psychiatrische Einzelgespräche
- Medikationsnummer

Nach Empfang des Randomisierungsfaxes trägt der Untersucher die Behandlungsbedingung und Medikationsnummer auf der Patientenidentifikationsliste ein und vermerkt die Identifikationsnummer (Zentrums- und Patientennummer) auf dem Notfallumschlag und auf der Prüfware mit der zugehörigen Medikationsnummer.

Methodik der Randomisierung:

Es wird eine nach Zentren stratifizierte Randomisierung durchgeführt in Blöcken mit variabler Länge. Die Blocklängen werden den Prüfärzten nicht bekanntgegeben. Die Randomisierung wird zentral über Fax durchgeführt. Wegen der zu bildenden Therapiegruppen erfolgt die Randomisierung der Patienten zeitlich gruppiert.

Der Ablauf der Aufnahme in die Studie kann wie folgt zusammengefasst werden:

1. Aufklärung und Einholen der Einwilligung nach Aufklärung des für eine Studienteilnahme in Frage kommenden Patienten

2. Listen des Patienten auf der Patientenidentifikationsliste
3. Prüfen der Ein- und Ausschlusskriterien (Eingangsdiagnostik)
4. Randomisierung (nach erfolgter Eingangsdiagnostik und Sicherheit über die Erfüllung der Einschlusskriterien; Laboranalyse innerhalb von max. 6 Wochen vor Randomisierung, ggf. neuer Schwangerschaftstest innerhalb von max. 2 Wochen vor Randomisierung)
5. Baselinediagnostik T1 (max. 7 Tage nach Randomisierung)
6. Therapiebeginn (max. 14 Tage nach Randomisierung)

9 Methode

9.1 Zeitplan der Studie

Flussdiagramm zum Studienverlauf

	1. Jahr				2. Jahr				3. Jahr			
**Monate**	3	6	9	12	3	6	9	12	3	6	9	12
**Planung**												
**Rekrutierung**	84	84	84	84	84	84						
**Studien-behandlung**		75	…	…	…							
			75	…	…	…						
				75	…	…	…					
					75	…	…	…				
						75	…	…	…			
							75	…	…	…		
**Auswertung**												

Der Prüfarzt informiert den Monitor 4 Wochen nach Studienende über weitere Ereignisse (UEs, sUEs, uUEs), die indirekt oder direkt mit der klinischen Prüfung im Zusammenhang stehen und/oder für die Beurteilung der Studienergebnisse relevant sein könnten.

9.2 Behandlung

9.2.1 Studienbehandlung

Die im Rahmen der Studie durchgeführte **Gruppenpsychotherapie** zur Behandlung der ADHS richtet sich nach dem kognitiv-verhaltenstherapeutischen Arbeitsbuch „Psychotherapie der ADHS im Erwachsenenalter – ein Arbeitsbuch" von Heßlinger et al. (2004). Diesem Ansatz liegt die dialektisch-behaviorale Therapie der Borderline-Persönlichkeitsstörung (BPS) zu Grunde (Linehan, 1996), da zum Teil ähnliche Schwierigkeiten bei Patienten mit BPD und ADHS vorliegen (z.B. Impulsivität, Stimmungslabilität, instabile und streitbelastete soziale Beziehungen, Inkonstanz im Verfolgen von Zielen). Das Programm wurde jedoch spezifisch auf die ADHS ausgerichtet. Wesentliches Ziel der Therapie ist die Kontrolle der ADHS. Das Vorgehen ist strukturiert mit symptomorientierten Modulen. Diese Module decken die wesentlichen Symptome und typischen Verhaltensweisen von Erwachsenen mit ADHS ab. Um flexibel auf die Bedürfnisse der Patienten reagieren zu können, kann die Reihenfolge der Module variabel sein. Durchgeführt wird die Behandlung als Gruppentherapie mit 6–9 Teilnehmern in wöchentlichen Sitzungen von je 2 Stunden Dauer. Die Gruppe findet in den ersten 12 Wochen wöchentlich, danach 4-wöchentlich statt. Hausaufgaben sind wesentlicher Bestandteil der Therapie. Die Inhalte der Sitzungen sind folgende:

1. Einführung und Klärung, Informationen zur ADHS.
2. Informationen zur ADHS, Achtsamkeitstraining (auch gerichtet auf Aufmerksamkeits- und Konzentrationsschwierigkeiten).
3. Achtsamkeitstraining, Alltagstransfer.
4. Kontrolle von Desorganisiertheit: Zeitplanung, Organisationsplanung, Merkhilfen, Hilfestellungen, Umgebung.

5. Verhaltensanalyse: Teilnehmer erlernen Verhaltensanalysen (Beschreibung des Problemverhaltens im Detail, typische Situationen, vorausgehende Bedingungen, kurz- und langfristige Konsequenzen, alternative Problemlösestrategien, vorbeugende Maßnahmen, Wiedergutmachung).
6. Verhaltensanalyse: Verhaltensanalysen in Eigenregie durchführen.
7. Gefühlsregulation: Einführung in Theorie der Gefühle (Primäremotionen, Signal- und Kommunikationscharakter von Emotionen, Beziehung von Emotionen zu Kognitionen, Körperwahrnehmungen und Verhalten, Übungen zur Emotionswahrnehmung und Emotionsregulation, Kontrolle von Wut und Ärger).
8. Information über Symptome und Behandlungsmöglichkeiten bei Depression, Information über medikamentöse Behandlungsmöglichkeiten bei ADHS, Wirkungen und Nebenwirkungen, Erfahrungsaustausch.
9. Impulskontrolle: Verhaltensanalysen bezüglich Impulskontrollstörungen, kurz- und langfristige Konsequenzen von Impulsivität, typische Situationen, zielorientiertes Verhalten erlernen.
10. Streßmanagement: Zusammenhang von desorganisiertem Verhalten mit subjektivem Erleben von Streß, Stress-Leistungs-Kurve, Stressmanagement, Sport.
11. Sucht: Information zu Komorbidität bei ADHS, Reflektion eigenen süchtigen Verhaltens, kurz- u. langfristige Konsequenzen, Indikationen für Alternativverhalten bzw. Therapie.
12. Auswirkungen von ADHS auf Beziehungen, Selbstachtung; Reflektion und Austausch.

In der **Kontrollgruppe** werden in den ersten 12 Wochen wöchentlich, danach 4-wöchentlich psychiatrische Einzelgespräche (ca. 15-20 Min) durchgeführt. Ziel der Gespräche ist es, die Wirkung der Medikation zu überprüfen und die Dosis möglichst optimal anzupassen. Der Ablauf und Inhalt dieser Gespräche richtet sich nach einem schriftlich festgelegten Leitfaden:
Begrüßung, Aktuelles, Wirkung der Studienmedikation auf die ADHS Symptomatik (Unaufmerksamkeit und Hyperaktivität/Impulsivität), unerwünschte Wirkungen (nach AMDP, z.B. Schlafstörungen, Appetit, gastrointestinale Beschwerden, Tachykardie, Schwitzen, Ängste, Reizbarkeit), Erhebung des psychopathologischen Befundes, Dosisanpassung, weitere aktuelle Fragen, Verabschiedung.

9.2.2 Definition der Begleitmedikation/zusätzliche Behandlungsmethoden

Nicht erlaubte Komedikation siehe unter 4.5. Die Einnahme der unter 4.5. genannten Medikamente führt zum Abbruch der medikamentösen Studienbehandlung. Übrige Begleitmedikation (verschreibungspflichtig und nicht verschreibungspflichtig), die während der Studie eingenommen wurde, muss im CRF und in der Patientenakte dokumentiert werden (Indikation, Dosis, Applikationsform, Dauer der Einnahme).

Es werden keine zusätzlichen Behandlungsmethoden angewandt, die nicht Gegenstand der Studienbehandlung sind. Eine begleitende Einzelpsychotherapie außerhalb der Studie ist nicht erlaubt. Sollte sich ein Patient bereits in psychotherapeutischer Behandlung befinden, so kann diese Behandlung während der Studienteilnahme unterbrochen werden, wenn dies klinisch vertretbar ist.

Für die Patienten der Behandlungsgruppe besteht die Möglichkeit, begleitend zur Gruppentherapie Einzelgesprächstermine wahrzunehmen, um etwaige Probleme oder Themen aufgreifen zu können, die die Patienten in der Gruppe nicht besprechen wollen. Dafür sind in den ersten 12 Wochen 1 Termin und in den folgenden 9 Monaten 2 Termine à ca. 30-45 Minuten möglich.

9.3 Untersuchungen

9.3.1 Eingangsdiagnostik (Screening)

Die Eingangsdiagnostik bildet die Grundlage für die Beurteilung der Ein- und Ausschlusskriterien (siehe auch Flussdiagramm 9.4).
Sie erfolgt maximal 6 Wochen vor der Randomisierung und gestaltet sich für alle Patienten gleich. Sie beinhaltet diagnostische Interviews bzw. Selbstbeurteilungsverfahren und einen IQ-Kurztest.
Weiterhin werden eine körperlich-neurologische Untersuchung (siehe 9.3.1.1), ein EEG und EKG sowie die beschriebenen Blut- und Urinuntersuchungen (siehe 9.3.1.2) durchgeführt. Vorraussetzung für die Randomisierung ist u.a. ein negativer Schwangerschaftstest (vgl. Ein- und Ausschlusskriterien Kapitel 7.2 und 7.3). Bei Randomisierung darf der Schwangerschaftstest nicht länger als 2 Wochen zurückliegen, ansonsten muss er wiederholt werden.

Folgende demographische Daten werden vom Prüfarzt erfragt und in CRF und Patientenakte dokumentiert:
Geburtsdatum, Alter, Geschlecht, Familienstand, Anzahl der Kinder, ethnische Zugehörigkeit, höchster erreichter Schulabschluss, berufliche Tätigkeit.
Weiterhin werden ältere und aktuelle Erkrankungen sowie vorangegangene psychiatrische Hospitalisierungen (Alter bei Hospitalisierung, Dauer) erfragt.

Dabei wird auch ein **strukturiertes klinisches Interview für DSM-IV**, **SKID I+II (First et al. 1997)** durchgeführt: (Hogrefe 1997). Das SKID ist ein international und im deutschen Sprachraum etabliertes Verfahren. Im Rahmen der Studie dient es der Feststellung von Komorbiditäten, die für die Ausschlusskriterien und die Stichprobenbeschreibung von Bedeutung sind.
SKID-I und SKID-II dient zur Diagnose psychischer Störungen (Achse I) und Persönlichkeitsstörungen (Achse-II) nach DSM-IV. Alle Diagnosen werden aktuell und lifetime mit Zusatzinformationen über Beginn und Verlauf erfaßt. Berücksichtigte Störungen im SKID-I: Affektive Störungen, Psychotische Störungen, Störungen durch psychotrope Substanzen, Angststörungen, somatoforme Störungen, Essstörungen und Anpassungsstörungen.
SKID-II: im DSM-IV definierte Persönlichkeitsstörungen werden berücksichtigt.

Zur retrospektiven Selbstbeurteilung kindlicher ADHS-Symptome wird die **Wender-Utah-Rating-Scale (WURS-k,** deutsche Kurzform der Wender-Utah-Rating-Scale, Retz-Junginger et al. 2002) eingesetzt.

Fragebogen zur Gruppenpsychotherapieevaluation (Hesslinger et al. 2002):
Selbsteinschätzung der Wirksamkeit der Gruppenpsychotherapie und deren Module auf die ADHS Symptomatik durch den Patienten.

Die übrigen Interviews und Selbstbeurteilungsinstrumente sind unter dem Kapitel „Zielkriterien“ (Kapitel 10.1) spezifiziert.

9.3.1.1 Spezifizierung der körperlichen Untersuchung

Gründliche internistische und neurologische körperliche Untersuchung (Motorik, Sensibilität, Hirnnervenstatus, Koordination, Feinmotorik), Körpergröße, -gewicht, Vitalparameter (Blutdruck und Puls).
Der Blutdruck wird, nachdem der Patient in einem ruhigen Raum einige Minuten Platz genommen hat, im Sitzen gemessen. Die 2. Messung erfolgt 5 Minuten nach dem Aufstehen. Die Herzfrequenzmessung erfolgt nach der 2. Messung des Blutdrucks wieder im Sitzen.

9.3.1.2 Spezifizierung der Laboruntersuchungen

Blutuntersuchungen: Entnahme von ca. 20 ml Blut für Serummonovette, EDTA-Monovette, Citratmonovette, und Fluoridmonovette. Die Sicherheitslaborparameter werden von den jeweiligen Laboren der Prüfzentren analysiert:
Kleines Blutbild (Hb, HKT, Erythrozyten, Leukozyten, Thrombozyten, MCV).
Serumchemie: Leberparameter (GOT, GPT, Gamma-GT), Nierenretentionswerte (Kreatinin, Harnstoff), Harnsäure, Kalium, Natrium, Kalzium, Gesamteinweiß, Albumin, Alkalische Phosphatase, Cholinesterase, Cholesterin, Triglyceride, LDL- und HDL-Cholesterin, Kreatininkinase, TSH, Gesamtbilirubin, Nüchtern-Glucose. PTT und Quick.
Urinuntersuchungen: Drogenscreening (Cannabis, Amphetamine, Ecstasy, Cocain, Barbiturate, Opiate, Benzodiazepine, Trizyklika).
Bei Frauen Schwangerschaftstest (Beta-HCG).

9.3.1.3 Spezielle Untersuchungen

EEG und EKG, Auswertung erfolgt nach visuellen Kriterien.
Neurobiologische Zusatzuntersuchungen (cerebrale Kernspintomographie und genetische Analysen siehe Anhang Kapitel 18.1 und 18.2).

9.3.2 Zeitliche Abfolge der Untersuchungen in der Studienbehandlungsphase

Im Folgenden werden die Untersuchungen in ihrer exakten zeitlichen Folge aufgeführt.

Tag 0 (Woche 0; 0 bis 7 Tage nach Randomisierung)
Visite T1 (siehe Flussdiagramme in 9.4)

Tag 7 (± 1 Tag) (Woche 1)
Blutdruck, Puls, Erfassung unerwünschter Ereignisse, Änderung der Begleitmedikation, Ausgabe des Studienpräparates (ab Woche 2 zusätzlich Kontrolle der eingenommenen Medikation: „Pill-Counting“).

Tag 14 (± 2 Tag) (Woche 2)
Entspricht Woche 1.

Tag 21 (± 2 Tag) (Woche 3)
Entspricht Woche 1.

Tag 28 (± 2 Tag) (Woche 4)
Entspricht Woche 1.

Tag 35 (± 2 Tag) (Woche 5)
Entspricht Woche 1.

Tag 42 (± 2 Tag) (Woche 6)
Entspricht Woche 1.
Zusätzlich: Körpergewicht.

Tag 49 (± 2 Tag) (Woche 7)
Entspricht Woche 1.

Tag 56 (± 2 Tag) (Woche 8)
Entspricht Woche 1. **Zusätzlich: Minivisite t2** (siehe Flussdiagramme in 9.4)

Tag 63 (± 2 Tag) (Woche 9)
Entspricht Woche 1.

Tag 70 (± 2 Tag) (Woche 10)
Entspricht Woche 1.

Tag 77 (± 2 Tag) (Woche 11)
Entspricht Woche 1.

Tag 84 (± 2 Tag) (Woche 12)
Entspricht Woche 1.
Zusätzlich: Laboruntersuchungen (siehe 9.3.1.2 Blutuntersuchungen), Körpergewicht.

Tag 91 (± 2 Tag) (Woche 13)
Visite T2 (siehe Flussdiagramme in 9.4)

Tag 112 (± 4 Tage) (Woche 16)
Entspricht Woche 1.

Tag 140 (± 4 Tage) (Woche 20)
Entspricht Woche 1. **Zusätzlich:** Körpergewicht.
Minivisite t3 (siehe Flussdiagramme in 9.4)

Tag 168 (± 4 Tage) (Woche 24)
Entspricht Woche 1.
Zusätzlich: EKG, Schwangerschaftstest, Drogenscreening (siehe 9.3.1.2 Urinuntersuchungen).

Tag 182 (± 2 Tag) (Woche 26)
Visite T3 (siehe Flussdiagramme in 9.4)

Tag 196 (± 4 Tage) (Woche 28)
Entspricht Woche 1.
Zusätzlich: Laboruntersuchungen, Körpergewicht.

Tag 224 (± 4 Tage) (Woche 32)
Entspricht Woche 1.

Tag 252 (± 4 Tage) (Woche 36)
Entspricht Woche 1.

Tag 280 (± 4 Tage) (Woche 40)
Entspricht Woche 1. **Zusätzlich:** Laboruntersuchungen (siehe 9.3.1.2 Blutuntersuchungen), Körpergewicht.
Minivisite t4 (siehe Flussdiagramme in 9.4)

Tag 308 (± 4 Tage) (Woche 44)
Entspricht Woche 1.

Tag 336 (± 4 Tage) (Woche 48)
Entspricht Woche 1.

Tag 364 (± 4 Tage) (Woche 52)
Entspricht Woche 1.
Zusätzlich: Laboruntersuchungen (siehe 9.3.1.2 Blutuntersuchungen), Schwangerschaftstest (siehe 9.3.1.2), Körpergewicht. **Visite T4** (siehe Flussdiagramme in 9.4).

Sollte ein Patient zum Untersuchungstermin nicht erscheinen, kontaktiert der Prüfarzt den Patienten und fragt nach den Gründen für das Nichterscheinen. Nach Möglichkeit sollte die fehlende Untersuchung so schnell wie möglich nachgeholt werden.

9.3.3 Nachuntersuchungen

4 Wochen nach Studienende (Woche 56) werden die Patienten telefonisch vom Prüfarzt zur Nachverfolgung bekannter unerwünschter Ereignisse und zur Erfragung neu aufgetretener unerwünschter Ereignisse telefonisch kontaktiert.

9.3.4 Abschlussuntersuchung

Umfang der Abschlussuntersuchung siehe 9.4. Sollte ein Patient früher als vorgesehen aus der Studie ausscheiden, so soll nach Möglichkeit die Abschlussuntersuchung (ensprechend Woche 52) durchgeführt werden. Die Nachuntersuchung enspricht 9.3.3.

Bei der Abschluss- und Nachuntersuchung wird die Indikation zu weiteren Behandlungsmaßnahmen vom Prüfarzt geprüft. Bei vorhandener Indikation ist diese dem Patienten mitzuteilen. Das Prüfzentrum unterbreitet bei Indikation ein Behandlungsabgebot oder ist bei der Organisation einer Weiterbehandlung behilflich. Modalitäten der Weiterbehandlung sind in der Krankengeschichte zu dokumentieren.

Dieses Vorgehen gilt auch auch für vorzeitig aus der Studie ausscheidende Patienten (siehe Kapitel 15.2.2).

9.3.5 Videoaufzeichnungen

Diagnostische Interviews sowie die psychotherapeutischen Interventionen werden videographiert, um studieninterne Qualitätskontrollen zu ermöglichen (Bestimmung von Beurteiler-Übereinstimmungen und Sicherstellung manualtreuer Behandlungen). Videobänder werden pseudonymisiert aufbewahrt. Die Kontrollbehandlung (psychiatrische Einzelgespräche wird audiographiert und auch pseudonymisiert aufbewahrt. Die Einwilligung der Patienten nach Aufklärung über die anzufertigenden Video- und Audioaufzeichnungen wird vor Studienbeginn schriftlich eingeholt.

Um Beurteiler-Übereinstimmungen bestimmen zu können, wird auf den CRFs von Interviews bzw. Fremdbeurteilungsskalen der Name des Beurteilers festgehalten.

9.4 Flussdiagramm Studienablauf

**Flussdiagramm**	**Behandlungsdauer (52 Wochen)**							
4-armiges Studiendesign<br>n = 112/Behandlungsarm			Erste 12 Wochen: wöchentliche Gruppensitzungen / psychiatrische Einzelgespräche		Woche 13-52<br>4-wöchentliche Gruppensitzungen/ psychiatrische Einzelgespräche			
		**T1**	**t2**	**T2**	**t3**	**T3**	**t4**	**T4**
**Messinstrumente**	**Eingangsdiagnostik (Screening)**	**Studienvisite Baseline (Woche 0)**	**Minivisite (Woche 8)** (to allow for LOCF)	**Studienvisite (Woche 13)**	**Minivisite (Woche 20)** (to allow for LOCF)	**Studienvisite (Woche 26)**	**Minivisite (Woche 40)** (to allow for LOCF)	**Studienvisite (Woche 52)**
**Symptomatik und Therapieevaluation:**								
SKID-I	X							
SKID-II	X							
Mehrfachwortwahltest (MWT)	X							
ADHS-Checkliste	X	X		X		X		X
Wender-Utah Rating Scale (WURS-25)	X							
CAARS Selbsteinschätzung		X	X	X	X	X	X	X
CAARS Fremdeinschätzung, (verblindet)		X		X		X		X
Allgemeine Psycho-pathologie (SCL-90-R)		X		X		X		X
Depression (BDI)		X		X		X		X
Lebensqualität (Q-LES-Q)		X		X		X		X
Allgemeiner Symptom-schweregrad (CGI), Fremd-einschätzung (verblindet)		X		X		X		X
Nikotin-/Koffeinkonsum, nicht stoffgebundene Süchte	Woche 0, 6, 12, 20, 28, 40, 52							
Fehltage am Arbeitsplatz	Woche 0-12 wöchentlich, Woche 13-52 4-wöchentlich							
Fragebogen zur Therapieevaluation								X
**Sicherheitsparameter:**								
Körpergröße	X							
EEG	X							
Körperliche Untersuchung	X							
Gewichtskontrolle	Eingangsdiagnostik, Woche 6, 12, 20, 28, 40, 52							
Blutentnahmen (Routine)	Eingangsdiagnostik, Woche 12, 28, 40, 52							
Drogenscreening	Eingangsdiagnostik, Woche 24							
Schwangerschaftstest*	Eingangsdiagnostik, Woche 24, 52							
EKG	Eingangsdiagnostik, Woche 24							
Vitalparameter	Eingangsdiagnostik, Woche 0-12 wöchentlich, Woche 13-52 4-wöchentlich							
Prüfmedikationsausgabe und -rücknahme	Woche 0-12 wöchentlich, Woche 13-52 4-wöchentlich							
**Zusatzuntersuchungen:**								
Cerebrale MRT		X		X				X
Genetik	X							

**** Wiederholung des Schwangerschaftstests, falls dieser bei Beginn der Studienbehandlung mehr als 4 Wochen zurückliegt (siehe auch Kapitel 8.1)***

10 Zielkriterien zur Beurteilung von Wirksamkeit und Verträglichkeit

10.1 Wirksamkeit

Primäres Zielkriterium:

Conners Adult ADHD Rating Scale (CAARS-O:L, blind-observer rated, 66 Fragen, Conners et al. 1999):
ADHS –Symptomatik nach Fremdeinschätzung gemessen mit dem Summenscore der Conners Adult ADHD Rating Scale, Vergleich T1/T2.
Die CAARS erfüllt alle notwendigen Voraussetzungen an ein zuverlässiges und gültiges Selbstrating bzgl. der ADHS-Symptomatik bei Erwachsenen. Er liefert ein quantitatives Maß der gegenwärtigen ADHS-Symptomatik. Die Langform des Fremdbeurteilungsverfahrens (CAARS-S:L) hat 66 Einzelitems und enthält 9 empirisch abgeleitete Skalen, die ausführlich das Problemverhalten charakterisieren. Sie wird vom „blinden" Interviewer ausgefüllt und im Rahmen der Studie zur Veränderungsmessung eingesetzt.

Sekundäre Zielkriterien:

CAARS-O:L: nach „blinder" Fremdeinschätzung (siehe primäres Zielkriterium).Vergleich T1/T3, T1/T4.

Subskalen der CAARS-O:L: nach „blinder" Fremdeinschätzung. Vergleich T1/T2, T1/T3, T1/T4.

Conners Adult ADHD Rating Scale (CAARS-S:L – Self-report Scale Long Version, Conners et al. 1999): siehe primäres Zielkriterium, nach Selbsteinschätzung, Summenscore und Subskalen Vergleich T1/T2, T1/T3, T1/T4.

ADHS-Checkliste (ADHS-DC, Rösler et al. 2004): Validierter Fragebogen zur Fremdeinschätzung des Ausmasses der diagnostischen Kriterien für ADHS/ADS im Erwachsenenalter nach DSM-IV. Insgesamt 18 Fragen (9 für Hyperaktivität/Impulsivität, 9 für Unaufmerksamkeit). Vergleich T1/T2, T1/T3, T1/T4.

Allgemeine Psychopathologie (SCL-90-R, Derogatis 1977): Symptom-Checkliste, deutsch: Franke 2002. Die SCL-90-R ist ein Selbstbeurteilungsverfahren zur Erfassung körperlicher und psychischer Symptome durch körperliche und psychische Symptome zeitlich bezogen auf den Tag der Beurteilung und die zurückliegende Woche. Es exisiteren drei übergeordnete Indices zur Profilbeschreibung und 9 Syndromskalen zur Erfassung folgender Bereiche: Somatisierung, Zwanghaftigkeit, Unsicherheit im Sozialkontakt, Depressivität, Ängstlichkeit, Aggressivität/ Feindseligkeit, Phobische Angst, Paranoides Denken und Psychotizismus. Das Verfahren ist international und im deutschen Sprachraum gut eingeführt und umfassend hinsichtlich gängiger Gütekriterien untersucht). Vergleich T1/T2, T1/T3, T1/T4.

Beck Depressions Inventar (BDI, Beck 1961): Das Beck-Depressions-Inventar ist ein national und international verbreitetes und in klinischen Zusammenhängen erfolgreich eingesetztes Selbstbeurteilungs-instrument zur Erfassung des Schweregrades einer depressiven Symptomatik (21 Fragen). Vergleich T1/T2, T1/T3, T1/T4.

Clinical Global Impression (CGI, Guy 1976): Allgemeiner klinischer Eindruck. Erfasst wird der allgemeine Schweregrad (0-7) sowie die allgemeine Änderung nach Fremdeinschätzung (0-7). Vergleich T1/T2, T1/T3, T1/T4.

Lebensqualität (Quality of Life Enjoyment and Satisfaction Questionnaire, Q-LES-Q, Endicott et al. 1993) : 91 Fragen zu Lebensqualität und – zufriedenheit. Erfasst werden die Bereiche: körperliche Gesundheit, Emotionalität, Arbeitsplatz, Haushalt/Alltag, Schule/Ausbildung, Freizeit, Soziale Beziehungen, allgemeine Zufriedenheit.). Vergleich T1/T2, T1/T3, T1/T4.

Weitere sekundäre Zielkriterien:

- Fehlzeiten am Arbeits- bzw. Ausbildungsplatz (in Tagen) während der Studienbehandlung in den 4 Behandlungsbedingungen. Erfassung an den Visiten wöchentlich von Woche 1-12, 4-wöchentlich von Woche 13-52.
- Falls zutreffend: Menge des Nikotin-/Koffeinkonsums in den vergangenen 7 Tagen (Zig/d; durchschnittlich ml/d), Ausmass nicht-stoffgebundener Süchte in den vergangenen 7 Tagen (durchschnittlich Stunden/d). Vergleich T1/T2, T1/T3, T1/T4.
- Bestimmung neurobiologischer (cerebrale MRT) und genetischer Marker für Therapieresponse (siehe Anhang Kapitel 18).
- Weitere explorative Datenanalyse vorgesehen.

10.2 Verträglichkeit

Die Sicherheit und Verträglichkeit werden während der Studienbehandlung auf der Basis der berichteten unerwünschten Ereignisse, der Laborparameter, Vitalparameter (Blutdruck, Puls), des Körpergewichts und des EKGs beurteilt (siehe auch Kapitel 9.3 und 9.4).

Alkohol-, Drogen- und Medikamentenabusus
Die Erfassung der Konsumgewohnheiten von Drogen, Medikamenten und Alkohol erfolgt bei der Eingangsdiagnostik mit dem SKID-I Interview, das speziell zur Diagnostik von Achse I Störungen nach DSM-IV und auch für ICD-10 entwickelt worden ist. Es handelt sich um ein eingeführtes strukturiertes Untersuchungsinstrument. Drogenscreening im Urin im Rahmen der Eingangsdiagnostik und im Verlauf in Woche 24.
Die Begleitmedikation wird in CRF und Patientenakte dokumentiert.

Unerwünschte Ereignisse bzw. unerwünschte Arzneimittelwirkungen werden kontinuierlich während der Studie, d.h. in den ersten 12 Wochen wöchentlich bei jeder Visite, anschließend bis Woche 52 4-wöchentlich registriert und in CRF und Patientenakte eingetragen.

Relevante oder pathologische Laborparameter
Bei relevanten und pathologischen Laborparametern wird spätestens nach 1 Woche eine Kontrolluntersuchung durchgeführt. Sollte dann noch keine Normalisierung der Werte eingetreten sein, werden Laborwertkontrollen bis zur Normalisierung oder Klärung der Ursache wöchentlich durchgeführt. Der Prüfarzt entscheidet über einen eventuellen Abbruch der Studienmedikation oder der gesamten Studie bei diesem Patienten und ggf. über weitere Untersuchungen.
Vor Studienbeginn ist vom untersuchenden Labor eine Tabelle der Referenzbereiche mit Beschreibung der Methodik zur Verfügung zu stellen. Ändern sich während der Studie die Referenzbereiche der Routine-Laborparameter oder der speziell für die Studie durchgeführten Laboruntersuchungen, so ist dies dem Monitor mitzuteilen. Am Ende der Studie ist erneut eine Tabelle mit den aktuellen Referenzbereichen zur Verfügung zu stellen. Das untersuchende Labor unterliegt einer regelmäßigen Qualitätssicherung.

10.2.1 Definition und Dokumentation unerwünschter Ereignisse (UE)

Unerwünschte Ereignisse sind **alle** im Rahmen der Studie beobachtete unerwünschte medizinische Ereignisse, die bei einem Patienten nach Durchführung der einzelnen Behandlungen auftreten.
Unerwünschte Ereignisse, die der Patient spontan meldet oder der Prüfarzt beobachtet, werden unabhängig vom ursächlichen Zusammenhang kontinuierlich während der klinischen Prüfung und bei jeder Kontrollvisite registriert sowie in der Krankenakte und in den dafür vorgesehenen Prüfbogen (UE-Bogen) im CRF eingetragen.

Alle unerwünschten Ereignisse, gleich welcher Intensität, werden vom Prüfarzt überwacht, bis sie ausreichend abgeklungen oder vollständig charakterisiert sind.

10.2.2 Definition und Dokumentation schwerwiegender unerwünschter Ereignisse (SUE)

Ein **schwerwiegendes unerwünschtes Ereignis** ist jedes unerwünschte medizinische Ereignis, das

- zum Tode des Patienten führt,
- lebensbedrohlich ist,
- eine stationäre Behandlung des Prüfungsteilnehmers oder eine Verlängerung des stationären Aufenthaltes erforderlich macht,
- zu bleibenden oder signifikanten Schäden/Behinderungen führt, oder
- eine angeborene Mißbildung bzw. einen Geburtsfehler darstellt.

Andere Zustände können auch als schwerwiegend eingestuft werden, wenn sie nach Ansicht des Prüfarztes nicht unmittelbar lebensbedrohlich sind oder zu einer stationären Aufnahme führen, aber die Sicherheit des Patienten gefährden oder nur durch Eingreifen eines der oben genannten Ereignisse abgewendet werden kann.
Der Prüfarzt hat jedes schwerwiegende unerwünschte Ereignis, das während der Studie oder während 30 Tagen nach Ausscheiden des Patienten aus der Studie eintritt, auf dem Formblatt **„Schwerwiegendes Unerwünschtes Ereignis (SUE-Bogen)“** zu dokumentieren und **innerhalb von 24 Stunden** an den Leiter der Klinischen Prüfung sowie an den Hersteller der Prüfmedikation zu melden. Die Meldung muß per Telefax an die folgenden beiden Adressen erfolgen:

**LKP Dr. med. Alexandra Philipsen**<br>Universitätsklinikum Freiburg<br>Abteilung für Psychiatrie und Psychotherapie<br>Hauptstrasse 5, D-79204 Freiburg<br>**FAX: 0761-270-6619**<br>Telefon: 0761-270-6501
**Fa. Medice Arzneimittel Pütter GmbH & Co.KG**<br>**z.Hd. Frau Dühme**<br>Kuhloweg 37, D- 58638 Iserlohn<br>**FAX: 02371-937-360**<br>Telefon: 02371-937-203

Die Dokumentation des SUE erfolgt sowohl auf der entsprechenden Seite der Patientenerhebungsunterlagen (UE-Bogen), als auch auf dem SUE-Bogen.

Im unerwarteten Todesfall oder bei lebensbedrohlichem SUE muß der SUE-Bogen zusammen mit einer Epikrise innerhalb von 3 Arbeitstagen, bei nichtlebensbedrohlichem SUE innerhalb von 10 Arbeitstagen, an den LKP geschickt werden.

Wenn das SUE die Sicherheit der Patienten oder die Durchführung der Studie beeinträchtigen könnte, werden ebenfalls die Ethikkommissionen informiert. Über einen Abbruch der Studienbehandlung und therapeutische Maßnahmen entscheidet der Prüfarzt.

Alle UE und SUE müssen nach GCP verfolgt werden, bis sie behoben sind oder nicht mehr klinisch relevant oder vollständig charakterisiert sind.

10.2.3 Meldepflichten von SUE

Der Sponsor muss bei unerwartetem Tod oder unerwartetem lebensbedrohlichen Ereignis, die mit der Studienmedikation zusammenhängen könnten, innerhalb von **7 Tagen** nach Kenntnis die Bundesoberbehörde und die Ethikkommission informieren, und innerhalb von **8 Tagen** relevante Informationen nachsenden.

Bei allen anderen unerwarteten schwerwiegenden Ereignissen, die mit der Studienmedikation zusammenhängen könnten, muss der Sponsor die Bundesoberbehörde und die Ethikkommission innerhalb von **15 Tagen** nach Kenntnis informieren.

10.2.4 Klassifizierung unerwünschter Ereignisse

Für jedes unerwünschte Ereignis müssen folgende Informationen erfasst werden:

- Die **Intensität** unerwünschter Ereignisse ist wie folgt einzustufen:
- **Beschreibung der Symptome:** Falls möglich wird eine Diagnose angegeben. Falls keine Diagnose gestellt werden kann, sollen Symptome angegeben werden, z.B. Übelkeit.
- **Beginn** Datum und Zeitpunkt: Bei veränderten Laborwerten erstes Auftreten des pathologischen Wertes.
- **Ende** Datum und Zeitpunkt: Bei veränderten Laborwerten Zeitpunkt der Normalisierung des Parameters.
- **Beurteilung des Kausalzusammenhanges:**

Ziel ist es, möglichst immer den Kausalzusammenhang von unerwünschter Ereignisse zu bestimmen.

Dabei wird jedes unerwünschte Ereignis vom Prüfarzt entweder als mit der Studienbehandlung (Studienmedikation, Gruppenpsychotherapie, Kontrollbehandlung (psychiatrische Gespräche)) in Zusammenhang stehend oder nicht mit der Studienbehandlung in Zusammenhang stehend beurteilt. Dabei ordnet der Prüfarzt das UE hinsichtlich der Kausalität den zwei folgenden Kategorien zu:

- **UE mit Studienbehandlung** (wahlweise Studienmedikation, Gruppenpsychotherapie, Kontrollbehandlung (psychiatrische Gespräche)) in **Zusammenhang stehend.**
- **UE nicht mit Studienbehandlung** (wahlweise Studienmedikation, Gruppenpsychotherapie, Kontrollbehandlung (psychiatrische Gespräche)) **in Zusammenhang stehend.**

Entsprechend der Empfehlung der WHO wird auf eine Einstufung des Kausalzusammenhanges in „sicher, wahrscheinlich, möglich, keiner oder nicht beurteilbar“ verzichtet (World Health Organization (WHO) - Council for International Organizations of Medical Sciences (CIOMS) (2005) Management of Safety Information from Clinical Trials. Report of the CIOMS Working Group VI. CIOMS, Geneva:84-86).

- **Maßnahmen**
- **Ergebnis für den Patienten**
- **Kommentare**
  Andere wichtige Information kann unter "Kommentare" auf dem UE-Bogen angegeben werden; z.B. vorbestehende begünstigende Faktoren.

11 Dokumentation der Ergebnisse, Datenmanagement

11.1 Dokumentation und Korrektur der Daten

Alle während der Studie erhobenen Daten von allen in die Studie eingebrachten Patienten werden vom verantwortlichen Studienarzt oder einer von ihm beauftragten Person in die für diese Studie erstellten CRF eingetragen und mit Unterschrift und Stempel abgezeichnet. Die Daten müssen vollständig, eindeutig und plausibel sein. Fehlende Untersuchungen oder Daten müssen mit Angabe einer Begründung in den CRFs als solche gekennzeichnet werden.

Die Eintragung der während der Studie erhobenen Daten in die Patientenerhebungsbögen sollten mit schwarzem Kugelschreiber und gut leserlich vorgenommen werden. Evtl. notwendige Korrekturen sind vom Prüfarzt oder einer von ihm beauftragten Person vorzunehmen.

Die **Korrekturen** sollen entsprechend den GCP-Richtlinien durchgeführt werden, das heißt:
- die zu korrigierende Version wird so durchgestrichen, daß sie noch lesbar ist
- die gültige Version wird gut lesbar über oder neben die erste Version geschrieben

– die Korrektur (oder sonstige Eintragungen) werden vom Prüfarzt oder einer von ihm benannten Person mit Datum, Handzeichen und evtl. Grund der Änderung versehen.

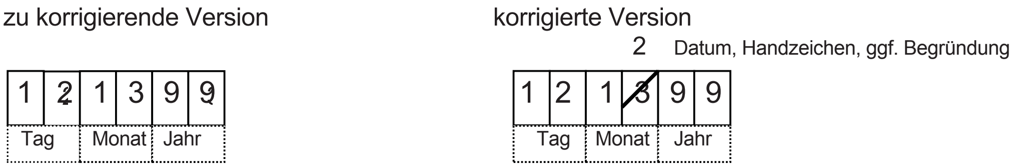

Falsche Eintragungen dürfen nicht mit Korrekturflüssigkeit überdeckt, ausgelöscht oder in anderer Weise unleserlich gemacht werden. Im Interesse einer einwandfreien Datenerhebung sind die Erhebungsbögen vollständig auszufüllen.
Korrekturen können aber auch anhand von Fehlerlisten durchgeführt werden, die der Monitor nach Durchsicht der Prüfbögen erstellt. Auf diesen Listen sind Prüfzentrum, Patientennummer, Patienteninitialen, Datum der Durchsicht, Seite, Fehler und korrekte Version genau aufgelistet. Die Richtigkeit aller Angaben, insbesondere der korrigierten Versionen, bestätigt der Prüfarzt oder ein von ihm benannter Vertreter durch seine Unterschrift auf der Fehlerliste. Eine vom Prüfarzt oder von einem von ihm benannten Vertreter unterzeichnete Fehlerliste stellt ein Original dar. Die Fehlerlisten müssen daher stets dem entsprechenden Prüfbogen beigeheftet werden, um die Vollständigkeit und die Validität der Daten zu gewährleisten.

11.2 Datenmanagement

Dateneingabe, Datenmanagement und statistische Analysen werden mit der Software ‚Statistical Analysis System' (SAS) durchgeführt. Die Eingabe erfolgt doppelt durch zwei verschiedene Personen. Hiervon ausgenommen sind Freitexte (außer Laboreinheiten): Diese werden nach einfacher Eingabe einer Sichtkontrolle unterzogen. Die aus dem Abgleich der Doppeleingaben resultierenden Fehlerkorrekturen werden dokumentiert. Datenübertragungen und –transformationen werden stichprobenartig überprüft oder validiert. Die Patientendaten werden auf Plausibilität überprüft. Rückfragen werden bei fehlenden, inkonsistenten oder unplausiblen Angaben ausgesandt. Änderungen der Datenbank werden nur von authorisierten Personen vorgenommen. Dabei wird ein Audittrail geführt, d.h. die Änderungen einschließlich Datum und durchführender Person werden festgehalten. Nach abschließender Bearbeitung der Rückfragen wird die Datenbank dem verantwortlichen Statistiker zur Auswertung zur Verfügung gestellt.

11.3 Kodierung der Daten

Die Kodierung von **Vorerkrankungen** und die Kodierung von **unerwünschten Ereignissen** erfolgt mit MedDRA (Medical Dictionary for Regulatory Activities).
Die Kodierung der **Begleitmedikationen** erfolgt nach dem ATC (Anatomisch-Therapeutisch-Chemischer-Code).

12 Qualtätssicherungssystem

Im Rahmen der klinischen Prüfung wird durch Monitoring und behördliche Überwachung die Qualitätskontrolle und Qualitätssicherung sichergestellt.

12.1 Qualitätskontrolle (Monitoring)

Zur Überprüfung der ordnungsgemäßen Dokumentation von Studiendaten wird der Prüfarzt dem Monitor Zugang zu den personenbezogenen Krankenakten gewähren. Dabei findet das Bundesdatenschutzgesetz volle Beachtung. Zur Datenüberprüfung sind auch Bevollmächtigte des Auftraggebers und die Behörden autorisiert.
Die Studie wird von einem speziell für diese Studie geschulten Monitor durch regelmäßige Besuche in den Prüfzentren und Telefonate mit den Prüfärzten überwacht. Die Prüfärzte gestatten dem Monitor

Besuche vor, während und nach Abschluss der Studie. Bei den Monitorbesuchen im Verlauf der klinischen Prüfung ist dem Monitor ein angemessener, ruhiger Arbeitsplatz zur Verfügung zu stellen.
Vom Prüfarzt muss für diese Besuche ausreichend Zeit eingeplant werden. Der Prüfarzt erlaubt dem Monitor Zugang zu den Originalunterlagen, welche die in die Prüfbögen eingetragenen Daten belegen. Der Monitor verpflichtet sich schriftlich, alle Daten, die unter die ärztliche Schweigepflicht fallen oder die Identität des Patienten offenlegen könnten, absolut vertraulich zu behandeln und nur der Verwendung zukommen zu lassen, mit der sich der Patient schriftlich einverstanden erklärt hat.

Nach Initiierung der Studie wird der Monitor mit den Prüfzentren kurzfristig und regelmäßig Kontakt halten, um sich über die Einhaltung des Prüfplans, die Übereinstimmung der in die Prüfbögen eingetragenen Daten mit den Originalunterlagen, die Datierung der Randomisation, die Unversehrtheit nicht gebrauchter Randomisationsumschläge, die Medikamenteninventarlisten, die Aufbewahrung der Studienmedikamente, die fortlaufende Führung der Patientenidentifikationsliste und das für die Originalunterlagen verwendete Ablagesystem zu informieren. Diese Besuche und Telefonate dienen auch dazu, den Fortgang der Studie zu überprüfen, Probleme frühzeitig zu erkennen und, falls vorhanden, zu beheben.

Der Prüfarzt sollte für den Monitorbesuch entsprechend Zeit einplanen. Falls eine andere Person mit einer entsprechenden medizinischen Ausbildung (z.B. Assistenzarzt) die Studie betreut und auch bei der Durchführung und Erhebung der Daten beteiligt ist, so ist auch deren Anwesenheit erforderlich.

12.2 Quelldatenvergleich (Source Data Verification [SDV])

Der Quelldatenvergleich dient dazu, die Eintragungen im CRF auf Richtigkeit und Vollständigkeit im Vergleich zu den Originaldaten zu überprüfen und somit die Qualität der Daten zu sichern und zu erhöhen. Alle Daten, die einer SDV unterworfen sind, müssen entweder in der Krankenakte eingetragen sein oder, falls es sich um Originalunterlagen handelt, der Krankenakte beigefügt sein. Zur Durchführung der SDV gewähren die Prüfärzte dem Monitor Einsicht in die Krankenakten.

Originaldaten im Sinne der EG-Note for Guidance (ICH-GCP 1996) sind:
- Krankenblätter
- Originalaufzeichnungen von automatisierten Geräten
- EKG
- Röntgenbilder
- Laboraufzeichnungen, etc.

12.2.1 Daten, die einer 100%igen SDV unterliegen

Für folgende Daten wird bei allen Patienten eine SDV durchgeführt:
- Initialen
- Geburtsdatum
- Geschlecht
- Unerwünschte Ereignisse (UEs)
- die wichtigsten Ein-/Ausschlusskriterien
- Einwilligungserklärung

12.2.2 Daten, die einer 20%igen SDV unterliegen

Alle weiteren nicht unter 12.2.1 aufgeführten Daten, zu denen Quelldaten nach 16.2.2 in der Krankenakte vorhanden sein müssen, werden zu ca. 20% mit der Krankenakte verglichen.

13 Biometrische Planung und Auswertung

13.1 Studiendesign

Es handelt sich um eine prospektive, randomisierte, kontrollierte, multizentrische Studie mit 4 Behandlungsarmen in einem 2x2-faktoriellen Design.

13.2 Zielkriterien

Primäres Zielkriterium ist die Veränderung der CAARS-O Skala (Summenscore) zwischen den Zeitpunkten T1 und T2 (siehe auch Kapitel 10.1)

Sekundäre Zielkriterien sind:

- Veränderung der CAARS-O Skala (Summenscore) zwischen den Zeitpunkten T1 und T3 bzw. T4.
- Veränderungen der CAARS-O Subskalen zwischen den Zeitpunkten T1 und T2, T3, T4.
- Veränderung der ADHS-DC zwischen den Zeitpunkten T1 und T2, T3, T4.
- Veränderung der CAARS-S Skala zwischen den Zeitpunkten T1 und T2, T3, T4.
- Veränderungen der CAARS-S Subskalen zwischen den Zeitpunkten T1 und T2, T3, T4.
- Veränderung des SCL-90-R zwischen den Zeitpunkten T1 und T2, T3, T4.
- Veränderung des BDI zwischen den Zeitpunkten T1 und T2, T3, T4.
- Veränderung des Q-LES-Q zwischen den Zeitpunkten T1 und T2, T3, T4.
- Veränderung des CGI zwischen den Zeitpunkten T1 und T2, T3, T4.
- Falls zutreffend: Veränderung des Nikotinkonsums zwischen den Zeitpunkten T1 und T2, T3, T4.
- Falls zutreffend: Veränderung des Nikotinkonsums zwischen den Zeitpunkten T1 und T2, T3, T4.
- Falls zutreffend: Veränderung des Koffeinkonsums zwischen den Zeitpunkten T1 und T2, T3, T4.
- Falls zutreffend: Veränderung des Ausmasses nicht stoffgebundener Süchte zwischen den Zeitpunkten T1 und T2, T3, T4.
- Fehltage am Arbeitsplatz/Ausbildungsplatz in den vier Behandlungsbedingungen im Vergleich.

13.3 Fallzahlkalkulation

Die Fallzahlkalkulation basiert auf dem primären Endpunkt „Veränderung der CAARS-O Skala zwischen den Zeitpunkten T1 und T2. In der primären Analyse wird die Basismessung des Scores als Kovariable berücksichtigt, daher erscheint eine Formulierung der zu erwartenden bzw. klinisch relevanten Effekte in Form von prozentualen Veränderungen angemessen.
Es handelt sich um ein 2x2-faktorielles Design, mit dem der Effekt der Psycho-therapie und der Effekt der Medikation untersucht werden soll. Dazu werden die beiden Gruppen, die Psychotherapie erhalten, verglichen mit den Patienten in den beiden Gruppen, die Clinical Management erhalten. Entsprechend werden die beiden Gruppen der Patienten, die die Prüfmedikation erhalten, verglichen mit den beiden Gruppen, die Placebo erhalten. Die erwarteten Effekte sind: 40% Reduktion in der CAARS Skala für Patienten, die Psychotherapie und Prüfmedikation erhalten; jeweils 30% Reduktion in den Patientengruppe, die Psychotherapie und Placebo bzw. Clinical Management und Prüfmedikation erhalten; 20% Reduktion in der Gruppe, die Clinical Management und Placebo erhält. Daraus ergibt sich, dass für die beiden Vergleiche (Medikation vs Placebo, Psychotherapie vs Clinical Management) jeweils mit einem Effekt von 10 Prozentpunkten zu rechnen ist, während der Vergleich der kombinierten Therapie mit Placebo / Clinical Management einen Effekt von 20 Prozentpunkten ergeben würde. Bei einer Berechnung in Form von Prozentpunkten und einer angenommen Standardabweichung von 30 Prozentpunkten ergeben sich hieraus Effektgrößen von 0.33 bzw. 0.67, wobei mit Effektgröße die standardisierte Differenz der prozentualen Reduktionen beim Vergleich von zwei Gruppen gemeint ist. Im Lichte der in der Übersichtsarbeit von Faraone et al. (2004) zusammengestellten Effektgrößen für Studien zum Vergleich von Methylphendiat vs Placebo bei ADHD bei Erwachsenen ist dies eine sehr vorsichtige Einschätzung, da sich in der Arbeit von Faraone eine über 6 Studien gepoolte Effektgröße von 0.9 ergab und sich die Effektgrößen für Fremd-Rating Skalen zwischen 0.3 und 2.3 bewegten.
Zum Nachweis einer Effektgröße von 0.33 werden pro Vergleich 175 Patienten pro Gruppe benötigt und damit 350 Patienten insgesamt, basierend auf einem zweiseitigen t-Test zum Niveau $\alpha=2.5\%$ bei einer Power von $1-\beta=80\%$. Mit der Adjustierung des Niveaus auf 2.5% wird berücksichtigt, dass in der primären Analyse zwei Vergleiche durchgeführt werden.
Da damit zu rechnen ist, dass von einem kleinen Teil der Patienten keine auswertbaren Daten vorliegen, so dass diese Patienten auch in der ITT-Analyse nicht berücksichtigt werden können, sollen 448 Patienten randomisiert werden. Es wird geschätzt, dass dazu 500 Patienten gescreent werden müssen. Um die dropout-Rate von Patienten zu berücksichtigen, die die Behandlung beginnen, aber nicht beenden, von denen aber für die ITT-Analyse verwertbare Daten vorliegen, wurde die nachzuweisende Effektgröße sehr konservativ angesetzt.

13.4 Definition der in die Analysen eingehenden Kollektive

Diese Studie wird nach dem "Intention-to-treat" Prinzip ausgewertet, d.h. dass die zu den Behandlungsarmen randomisierten Patienten als zum jeweiligen Arm zugehörig analysiert werden, unabhängig davon, ob sie die Therapie verweigert, abgebrochen haben oder andere Protokollverletzungen bekannt werden. Grundlage der primären Analyse ist daher das full analysis set (FAS).

Die "Per-Protocol" (PP)-Population ist eine Untergruppe des FAS und ist definiert als die Gruppe von Patienten, bei denen keine ernsthaften Protokollverletzungen vorliegen, die einen zuvor festgelegten Mindestumfang der Behandlung erhalten haben und bei denen die erforderlichen Untersuchungen zur Beurteilung der Zielkriterien an relevanten und zuvor festgelegten Zeitpunkten durchgeführt wurden. Die Auswertung der PP-Population erfolgt als Sensitivitätsanalyse.

Full analysis set (FAS): Das FAS schließt alle Patienten ein, bei denen Angaben zu dem Hauptzielparameter bei der Baselineerhebung T1 vorliegen.

Per protocol set (PPS): Patienten, die die Studie aufgrund mangelnder Compliance oder anderen präparatunspezifischen Gründen abbrechen, werden aus dem PPS ausgeschlossen. Falls bei einzelnen Patienten, die mindestens einmal das Prüfpräparat eingenommen haben oder an der Psychotherapie bzw. Clinical Management teilgenommen haben, Protokollverletzungen (z.B. Verstoß gegen die Ein-/Ausschlusskriterien, viele fehlende Daten) festgestellt werden, so wird von einem Studienbeteiligten, der blind für die Behandlungsbedingung ist, vor dem Beginn der Auswertung festgelegt, ob dieser Patient im PPS oder nur in dem FAS und der Verträglichkeitsanalyse berücksichtigt wird.
Patienten, die die Studie abbrechen wegen mangelnder Wirksamkeit oder unerwünschten Ereignissen, für die ein Zusammenhang mit der Studienmedikation nicht ausgeschlossen werden kann, werden in jedem Fall im PPS ausgewertet. Damit soll vermieden werden, dass die aus der PP-Analyse resultierende Effektschätzung zu optimistisch ist.

Safety analysis set (SAF): Jeder Patient gleich welcher Gruppe, der mindestens einmal eine Intervention erhalten hat (einmalige Teilnahme an Gruppentherapie, psychiatrischem Einzelgespräch, einmalige Medikationseinnahme von Verum oder Placebo), wird in die Verträglichkeitsanalyse miteinbezogen.

Vorgehen bei drop-outs oder/und fehlenden Werten:
Ist der Patient nicht mehr bereit, an der Studie teilzunehmen, so werden Werte des zuletzt wahrgenommenen Untersuchungstermins als Endwerte betrachtet und über LOCF in die Analyse einbezogen.
Sollten die Visiten t2, t3 oder t4 („Minivisiten“) die letzten wahrgenommenen Untersuchungstermine bei einem Patienten gewesen sein und keine Abschlussuntersuchung vorliegen, so wird der Wert der CAARS-S-L stellvertretend für den CAARS-O-L bei diesem Patienten eingesetzt.
Treten bei einem Score bei nicht mehr als 10% der Einzelitems fehlende Werte auf, so wird dieser Score in die Berechnungen mit einbezogen (Missings werden durch den Itemmittelwert ersetzt).

13.5 Zur Auswertung

13.5.1 Darstellung der Daten

Die Daten der Parameter zur Wirksamkeits- und Verträglichkeitsauswertung werden – sortiert nach Identifikationsnummer und Untersuchungstag – gelistet.
In Abhängigkeit vom Messniveau der einzelnen Variablen werden geeignete deskriptive Statistiken, z.B. Häufigkeiten (absolut und/oder prozentual), Anzahl der verfügbaren bzw. nicht verfügbaren Werte (non-missing bzw. missing data), Mittelwert, Standardabweichung, ggf. Median, unteres und oberes Quartil oder Interquartilsabstand, sowie p-Quantile, Minimum und Maximum oder Spannweite und ggf. Konfidenzintervalle berechnet und dargestellt. Diese Art der Darstellung wird auch für die während der Auswertung sinnvoll zu berechnenden Variablen (-kombinationen) verwendet.
Sollten gewisse Transformationen, z.B. zur Normalisierung oder zur Varianzstabilisierung der Daten, erforderlich werden, so werden sie ausführlich im statistischen Analyseplan dieser Studie beschrieben und begründet.
Die graphische Aufbereitung der wesentlichen Variablen erfolgt in Abhängigkeit vom Messniveau der Variablen mittels Stabdiagrammen, Histogrammen, Box-Whisker-Plots, Mittelwertsverläufen oder anderer gebräuchlicher Graphiken.
Der tatsächliche zeitliche Verlauf der Patientenrekrutierung wird ebenfalls dargestellt.

Alle Patienten, die die Studie vorzeitig abgebrochen haben, werden mit ihren Abbruchgründen tabellarisch dargestellt. Zusätzlich werden sie mit ihren demographischen Variablen, der Patientenidentifikation, den primären und sekundären Zielparametern und dem Abbruchgrund einzeln aufgelistet.

13.5.2 Analyse des primären Endpunktes

Die primäre Analyse erfolgt auf der Basis des oben definierten FAS. Um bei Patienten, die die Behandlung nicht protokollgerecht durchführen, dennoch möglichst aussagekräftige Daten zu erhalten, werden im Behandlungsverlauf Minivisiten durchgeführt, an denen die zentralen Wirksamkeitsparameter erfasst werden, so dass diese ggf. an Stelle möglicherweise fehlender Messungen zu den Zeitpunkten T2, T3, T4 eingesetzt werden können.
Der Vergleich der Behandlungen in Bezug auf die primäre Zielgröße (Veränderung des CAARS Summenscore von T1 nach T2) erfolgt innerhalb eines linearen Regressionsmodells, in dem die Behandlungen als 2 Faktoren mit jeweils 2 Kategorien (Psychotherapie ja/nein bzw. Medikation mit Methylphenidat ja/nein) formuliert werden.
Baselinewerte des CAARS Scores werden als Kovariable im Modell berücksichtigt. Dies steht in Einklang mit der EMEA Richtlinie "Points to Consider on Adjustment for Baseline Covariates" (CPMP/EWP/2863/99) und führt dazu, dass Ergebnisse des Behandlungsvergleichs unabhängig davon sind, ob Veränderungswerte oder Outcomes nach Beendigung der Therapie (T2) analysiert. Weiterhin wird im Modell für Studienzentren adjustiert. Die Festlegung weiterer relevanter Kovariablen erfolgt im Rahmen eines Statistischen Analyseplans (SAP) vor Beginn der Auswertung.
Im ersten Schritt der Analyse wird überprüft, ob eine Interaktion vorliegt, indem ein geeigneter Interaktionsterm im Modell formuliert und auf Signifikanz geprüft wird. Sollte der Interaktionsterm signifikant sein zum Niveau 10%, so wird er beibehalten, wenn das nicht der Fall ist, werden die Behandlungseffekte auf der Basis von 2 Haupteffekten ohne Wechselwirkung geschätzt.
Behandlungseffekte werden mit einem zweiseitigen 95%- Konfidenzintervall berichtet. Der Test auf Signifikanz der Behandlungseffekte wird jeweils zum zweiseitigen Niveau 2.5% durchgeführt, um zu berücksichtigen, dass 2 Haupthypothesen getestet werden.
Die weitere Untersuchung der Behandlungseffekte erfolgt hierarchisch, d.h. sie wird nur dann durchgeführt, wenn sich einer der beiden Haupteffekte als signifikant erwiesen hat. Daher ist hierfür keine weitere Adjustierung des Signifikanzniveaus erforderlich.

13.5.3 Weitere Analysen zur Wirksamkeit

Weitere Analysen zur Wirksamkeit umfassen:
- eine längerfristige Bewertung des Therapie-Effekte durch Erhebung des primären Endpunktes zu späteren Zeitpunkten (T3, T4);
- eine Bewertung der Therapie-Effekte an Hand der sekundären Wirksamkeitsparameter zu den Zeitpunkten T2, T3, T4.

Hierzu werden jeweils zur oben beschriebenen primären Analyse analoge Berechnungen durchgeführt, d.h. lineare Regressionsmodelle mit den Veränderungen seit T1 als Zielvariablen unter Einbeziehung der entsprechenden Baseline-Messungen, der Studienzentren, einer möglichen Interaktion sowie eventueller weiterer Kovariablen.

Vorgehen bei drop-outs oder/und fehlenden Werten:
Ist der Patient nicht mehr bereit, an der Studie teilzunehmen, so werden Werte des zuletzt wahrgenommenen Untersuchungstermins als Endwerte betrachtet und über LOCF in die Analyse einbezogen.
Sollten die Visiten t2, t3 oder t4 („Minivisiten") die letzten wahrgenommenen Untersuchungstermine bei einem Patienten gewesen sein und keine Abschlussuntersuchung vorliegen, so wird der Wert der CAARS-S-L stellvertretend für den CAARS-O-L bei diesem Patienten eingesetzt.
Treten bei einem Score bei nicht mehr als 10% der Einzelitems fehlende Werte auf, so wird dieser Score in die Berechnungen mit einbezogen (Missings werden durch den Itemmittelwert ersetzt).

13.5.4 Analyse der unerwünschten Ereignisse

Unerwünschte Ereignisse werden im Rahmen dieser Studie auf entsprechenden CRFs vom Prüfarzt erfasst mit den folgenden Parametern: Intensität, Zusammenhang mit dem Prüfpräparat, Beginn, Ende, schwerwiegend, (Gegen)-Maßnahmen, Ausgang. Alle unerwünschten Ereignisse werden getrennt nach Patient mit ihren weiteren Parametern (Intensität, Dauer etc.) sowie Beginn der Behandlung gelistet. Schwerwiegende unerwünschte Ereignisse werden zusätzlich gesondert gelistet.

Raten von (schwerwiegenden) unerwünschten Ereignissen werden getrennt nach Behandlungsgruppe mit 95%-Konfidenzintervallen berechnet. Entsprechend werden nach Behandlungsgruppen getrennt die Raten von (schwerwiegenden) unerwünschten Ereignissen dargestellt, für die ein Zusammenhang mit der Studienmedikation besteht bzw. nicht besteht.
Außerdem wird für das SAF Kollektiv zum einen die jeweilige Anzahl von Patienten mit unerwünschten Ereignissen berechnet und zum anderen die Anzahl der Patienten pro Organsystem nach MedDRA tabellarisch dargestellt. Diese Analyse wird für diejenigen unerwünschten Ereignisse, bei denen ein Kausalzusammenhang zumindest für möglich gehalten wird, wiederholt und wie oben beschrieben dargestellt.
Alle schwerwiegenden unerwünschten Ereignisse und Todesfälle werden individuell gelistet.

13.5.5 Auswertung der Laborparameter

Labordaten werden in den gemessenen Einheiten oder aus den Originaleinheiten konvertiert in SI Einheiten dargestellt. Werte außerhalb der lokalen Normbereiche werden kenntlich gemacht als unterhalb oder oberhalb des Normbereichs. Darüber hinaus werden Shifttabellen und zusammenfassende statistische Kennwerte errechnet.

13.5.6 Auswertung der Vitalparameter

Vitalparameter zu den einzelnen Zeitpunkten werden wie oben für die Laborparameter beschrieben analysiert. Die Veränderungen der Vitalparameter zum Ausgangswert werden als Mittelwert, Median, Range, Quartile getrennt nach Zeitpunkten und Behandlungsgruppen dargestellt.

13.5.7 Erstellung eines Statistischen Analyseplans

Vor Öffnung des Randomisierungscodes, Schließen der Datenbank und Durchführung der Analyse wird ein statistischer Analyseplan (SAP) erstellt, in dem die Details der Auswertung festgehalten werden.

Es wird zuvor ein „blind review“ durchgeführt, in dem die folgenden Punkte überprüft werden:

- Verteilung der primären und sekundären Zielgrößen
- Transformation erforderlich
- Streuung
- Extremwerte
- fehlende Werte
- Abbruchrate
- Protokollverletzungen
- Definition von FAS und PPS, Ausschluss von Patienten aus diesen Kollektiven.

13.6 Zwischenanalyse und Abbruchkriterien

Eine Zwischenanalyse ist vorgesehen für den Zeitpunkt, nach dem etwa die Hälfte (etwa 220) Patienten randomisiert und nach der wöchentlichen Studienbehandlung 3 Monate (T3) nachbeobachtet wurden. Die genaue Anzahl wird mit Rücksicht auf die Tatsache, dass gruppenweise randomisiert wird, in Abhängigkeit vom tatsächlichen Verlauf der Rekrutierung sinnvoll festgelegt. Gegenstand der Interimanalyse und des zugehörigen Zwischenberichts ist der Verlauf der Patientenaufnahme, die Compliance sowie Sicherheit und Verträglichkeit für das Patientenkollektiv und den Zeitraum wie oben beschrieben. Parameter für den Wirksamkeitsnachweis werden nicht ausgewertet. Nach Plausibilitätsprüfung der Daten und Auswertung wird der Zwischenbericht dem Unabhängigen Data Monitoring Committee (DMC, Kapitel 14.1) zur Stellungnahme vorgelegt.

14 Bedingungen für Protokolländerungen

14.1 Data Monitoring Committee (DMC)

Für die Überwachung der Studie wird ein unabhängiges Data Monitoring Committee (DMC) eingerichtet. Dieses gibt der Studienleitung nötigenfalls Empfehlungen zu Abbruch, Modifikation oder Fortsetzung der Studie. Aufgabe des DMC ist zu prüfen, ob die Durchführung der Studie weiterhin ethisch vertretbar ist, ob die Sicherheit der Patienten gewährleistet ist und ob die praktische Umsetzung der Fragestellung gerecht wird. Hierfür soll das DMC den Stand der Rekrutierung, die Einhaltung des Prüfplans und das Auftreten unerwünschter Ereignisse überwachen. Das DMC erhält den entsprechenden Bericht zur Zwischenanalyse. Die Zusammensetzung, Verantwortlichkeiten und Verfahrensweisen des DMC sowie die Kommunikation mit dem Vorstand des Netzwerkverbundes (Steering Committee) werden in einem DMC-Charter festgelegt.

14.2 Protokolländerungen

Protokolländerungen (Amendments) müssen von allen Personen unterzeichnet werden, die auch den Prüfplan unterzeichnet haben. Größere Änderungen verlangen zusätzlich die Miteinbeziehung des DMC. Alle beteiligten Studienzentren werden unmittelbar nach der Bewilligung eines Amendments informiert. Das Amendment ist jedem im Umlauf befindlichen Prüfplan hinzuzufügen.

Bezüglich des Inhaltes von Amendments unterscheidet man:

- Amendments, die die Gesundheitsinteressen des Patienten berühren könnten, erfordern eine erneute Beratung und ein Votum der zuständigen Ethikkommission und Bundesoberbehörde sowie eine erneute schriftliche Einwilligung der Patienten. Dies gilt auch für Patienten, die bereits in die klinische Prüfung aufgenommen wurden, sofern sie von dieser Änderung betroffen sind.

- Administrative oder technische Änderungen des Prüfplans, welche die Gesundheitsinteressen des Patienten nicht berühren, erfordern eine Prüfplanergänzung. In diesem Fall ist nur die Benachrichtigung der zuständigen Ethikkommission erforderlich.

Ein aus Sicherheitsgründen indiziertes Abweichen vom Protokoll kann für einen einzelnen Studienpatienten durch den Prüfarzt allein veranlasst werden (notwendige baldmögliche Meldung an den Sponsor).

15 Ethische und rechtliche Grundlagen

Die Prüfung wird in Übereinstimmung mit den gültigen gesetzlichen Anforderungen, insbesondere dem Deutschen Arzneimittelgesetz (AMG 1976 und Novellen), den GMP-, GLP- und ICH-GCP-Richtlinien sowie entsprechend den Prinzipien der Deklaration von Helsinki (1964 und Revisionen 1975, 1983, 1989, 1996, 2000) durchgeführt.

15.1 Aufklärung und Einwilligungserklärung des Patienten

Vor Aufnahme in die klinische Prüfung wird jeder Patient darüber informiert, dass die Teilnahme an der Studie freiwillig ist und dass er jederzeit ohne Angabe von Gründen und ohne dass ihm hierdurch ein Nachteil erwächst aus der Studie ausscheiden kann.

Der Patient wird vom behandelnden Arzt über die zu vergleichenden Behandlungsmethoden und die möglichen Risiken informiert. Gleichzeitig werden ihm Wesen, Bedeutung, Tragweite, erwartete Vorteile der Studie und Alternativbehandlungen erklärt. Die Aufklärung umfaßt auch die Unterrichtung des Patienten über den bestehenden Versicherungsschutz und die Obliegenheiten des Versicherten. Dem Patienten wird ausreichend Zeit und Gelegenheit gegeben, offene Fragen zu klären. Darüber hinaus wird

dem Patienten eine „Patientenaufklärung“ ausgehändigt, die alle wichtigen Informationen nochmals in schriftlicher Form enthält.

Die Einwilligung des Patienten muss **schriftlich vor Studienbeginn** erfolgen. Durch seine Unterschrift auf der Einwilligungserklärung erklärt der Patient seine freiwillige Teilnahme an der Studie und seine Absicht, den Erfordernissen der Studie und den Anweisungen des Prüfarztes während der Studie Folge zu leisten.

Die Einwilligungserklärung liegt in zweifacher Ausfertigung vor. Ein Exemplar verbleibt beim Prüfarzt, das andere Exemplar wird dem Patienten mitgegeben. Erst nach Unterzeichnung der Einwilligungserklärung ist diese gültig und der Patient kann, sofern er die Ein- und Ausschlußkriterien erfüllt, in die Studie eingeschlossen werden.

Mit der Einwilligungserklärung erklärt der Patient zugleich, dass er mit der im Rahmen der Studie erfolgenden Aufzeichnung von Krankheitsdaten und ihrer Weitergabe an die zuständige Überwachungsbehörde oder die zuständige Bundesoberbehörde einverstanden ist.

Der Prüfarzt bestätigt durch Unterschrift in dem jeweiligen Patientenprüfbogen verbindlich, dass ein individuelles Aufklärungssgespräch stattgefunden hat und von Seiten des Patienten eine unterschriebene Einwilligungserklärung vorliegt.

15.2 Behandlungsabbruch/ Studienabbruch

15.2.1 Vorzeitiger Studienabbruch durch den Patienten

Der Patient hat das Recht, jederzeit ohne Angabe von Gründen und ohne dass ihm hierdurch ein Nachteil erwächst bzw. Folgen für seine zukünftige Behandlung entstehen, die Zusage zur Teilnahme an der Studie zurückziehen und die Studie abzubrechen.

Der Zeitpunkt des Abbruchs, die bis dahin vorliegenden Ergebnisse und, falls bekannt, die Begründung für den Abbruch sind im CRF zu dokumentieren.

15.2.2 Therapieabbruch durch den Prüfarzt beim einzelnen Patienten

Der für die Studie verantwortliche, behandelnde Arzt hat das Recht, die Behandlung solcher Patienten abzubrechen, bei denen eines oder mehrere der folgende Vorkommnisse auftreten:

Mögliche und zu dokumentierende Gründe für den Abbruch der medikamentösen Behandlung, um die Sicherheit des Patienten zu gewährleisten:

- Die Einnahme der unter 4.5 genannten Medikamente; übrige Begleitmedikation (verschreibungspflichtig und nicht verschreibungspflichtig), die während der Studie eingenommen wurde, muss im CRF und in der Patientenakte dokumentiert werden (Indikation, Dosis, Applikationsform, Dauer der Einnahme).
- Auftreten nicht tolerabler UEs und/oder Laborwertveränderungen, Verschlechterung des Gesundheitszustandes des Patienten (nach Urteil des Prüfarztes).
- Verdacht auf Arzneimittelinteraktion(en).
- Neu aufgetretene Erkrankungen,
  - welche die Wirksamkeit der Studienbehandlung beeinflussen können,
  - für die die Einnahme des Prüfpräparats kontraindiziert sind (arterielle Hypertonie, Tachkardie, epileptischer Krampfanfall),
  - die eine Medikation bedingen, die als Begleitmedikation in der Studie nicht erlaubt ist.
- Positives Drogenscreening.
- Gewichtsabnahme mit BMI < 18.
- Eintritt einer Schwangerschaft.

Mögliche und zu dokumentierende Gründe für den Abbruch der psychotherapeutischen Gruppenbehandlung, um die Sicherheit des Patienten zu gewährleisten:

- Verschlechterung des Gesundheitszustandes des Patienten (nach Urteil des Prüfarztes), der in kausalem Zusammenhang zur Gruppenpsychotherapie zu sehen ist.

Mögliche und zu dokumentierende Gründe für den Abbruch der gesamten Studienbehandlung für den einzelnen Patienten:

- Widerruf der Patienteneinwilligung auch ohne Angabe von Gründen.
- Weitere Studienbeteiligung nach Beurteilung durch den Prüfarzt nicht akzeptabel/zumutbar.
- Nachträgliche Feststellung, dass Einschlusskriterien nicht erfüllt oder Ausschlusskriterien erfüllt sind, nach Entscheidung des Leiters der klinischen Prüfung.
- Arztwechsel, Ortswechsel des Patienten.
- Mangelnde Kooperation des Patienten.

Über den Behandlungsabbruch entscheidet der verantwortliche, behandelnde Arzt im Zentrum nach Rücksprache mit der Studienleitung.

Wichtig ist, dass so wenige Patienten wie möglich die Behandlung vorzeitig abbrechen und dass sie auch bei bereits aufgetretenen Protokollverstößen weiter behandelt werden, solange dies sinnvoll und möglich ist.
Dabei geht die Sicherheit des Patienten vor. Patienten, bei denen ein Behandlungsabbruch vorauszusehen ist oder bei denen die Behandlung abgebrochen wird, sollen möglichst dafür gewonnen werden, dennoch die eigentlich vorgesehenen Untersuchungstermine wahrzunehmen. Sollte dies nicht gelingen, so soll dem Patienten die Durchführung einer abschließenden Untersuchung vorgeschlagen werden.

Im Rahmen des vorzeitigen Abbruchs der Studienbehandlung und der Nachuntersuchung (4 Wochen später zur Erfassung von Unerwünschten Ereignisse, vgl. Kapitel 9.3.3 und 9.3.4) wird die Indikation zu weiteren Behandlungsmaßnahmen vom Prüfarzt geprüft. Bei vorhandener Indikation ist diese den Patienten mitzuteilen. Das Prüfzentrum unterbreitet bei Indikation ein Behandlungsabgebot oder ist bei der Organisation einer Weiterbehandlung behilflich. Modalitäten der Weiterbehandlung sind in der Krankengeschichte zu dokumentieren.

15.2.3 Abbruchkriterien für einzelne Teile der Studie oder die gesamte Studie:

- Bekannt werden von neuen Erkenntnissen und/oder Risiken, die eine erneute Nutzen-Risiko-Bewertung erforderlich machen und deren Ergebnis negativ ist (Entscheidung des Leiters der klinischen Prüfung, und des DMC, Empfehlung der zuständigen Ethikkommission, Empfehlung einer staatlichen Überwachungsinstitution)
- Organisatorische Gründe (z.B. mangelnde Patientenrekrutierung).

Beim Abbruch der gesamten Studie werden die Ethikkommission und die zuständigen Behörden über den Abbruchgrund informiert.

Die Prüfung kann vom Leiter der klinischen Prüfung jederzeit unter Abwägung des Nutzen-Risiko-Verhältnisses unterbrochen oder beendet werden.

Der Prüfarzt informiert den Sponsor und den Monitor 4 Wochen nach Studienende über weitere Ereignisse (UEs, SUEs, uUEs), die indirekt oder direkt mit der klinischen Prüfung im Zusammenhang stehen und/oder für die Beurteilung der Studienergebnisse relevant sein könnten.

15.2.4 Studienabbruch in einem Prüfzentrum

Bestehen seitens eines Prüfarztes ethische Bedenken bezüglich der Weiterführung der Studie in einem Zentrum, so muss dies unverzüglich dem Leiter der Klinischen Prüfung angezeigt werden. Der Leiter der Klinischen Prüfung ist berechtigt, die klinische Studie in einem Prüfzentrum vorzeitig zu beenden, wenn:

- die Patientenrekrutierung unzulänglich ist
- gravierende, nicht zu klärende Probleme mit der Qualität der erhobenen Daten auftreten
- unvorhersehbare Umstände im jeweiligen Prüfzentrum eingetreten sind, die eine Weiterführung der Klinischen Studie nicht zulassen.

15.2.5 Vorzeitige Beendigung der gesamten Studie

Mögliche Gründe für einen Abbruch der gesamten klinischen Prüfung:

- Bekannt werden von neuen Erkenntnissen und/oder Risiken, die eine erneute Nutzen-Risiko-Bewertung erforderlich machen und deren Ergebnis negativ ist (Entscheidung des Leiters der klinischen Prüfung, des wissenschaftlichen Studienleiters, Empfehlung der zuständigen Ethikkommission, Empfehlung einer staatlichen Überwachungsinstitution)
- Organisatorische Gründe (z.B. mangelnde Patientenrekrutierung)

Beim Abbruch der gesamten Studie werden die Ethikkommission und die zuständigen Behörden über den Abbruchgrund informiert.

Die Prüfung kann vom Leiter der klinischen Prüfung jederzeit unter Abwägung des Nutzen-Risiko-Verhältnisses unterbrochen oder beendet werden.

15.3 Datenschutz und Schweigepflicht

Im Verlauf der Studie erhobene Befunde werden auf elektronische Datenträger gespeichert und streng vertraulich behandelt.

Zum Schutz dieser Daten sind organisatorische Maßnahmen getroffen, die eine Weitergabe an unbefugte Dritte verhindern. So werden während der gesamten Dokumentations- und Auswertungsphase die Patienten lediglich anhand ihrer Initialen und der individuellen Patientennummer identifiziert, während der volle Name des Patienten nicht in Erscheinung tritt. Die einschlägigen Bestimmungen der länderspezifischen Datengesetzgebung sind vollumfänglich zu erfüllen.

Der Prüfarzt ist verantwortlich dafür, zu jedem Patienten ausreichende Informationen (Name, Geburtsdatum, Geschlecht, Patienteneinwilligungserklärung) aufzubewahren (um den Patienten identifizieren zu können). Diese Unterlagen müssen entsprechend den ICH-GCP-Richtlinien mindestens 15 Jahre lang aufbewahrt werden.

Die Auswertung der Studie erfolgt ausschließlich durch die Studienleitung und die beteiligten Partner (ZKS, Sponsor). Die verwendeten Scores, Prüfbögen und Unterlagen sind Eigentum der Studienleitung und dürfen ohne Genehmigung nicht anderweitig verwendet oder weitergegeben werden.

15.4 Einreichung bei Ethikkommissionen

Der Prüfplan und die Patientenaufklärung und -einwilligungserklärung werden zunächst bei der federführenden Ethikkommission und den beteiligten Ethikkommissionen zur Bewertung vorgelegt und es werden die .Voten eingeholt. Dabei folgt die Antragstellung bei den Ethikkommissionen den Bestimmungen der §§40 und 42 i.V.m. 42a und §7 Absatz 2 und 4 GCP-V.

Es sind folgende Unterlagen einzureichen:

- der Prüfplan (mit Amendments)
- die Patienteninformation und –einverständniserklärung
- Versicherungspolicen
- die Investigator´s Brochure
- der aktuelle Lebenslauf des Leiters der klinischen Prüfung
- Sicherheitsinformationen über das Prüfpräparat

15.5 Genehmigung

Vor Beginn der Prüfung werden die erforderlichen Unterlagen und Angaben auch bei der zuständigen Oberbehörde, BfArM, gemäß §§40 und 42 i.V.m. §7 Abs. 2 und 4 GCP-V- zur Genehmigung eingereicht.

Erst nach Vorliegen der positiven Voten der Ethikkommissionen und der Genehmigung der Bundesoberbehörde kann die Studie begonnen werden.

Der Ethikkommission werden umgehend alle Änderungen im Prüfplan, die nicht rein administrativer Art sind, sowie alle schwerwiegenden unerwünschten Ereignisse, die während der Studie auftreten und die Sicherheit der Studienteilnehmer oder die Durchführung der Studie beeinträchtigen könnten, mitgeteilt.

Eventuelle Empfehlungen und Hinweise der Ethikkommission werden gegebenenfalls in den Prüfplan eingearbeitet.
Beim Abbruch der gesamten Studie werden die Ethikkommission und die zuständigen Behörden über den Abbruch und Abbruchgrund informiert.

15.6 Allgemeine Anzeigepflicht

Vor Beginn der Studie werden die für die Prüfärzte zuständigen Landesbehörden gemäß § 67 (1) AMG über die geplante Prüfung informiert.

15.7 Patienten-/Probandenversicherung

Für alle an der Studie teilnehmenden Patienten wurde entsprechend § 40 Abs. 1 Nr. 8 und Abs. 3 AMG bei der

Ecclesia-Versicherung GmbH (Versicherungs-Nr. 70-005623465-5)
Klingenbergstrasse 4, D- 32758 Detmold
Telefon: 05231-603-377
FAX: 05231-603-197

eine Probandenversicherung (Minimum: 500.000,- € pro Patient) abgeschlossen. Der Prüfer hat die Patienten über die Versicherungsbedingungen schriftlich und mündlich zu informieren.
Um den Versicherungsschutz nicht zu gefährden, muss jede Gesundheitsschädigung, die als Folge der Teilnahme an der klinischen Prüfung eingetreten sein könnte, bei o.g. Adresse unverzüglich angezeigt werden. In Deutschland liegt die Meldepflicht beim Patienten.
Diesbezüglich hat der Versicherte allen zweckmäßigen Maßnahmen zuzustimmen, die der Aufklärung der Ursache und des Umfangs des eingetretenen Schadens und der Minderung dieses Schadens dienen.
Im Todesfall fällt die Meldepflicht auf den Rechtsnachfolger (Erbe). Der Todesfall ist innerhalb der von der Versicherung geforderten Zeit zu melden. Wird eine Gesundheitsschädigung im Rahmen der Studie nicht gemeldet, so erlischt der Versicherungsanspruch. Dies gilt auch für den Fall, dass sich der Patient nicht an die ärztlichen Anweisungen hält.
Während der Dauer dieser Prüfung darf sich der Patient einer anderen klinischen Behandlung - außer im Notfall - nur im Einvernehmen mit seinem Prüfarzt unterziehen.
Des weiteren besteht die Verpflichtung, alle unerwünschten Ereignisse und auch zusätzlich eingenommenen Medikamente dem behandelnden Arzt sofort mitzuteilen.
Der Prüfarzt hat den Patienten über die Vorgehensweise im Falle einer Schädigung durch die Therapieverfahren aufzuklären.
Der Prüfarzt steht dem Patienten jederzeit für die Klärung noch offener Fragen zur Verfügung. Der Patient hat das Recht, jederzeit in die beim Prüfarzt vorliegenden Versicherungsbedingungen Einsicht zu nehmen.

16 Studiendokumente und Archivierung Prüfarzt-Ordner

Beim Initiierungsvisit wird dem Prüfarzt zu Beginn der Studie ein Prüfarzt-Ordner übergeben. Dieser enthält alle essentiellen studienrelevanten Unterlagen. Der Prüfarzt-Ordner muß während der Monitorbesuche, für Audits der klinischen Qualitätssicherung und Behördeninspektionen zur Verfügung stehen. Nach Abschluss der Studie ist der Ordner gemäß den ICH-GCP-Richtlinien und den gültigen gesetzlichen Bestimmungen im Prüfzentrum aufzubewahren.

16.2 Dokumentation der Studiendaten

16.2.1 Patientenerhebungsbogen

Der Prüfarzt oder ein von ihm benannter Vertreter dokumentiert die Studiendaten zeitnah und kontinuierlich in einem studienspezifischen Patientenerhebungsbogen (case report form = CRF). Nach Möglichkeit sollte der Eintrag stets unmittelbar stattfinden.

Alle erhobenen Daten müssen korrekt, plausibel und vollständig vom Prüfarzt oder durch eine von ihm autorisierte Person ins CRF eingegeben werden.

16.2.2 Dokumentation der Studiendaten in der Krankenakte

Der Prüfarzt vermerkt von jedem Studienpatienten in der Krankenakte:

- Teilnahme an der Studie
- Beginn und Dauer der Studienbehandlung.
- Häufigkeit der Studienvisiten
- Dosierung der Prüfmedikation
- Art der Studienbehandlung (Gruppenpsychotherapie, psychiatrische Einzelgespräche)
- die relevanten Krankheitsdaten (körperlicher Untersuchungsbefund, Blutdruck, Puls, EKG, EEG, Körpergröße, Gewicht, Labor- und Urinbefunde bzw. Verweise auf Aufbewahrungs- oder Speichort der Originaldaten der Ableitungen)
- Begleitmedikation
- das Auftreten von unerwünschten Ereignissen und daraus gezogene Konsequenzen.

Quelldaten zu folgenden Daten dürfen im CRF eingetragen werden und müssen nicht im Krankenblatt festgehalten werden:

Eingangsdiagnostik und Messzeitpunke T1 bis T4

- Checkliste Ein- und Ausschlusskriterien
- SKID-I, -II
- Ergänzendes anamnestisches Interview (demographische Daten)
- Wender-Utah Rating Scale 25
- CAARS Selbsteinschätzung
- CAARS Fremdeinschätzung
- ADHS-Checkliste (ADHS-DC)
- Mehrfachwahl-Wortschatztest MWT
- BDI
- SCL-90-R
- Q-LES-Q
- CGI
- Fehltage am Arbeitsplatz
- Nikotinkonsum, Koffeinkonsum
- Ausmass nicht stoffgebundener Süchte

16.2.3 Patientenidentifikationsliste

Entsprechend den ICH-GCP-Empfehlungen muss der Prüfarzt eine Patientenidentifikationsliste führen, die eine eindeutige Zuordnung von Patientenname zu Patientennummer, Patienteninitialen, Geburtsdatum, Geschlecht sowie dem Einschluß in die Studie ermöglicht.

16.3 Archivierung

Der Prüfarzt-Ordner mit den essentiellen Studiendokumenten ist nach Beendigung der Studie im Prüfzentrum gemäß den ICH-GCP-Empfehlungen unter Berücksichtigung lokaler Gesetzgebung zu archivieren.

Die gesamte Studiendokumentation wird gemäß den gesetzlichen Bestimmungen und den ICH-GCP-Empfehlungen beim LKP Dr. med. Alexandra Philipsen, Oberärztin der Abteilung für Psychiatrie und Psychotherapie der Albert-Ludwigs-Universität Freiburg, Hauptstraße 5, 79104 Freiburg archiviert.

17 Vereinbarungen

17.1 Studienfinanzierung

Die Studie wird finanziert durch das Bundesministerium für Bildung und Forschung (Förderung von Forschungsverbünden zur Psychotherapie). Die Prüfmedikation (Methylphenidat und Placebo) wird unentgeltlich von der Firma Medice zur Verfügung gestellt.

17.2 Probandengelder

Aufwandsentschädigungen werden Patienten nicht grundsätzlich gewährt. Für bedürftige Patienten sind jedoch Mittel zur Fahrtkostenerstatttung vorhanden.(maximal 20 Euro pro Studientermin).

17.3 Berichte

Nach Abschluss der Auswertung durch den verantwortlichen Biometriker erstellt und unterzeichnet die Projektleitung gemeinsam mit dem Biometriker den integrierten medizinisch-statistischen **Abschlussbericht**.

Außer aus zwingenden rechtlichen Gründen wird niemand Dritten gegenüber ein Ergebnis der Studie mitgeteilt, bevor sich nicht alle beteiligten Seiten auf die Ergebnisse der Analyse und deren Interpretation verständigt haben.

17.4 Publikation

Das Recht zur **Publikation** liegt primär beim LKP und den Studienkoordinatoren an den beteiligten Kliniken. Alle im Zusammenhang mit der Studie erhobenen Daten werden bis zur Publikation durch den LKP und die weiteren Studienbeteiligten vertraulich behandelt. Die Publikation (entweder mündlich oder schriftlich) von zwischenzeitlich erhobenen Daten oder endgültigen Ergebnissen sollte nicht ohne vorherige Absprache zwischen dem Leiter der Klinischen Prüfung, den weiteren Prüfärzten bzw. Studienbeteiligten und dem Zentrum Klinische Studien erfolgen. Dies ist für einen möglichst erschöpfenden Informationsaustausch zwischen den oben genannten Parteien unabdingbar, um vor Publikation die Meinung aller Beteiligten gehört zu haben. Diese Aussage beinhaltet kein Vetorecht und kein Recht auf Zensur von einer der beteiligten Seiten. Ob eine Publikation erstellt wird oder nicht, hängt nicht von der Signifikanz der gefundenen Ergebnisse, sondern von der Relevanz der Resultate ab. Die Publikation sollte nach den Vorgaben des CONSORT-Statements erfolgen.

18 Studienbegleitende neurobiologische Untersuchungen

Parallel zum klinischen und pharmakologisch-psychotherapeutischen Kernthema der Studie werden neurobiologische Begleitforschungen durchgeführt, um so genetische oder hirnphysiologische Faktoren zu identifizieren, die als Marker der Erkrankung und des Therapieerfolg oder evt. sogar als Prädiktor des Therapieerfolgs geeignet erscheinen. Dabei handelt es sich zum einen um neurobiologische Untersuchungen zur strukturellen und neurochemischen Verfasstheit des Gehirns vor und nach der Therapiephase sowie im Langzeitverlauf sowie darüber hinaus um genetische Untersuchungen bei Therapieeinschluss (vgl. Flussdiagramm 9.4).

18.1 Projekt D1: In-Vivo-MR-Bildgebung

„Möglichkeiten der molekularen In-Vivo-MR-Bildgebung zur Identifikation von möglichen Endophänotypen der ADHS sowie von möglichen Prädiktoren des Therapieerfolgs (Molecular Imaging Might Predict Therapeutic Response in Adult Patients with ADHD. A Pilot Multimodal Neuroimaging Study)"

Projektleiter: PD Dr. L. Tebartz van Elst; Sprecher des Süddeutschen Brain Imaging Centers, Leiter der Sektion Experimentelle Neuropsychiatrie, Abteilung für Psychiatrie & Psychotherapie, Hauptstr. 5, Universitätsklinikum Freiburg, 79104 Freiburg; Tel: +49-761-270-6501; Fax: +49-761-270-6619; Email: tebartzvanelst@uniklinik-freiburg.de

18.1.1 Hintergrund und wissenschaftliche Fragestellung der MR-Studie

Volumenminderung in verschiedenen Gehirnarealen wurden in der Literatur bei Kindern und Erwachsenen mit einer ADHS beschrieben (v.a. rechtes Präfrontalhirn, Striatum und Cerebellum)[1]. Die Spezifität, Sensitivität und mögliche prädiktive Eigenschaften solcher zerebraler Auffälligkeiten wurden bislang aber noch nicht prospektiv untersucht.

Darüber hinaus wiesen einige Studien u.a. aus der eigenen Arbeitsgruppe auf neurochemische Auffälligkeiten verschiedener Hirnareale bei ADHS-Patienten hin. Solche Ergebnisse konnten dabei mit der Methode der MR Spektroskopie (MRS) gewonnen werden[2;3]. Da diese MR-Methode nicht mit einer Strahlenbelastung oder sonstigen gravierenden Nebenwirkungen behaftet ist, kann sie auch in prospektiven Reihenuntersuchungen eingesetzt werden. In neuesten MRS-Untersuchungen konnten darüber hinaus Signale der Neurotransmitter Glutamat und GABA quantifiziert werden, was die Möglichkeiten dieser Methodik zusätzlich ausweitet[4]. Damit werden Perspektiven eines neurobiologischen Monitorings therapeutischer Prozesse eröffnet, da mithilfe der MRS indirekte Hinweise auf Änderungen der Neurotransmitter in vivo gewonnen werden können.

Erste entsprechende Studien im Kontext der ADHS im Kindesalter zeigten, dass initial erhöhte Glutamatkonzentrationen im Striatum sich möglicherweise unter einer Therapie mit Methylphenidat normalisierten[5;6]. In Hinblick auf psychotherapeutische Prozesse liegen dagegen bislang noch keine Untersuchungen vor. Auch die Beziehungen zwischen strukturellen und neurochemischen Hirnauffälligkeiten sind bislang ungeklärt.

Vor diesem Hintergrund ist es das Ziel dieser Studie spezifische morphologische und neurochemische Endophänotypen der ADHS im Erwachsenenalter zu definieren. Ferner soll untersucht werden, ob diese Parameter auf eine Pharmakotherapie bzw. eine Psychotherapie ansprechen oder ob entsprechende Auffälligkeiten prädiktive Wertigkeit in Hinblick auf ein Ansprechen in den verschiedenen Therapiearmen haben.

Dabei ist das neurobiologische Projekt D1 in enger Anlehnung an das Hauptprojekt C1 organisiert. Die Patientenrekrutierung, -aufklärung und die Diagnostik werden komplett in Projekt C1 durchgeführt und sämtliche demografische und klinische sowie psychometrische Daten aus Projekt C1 übernommen. Im Rahmen des Projekts D1 werden die Teilstichproben aus C1 von den Standorten Freiburg und Mannheim zusätzlich longitudinal und prospektiv 3 Mal mithilfe der Magnetresonanztomographie untersucht.

Folgende Fragestellungen sollen dabei beantwortet werden:

Prädiktor und konfirmatorische Hypothesen:

1. Volumenminderungen im Bereich des Präfrontalhirns, des Striatums und des Zerebellums prädizieren ein schlechteres therapeutisches Ansprechen unabhängig vom Therapiemodus.
2. Patienten mit ADHD weisen verminderte striatale Glutamatsignale auf.

Explorative Hypothesen:

1. Eine erfolgreiche Psychotherapie führt zu einer Normalisierung zuvor erhöhter frontostriataler Glutamatkonzentrationen.
2. Es finden sich Unterschiede in den frontostriatalen neurochemischen Profilen zwischen erwachsenen Patienten mit einer ADHS und gesunden Kontrollprobanden.
3. Spezifische genetische Varianten (wie in Projekt D2 bestimmt) gehen mit spezifischen frontostriatalen Volumenminderungen und neurochemischen Auffälligkeiten einher (Definition eines Endophänotyps).

18.1.2 Studieneinschluss

Der Studieneinschluss erfolgt im Rahmen des Projekts C1. Diesbezüglich sei auf Kapitel 6 und 7 dieses Prüfplans verwiesen.

18.1.3 Ein- und Ausschlusskriterien

Die Ein- und Ausschlusskriterien entsprechen denen des Projekts C1. Diesbezüglich sei auf Kapitel 6 und 7 dieses Prüfplans verwiesen. Darüber hinaus gelten Endoprothesen aus Metall nicht entfernbare Metallteile im Körper wie z.B. im Rahmen von Herzschrittmachern sowie großflächige Tätowierungen mit Bleipartikel aus MR-technischen Gründen als zusätzliche Ausschlussfaktoren.

18.1.4 Untersuchungsprogramm

Die ersten 78 Patienten mit einer ADHS aus Projekt C1, die Ein- und Ausschlusskriterien erfüllen und der Untersuchung zustimmen, und 50 gruppengematchte Kontrollprobanden werden an den Standorten Freiburg und Mannheim identifiziert und eingeschlossen. Alle Patienten werden in einem 3 Tesla MR Scanner der Firma Siemens am Süddeutschen Brain Imaging Center in Freiburg untersucht. Dabei wird ein hochauflösender 3D-Datensatz mit folgenden Akquisitionsparametern aufgenommen: FOV 240 mm (FOV phase 100%), 1 slab; 160 slices; 30% slice oversampling, TR 2300; TI 1100; TE 3.68, flip angel 12°; width 140 Hz/px. Ferner wird eine neurochemische MR Untersuchung mit folgenden Parametern akquiriert: PRESS, TR 3000ms, TE 30ms, 96 averages und zwar im anterioren Cingulum sowie im Striatum. Wie der Übersichtstabelle auf Seite 8 zu entnehmen ist werden diese Untersuchungen bei Einschluss in die Studie (Baseline), nach Beendigung der intensiven 13-wöchigen Therapiephase sowie nach 1 Jahr durchgeführt.

18.1.5 Auswertung der Daten

Die Absolutquantifizierung der spektroskopischen Daten sowie die MR-morphometrischen Auswertungen der MR-Daten erfolgen am Süddeutschen Brain Imaging Center (SBIC) in Freiburg. Die Ergebnisse werden in ein Statistikprogramm (SPSS) mit den demografischen und klinischen Daten aus Projekt C1 zusammengeführt und am SBIC in Freiburg ausgewertet.
Was die sehr spezifischen konkreten Auswertealgorithmen anbelangt, sei auf frühere entsprechende Arbeiten aus der Arbeitsgruppe verwiesen [4].

18.1.6 Ethische Erwägungen

Die MR-Untersuchung wird etwa 45 Minuten dauern. Mit dieser Untersuchung sind bei Beachtung der Ausschlusskriterien nach aktuellem Stand des Wissens keine gravierenden Nebenwirkungen verbunden. Es findet keine Strahlenbelastung statt. Vorteilig ist für Patienten wie für Probanden, das sie eine kostenfreie qualitativ hochwertige Untersuchungen des Gehirns erhalten. Relevante aber bislang asymptomatische Erkrankungen wie z.B. Gefäßmissbildungen des Gehirn, die mit einem nennenswerten Blutungsrisiko einhergehen, könnten auf diese Art und Weise als asymptomatische Zufallsbefunde entdeckt und behandelt werden. Insofern haben Patienten wie gesunde Probanden sogar einen gesundheitlichen Nutzen von der Teilnahme an dieser Untersuchung.

18.2 Projekt D2: Genetik

„Untersuchung des Zusammenhangs zwischen Genotypen ADHS-relevanter Gene und der In-Vivo-MR-Bildgebung sowie dem Therapieverlauf (The impact of genetic variation on molecular imaging, habituation, and efficacy of cognitive behavioural therapy in adult ADHS)"

Projektleiter: Prof. Dr. med. Klaus-Peter Lesch, Klinik für Psychiatrie und Psychotherapie der Universität Würzburg,Füchsleinstraße 15, D- 97080 Würzburg, Tel: +49-931-201-77600, Fax: +49-931-201-77620, E-mail: kplesch@mail.uni-wuerzburg.de

Die Einwilligung nach Aufklärung der in die genetische Studie einbezogenen Patienten wird getrennt von der Einwilligung zur Teilnahme an der vorliegenden Klinischen Prüfung und der In-Vivo-MR-Bildgebung eingeholt werden. Es wird für D2 ein gesonderter Ethikantrag gestellt. Grundsätzlich besteht die Möglichkeit, dass Patienten die Teilnahme an der genetischen Studie ablehnen können, aber ihr Einverständnis für die Teilnahme an der klinischen Studie und In-Vivo-MR-Bildgebung geben.
Die einmalige zusätzliche Blutentnahme für das genetische Projekt wird für die zur Teilnahme bereiten Patienten im Rahmen der Eingangsdiagnostik der vorliegenden Klinischen Prüfung stattfinden.

18.2.1 Hintergrund und wissenschaftliche Fragestellung der Studie

Im Entstehungsgefüge der ADHS spielen genetische Faktoren eine wichtige Rolle. Die Erblichkeit ist mit berichteten Heritabilitätsindices um 0.8 hoch. Dennoch ist die ADHS als genetisch komplexe Störung zu verstehen, bei der zahlreiche Gene beteiligt sind. Genorte wurden in Kopplungs-Studien z.B. auf den Chromosomen 4q13.2, 5q33.3, 11q22, und 17p11 gefunden. Spezielle Varianten betreffen Proteine, die für die dopaminerge und serotonerge Signalübertragung im zentralen Nervensystem von Bedeutung sind. Eigene Studien gaben Hinweise auf eine Beteiligung des Catechol-O-Methyltransferase Gens bei Aufmerksamkeitsleistungen und des Tryptophanhydroxylase-2 Gens bei der über die Amygdala vermittelten emotionalen Dysregulation bei ADHS. Insgesamt ist jedoch das Wissen über die Beziehung molekulargenetischer, neurochemisch-struktureller sowie symptom- und verlaufsbezogener Aspekte der ADHS sehr begrenzt. Die vorliegende Studie soll in Verbindung mit den anderen Projekten des Verbunds die Rolle molekulargenetisch definierter Endophänotypen bezüglich Symptomatik und Prädiktion des Therapieerfolges sowie deren Assoziationen mit morphologisch-neurochemischen Besonderheiten untersuchen.

Hauptfragestellung:

1. Welche genetischen Variationen stellen Risikofaktoren für die Persistenz der ADHS im Erwachsenenalter dar?
2. Welchen Prädiktionswert haben spezifische Allele für das Ansprechen auf pharmakologische und psychotherapeutische Behandlung?
3. Sind spezifische morphologische oder neurochemische Besonderheiten (siehe Projekt D1, z.B. fronto-striatale Volumenminderungen, glutamaterge Transmission) mit spezifischen genetischen Varianten assoziiert?
4. Wie interagieren genetische Varianten untereinander und mit anderen Risikofaktoren (kritische Lebensereignisse, Persönlichkeitsdimensionen, Stressbewältigungsstrategien)?
5. Wie modulieren genetische Variationen basale Lernprozesse?

18.2.2 Studieneinschluss

Der Studieneinschluss erfolgt im Rahmen des Projekts C1. Diesbezüglich sei auf Kapitel 6 und 7 dieses Prüfplans verwiesen.

18.2.3 Ein- und Ausschlusskriterien

Die Ein- und Ausschlusskriterien entsprechen denen des Projekts C1. Diesbezüglich sei auf Kapitel 6 und 7 dieses Prüfplans verwiesen.

18.2.4 Arbeitsprogramm

Es werden die Patientenstichproben mit adultem ADHS aus C1 und D1 auf Assoziation verschiedener Endophenotypen, Parameter aus den bildgebenden Verfahren sowie der Therapieresponse mit den replizierten und funktionalen Polymorphismen der Gene für DRD4, DAT, 5HTT und MAOA überprüft. Darüberhinaus sollen Assoziationanalysen mit anderen etablierten Kandidatengenen der dopaminergen (z.B. COMT, Dopamin-beta-Hydroxylase), serotonergen (z.B. Serotonin-Rezeptoren 1A und 2C), noradrenergen (z.B. Noradrenalin-Transporter) und neurotrophen Systeme (z.B. BDNF) sowie im Rahmen des Projekts neu charakterisierten Kandidatengenen durchgeführt werden. Es wird beim Screening der Kandidatengene besonderes Augenmerk auf die Untersuchung von Polymorphismen mit nachgewiesener Funktionalität gelegt. Falls keine funktionalen Polymorphismen nachgewiesen sind, was meist für neu charakterisierte Kandidatengene zutrifft, werden geeignete SNP- und Repeat-basierende Haplotypen (Haplotyp-"Blöcke") definiert und für Assoziationsstudien herangezogen. Die Funktionalität dieser definierten Haplotypen wird dann mit entsprechenden Testverfahren überprüft.
Studiendauer: 3 Jahre.

19 Literaturangaben

19.1 Literatur C1

1. Adler LA, Spencer TJ, Milton DR. Long-term, open-label study of the safety and efficacy of atomoxetine in adults with attention-deficit/hyperactivity disorder: an interim analysis. J Clin Psychiatry 2005;66:294-9.
2. Beck AT, Ward CH, Mendelson M, Mock H, Erbaugh J: An inventory for measuring depression. Arch Gen Psychiatry 1961;4:561-571.
3. Biederman J. Impact of comorbidity in adults with attention-deficit/hyperactivity disorder. J Clin Psychiatry 2004;65:3-7.
4. Bobb AJ, Castellanos FX, Addington AM et al. Molecular genetic studies of ADHD: 1991 to 2004. Am J Med Genet B Neuropsychiatr Genet 2005;132:109-25.
5. Brown TE (Editor). Attention-Deficit Disorders and Comorbidities in Children, Adolescents, and Adults. Washington DC: American Psychiatric Press; 2000.
6. Cohen-Zion M, Ancoli-Israel S. Sleep in children with attention-deficit hyperactivity disorder (ADHD): a review of naturalistic and stimulant intervention studies. Sleep Med Rev 2004;8:379-402.
7. Derogatis LR. SCL-90-R: Administration, Scoring and Procedures Manual II. 1977. Towson, MD, Clinical Psychometric Research.
8. Ebert D, Berger M, Hesslinger B. ADHS als Risikofaktor für Dissozialität und Persönlichkeitsstörungen. MMW Fortschr Med 2002; 47:1005-1007.
9. Ebert D, Krause J, Roth-Sackenheim C. ADHS im Erwachsenenalter - Leitlinien auf der Basis eines Expertenkonsensus mit Unterstützung der DGPPN. Nervenarzt 2003;74:939-946.
10. Elia J: Stimulants and antidepressant pharmacokinetics in hyperactive children. Psychopharm Bull 1991, 27: 411-415
11. Endicott J, Nee J, Harrison W, Blumenthal R: Quality of Life Enjoyment and Satisfaction Questionnaire: a new measure. Psychopharmacol Bull. 1993;29(2):321-6.
12. Faraone SV, Biederman J, Mick E: The age-dependent decline of attention deficit hyperactivity disorder: a meta-analysis of follow-up studies. *Psychological Medicine* 2006; **36**:159-165.
13. Faraone SV, Spencer T, Aleardi M. Meta-analysis of the efficacy of methylphenidate for treating adult attention-deficit/hyperactivity disorder. J Clin Psychopharmacol. 2004;24:24-9.
14. First, M. B. Spitzer R. L. Gibbon M. et. al., Structured Clinical Interview for DSM-IV Axis I Disorders (SCID-I)-Clinical Version. Washington D.C.: American Psychiatric Press; 1997.
15. Greenhill L: Attention-deficit hyperactivity disorder: the stimulants. Child Adolesc Psychiat Clin North America 1995, 4: 123-168.
16. Gualtieri CT, Hicks RE, Patrick K, Schroeder SR, Breese GR: Clinical correlates of methylphenidate blood levels. Therap Drug Monitor 1984, 6: 279-392
17. Guy W. ECDEU Assessment Manual for Psychopharmacology, 1976
18. Hesslinger B, Mochan F, Tebartz van Elst et al. Attention deficit hyperactivity disorder in adults – early versus late onset in a retrospective study. Psych Res 2003;119:217-223.
19. Hesslinger B, Philipsen A, Richter H. Psychotherapie der ADHS im Erwachsenenalter - Ein Arbeitsbuch. Göttingen: Hogrefe; 2004.
20. Hesslinger B, Tebartz van Elst L, Nyberg E et al. Psychotherapy of attention deficit hyperactivity disorder in adults - A pilot study using a structured skills training program. Eur Arch Psychiatry Clin Neurosci 2002;252:177-184.
21. Kessler RC, Adler L, Barkley R et al. The prevalence and correlates of adult ADHD in the United States: results from the National Comorbidity Survey Replication. Am J Psychiatry 2006; 163(4):716-23.
22. Krause J & Krause KH. ADHS im Erwachsenenalter, 2. Auflage. Stuttgart: Schattauer; 2005.
23. Linehan, Marsha M., Cognitive-Behavioral Treatment of Borderline Personality Disorder. New York: The Guildford Press; 1993.
24. Michelson D, Adler L, Spencer T et al: Atomoxetin in Adults with ADHD: Two Randomized, Placebo-Controlled Studies. Biol Psychiatry (2003) 53: 112-120
25. Paterson R, Douglas C, Hallmayer J, Hagan M, Krupenia Z : A randomised, double-blind, placebo-controlled trial of dexamphetamine in adults with attention deficit hyperactivity disorder. Australian New Zealand J psychiatry (1999) 33: 494-502

26. Philipsen A, Feige B, Hesslinger B. Sleep in Adults with Attention Deficit/Hyperactivity Disorder: A Controlled Polysomnographic Study including Spectral Analysis of the Sleep EEG. SLEEP 2005;28:
27. Rosler M, Retz W, Retz-Junginger P et al: Prevalence of attention deficit-/hyperactivity disorder (ADHD) and comorbid disorders in young male prison inmates. Eur Arch Psychiatry Clin Neurosci. 2004 Dec;254(6):365-71.
28. Safren SA, Otto MW, Sprich S et al. Cognitive-behavioral therapy for ADHD in medication-treated adults with continued symptoms. Behav Res Ther. 2005 Jul;43(7):831-42.
29. Spencer T, Biederman J, Wilens T, Faraone S, Prince J, Gerard K, Doyle R, Parekh A, Kagan J, Bearman SK: Efficacy of a mixed amphetamine salts compound in adults with attention-deficit hyperactivity disorder. Arch Gen Psychiat 2001, 58: 775-782
30. Spencer T, Biederman J, Wilens T, Prince J, Hatch M, Jones J, Harding M, Faraone S, Seidman L: Effectiveness and tolerability of tomoxetine in adults with attention deficit hyperactivity disorder. Am J Psychiat 1998, 155: 693-695
31. Spiss C, Danesh R, Study on the relative Bioavailability of One 20 mg Methylphenidat Hydrochloride Modified Release Capsule Formulation After Single Dose Oral Administration Following a High Calory Breakfast and One 10 mg Methylphenidate Hydrochloride Immediate Release Tablet Formulation After b.i.d. Oral Administration on Empty Stomach in 12 Healthy Volunteers, not published
32. Steinhausen HC. Attention-deficit hyperactivity disorder in a life perspective. Acta Psychiatr Scand 2003;107:321-322
33. Stevenson CS, Whitmont S, Bornholt L et al. A cognitive remediation programme for adults with Attention Deficit Hyperactivity Disorder. Aust N Z J Psychiatry 2002;36: 610-616.
34. Taylor FB, Russo J: Efficacy of Modafinil compared to dextroamphetamine for the treatment of attention deficit hyperactivity disorder in adults. J Child Adolesc Psychopharmacol 2000, 10: 311-320
35. Ward MF, Wender PH, Reimherr FW. The Wender Utah Rating Scale: an aid in the retrospective diagnosis of childhood attention deficit hyperactivity d. Am J Psychiatry 1993;150:885-890
36. Ward, M. F., Wender, P. H., and Reimherr, F. W. The Wender Utah Rating Scale: an Aid in the Retrospective Diagnosis of Childhood Attention Deficit Hyperactivity Disorder. Am.J.Psychiatry 1993;150(6):885-90.
37. Wender PH, Reimherr FW, Wood D, Ward M : A controlled study of methylphenidate in the treatment of attention deficit disorder, residual type, in adults. Am J Psychiat 1985, 142: 547-552
38. Wender PH. Die Aufmerksamkeitsdefizit-/Aktivitätsstörung im Erwachsenenalter. Psycho 2000;26:190-198.
39. Wilens TE, Biederman J, Prince J, Spencer TJ, Faraone SV, Warburton R, Schleifer D, Harding M, Linehan D, Geller D: Six week, double-blind, placebo-controlled study of desipramine for adult attention deficit hyperactivity disorder. Am J Psychiatry 1996, 153: 1147-1153
40. Wilens TE, Biederman J, Spencer TJ, Bostic J, Prince J, Monuteaux MC, Soriano J, Fine C, Abrams A, Rater M, Polisner D: A pilot controlled clinical trial of ABT-418, a cholinergic agonist, in the treatment of adults with attention deficit hyperactivity disorder. Am J Psychiat 1999b, 156: 1931-1937
41. Wilens TE, Biederman J, Spencer TJ, Frazier J, Prince J, Bostic J, Rater M, Soriano J, Hatch M, Sienna M, Millstein RB, Abrantes A: Controlled trial of high doses pemoline for adults with attention-deficit hyperactivity disorder. J Clin Psychopharmacol 1999a 19: 257-264
42. Wilens TE, Biederman J, Spencer TJ. Attention Deficit/Hyperactivity Disorder Across The Lifespan. Annu Rev Med 2002;53:113-131.
43. Wilens TE, Biederman J. The stimulants. Ped Clin North America 1992, 15: 191-222
44. Wilens TE, Faraone SV, Biederman J. Does stimulant therapy of attention deficit/hyperactivity disorder beget later substance abuse? A meta-analytic review of the literature. Pediatrics 2003;111:179-185.
45. Wilens TE, Haight BR, Horrigan JP. Bupropion XL in adults with attention-deficit/hyperactivity disorder: a randomized, placebo-controlled study. Biol Psychiatry. 2005;57:793-801.
46. Wilens TE, Hammerness PG, Biederman J et al. Blood pressure changes associated with medication treatment of adults with attention-deficit/hyperactivity disorder..J Clin Psychiatry,. 66(2):253-9, 2005.
47. Winsberg BG, Kupietz SS, Sverd J, Hungund BL, Young NL: Methylphenidate oral dose plasma concentration and behavioural response in children. Psychopharmacology 1982, 76:329-332.

19.2 Literatur D1

1. Faraone SV, Biederman J, Spencer T, Seidman LJ, Mick E, Doyle AE. Attention-Deficit/Hyperactivity Disorder in Adults: An Overview. Biol Psychiatry 2000; 48:9-20. Biological Psychiatry 2000;48:9-20.
2. Hesslinger B, Thiel T, Tebartz vE, Hennig J, Ebert D. Attention-deficit disorder in adults with or without hyperactivity: where is the difference? A study in humans using short echo (1)H-magnetic resonance spectroscopy. Neuroscience Letters 2001;304:117-9.
3. MacMaster FP, Carrey N, Sparkes S, Kusumakar V. Proton spectroscopy in medication-free pediatric attention-deficit/hyperactivity disorder. Biol.Psychiatry 2003;53:184-7.
4. Tebartz van Elst, L, Valerius, G., Büchert, M., Thiel, T., Hennig, J., Ebert, D., and Olbrich.D. Increased Prefrontal and Hippocampal Glutamate Concentration in Schizophrenia.Evidence from a Magnetic Resonance Spectroscopy Study. Biological Psychiatry . 2005. (In Press)
5. Carrey N, MacMaster FP, Sparkes SJ, Khan SC, Kusumakar V. Glutamatergic changes with treatment in attention deficit hyperactivity disorder: a preliminary case series. J.Child Adolesc.Psychopharmacol. 2002;12:331-6.
6. Yeo RA, Hill DE, Campbell RA, Vigil J, Petropoulos H, Hart B, Zamora L, Brooks WM. Proton magnetic resonance spectroscopy investigation of the right frontal lobe in children with attention-deficit/hyperactivity disorder. J.Am.Acad.Child Adolesc.Psychiatry 2003;42:303-10.
7. Tebartz van Elst L, Woermann FG, Lemieux L, Thompson PJ, Trimble MR. Affective aggression in patients with temporal lobe epilepsy: a quantitative MRI study of the amygdala. Brain 2000;123:234-43.
8. Tebartz van Elst LT, Thiel T, Hesslinger B, Lieb K, Bohus M, Hennig J, Ebert D. Subtle prefrontal neuropathology in a pilot magnetic resonance spectroscopy study in patients with borderline personality disorder. Journal of Neuropsychiatry & Clinical Neurosciences 2001;13:511-4.
9. Tebartz van Elst, L, Ludaescher, P., Thiel, T., Büchert, M., Hesslinger, B., Lieb, K., Bohus, M., Rusch, N., Hennig, J., and Ebert, D. Association of Disturbed Amygdala Energy Metabolism and Volume Loss in Patients with Borderline Personality Disorder. Biol Psychiat (In Review)
10. Tebartz vE, Hesslinger B, Thiel T, Geiger E, Haegele K, Lemieux L, Lieb K, Bohus M, Hennig J, Ebert D. Frontolimbic brain abnormalities in patients with borderline personality disorder: a volumetric magnetic resonance imaging study. Biological Psychiatry 2003;54:163-71.
11. Rusch N, Tebartz van Elst L, Ludaescher P, Wilke M, Huppertz HJ, Thiel T, Schmahl C, Bohus M, Lieb K, Hesslinger B, Hennig J, Ebert D. A voxel-based morphometric MRI study in female patients with borderline personality disorder. Neuroimage. 2003;20:385-92.

20 Genehmigung des Prüfplans

Leiter der Klinischen Prüfung

Unterschrift Ort / Datum

Studienkoordinator

Unterschrift Ort / Datum

Statistiker

Unterschrift Ort / Datum

Sponsor

Unterschrift Ort / Datum

Original der Seite: Trial Master File

21 Anhang

21.1 Liste der teilnehmenden Prüfzentren

C1 und D2

1. Universitätsklinik der Albert-Ludwigs Universität Freiburg, Abteilung für Psychiatrie und Psychotherapie, Hauptstraße 5, D- 79104 Freiburg (Ansprechpartner: LKP).
2. Klinik für Psychiatrie und Psychotherapie des Zentralinstituts für Seelische Gesundheit, J5, D-68072 Mannheim (Ansprechpartnerin: Dr. Esther Sobanski).
3. Institut für gerichtliche Psychologie und Psychiatrie der Universität des Saarlandes, D-66421 Homburg/Saar (Ansprechpartner: Prof. Michael Rösler).
4. Klinik für Psychiatrie und Psychotherapie Charité Campus Benjamin Franklin, Eschenallee 3, D-14050 Berlin (Ansprechpartner: Dr. Michael Colla).
5. Klinik für Psychiatrie und Psychotherapie der Julius-Maximilians Universität Würzburg, Füchsleinstrasse 15, D-97070 Würzburg (Ansprechpartner: Dr. Christian Jacob).

D1

1. Universitätsklinik der Albert-Ludwigs Universität Freiburg, Abteilung für Psychiatrie und Psychotherapie, Hauptstraße 5, D-79104 Freiburg (Ansprechpartner: Dr. Ludger Tebartz van Elst).
2. Klinik für Psychiatrie und Psychotherapie des Zentralinstituts für Seelische Gesundheit, J5, D-68072 Mannheim (Ansprechpartnerin: Dr. Esther Sobanski).

D2

Labor für molekulare und klinische Psychobiologie, Klinik für Psychiatrie und Psychotherapie der Universität Würzburg, Füchsleinstraße 15, D-97080 Würzburg, Telefon: +49-931-201-77600, Fax: +49-931-201-77620 (Ansprechpartner: Prof. Klaus-Peter Lesch).

21.2 Verpflichtung des Prüfers zur Prüfplaneinhaltung am Zentrum

Der Unterzeichnende verpflichtet sich mit seiner Unterschrift, dass er die Studie nach diesem Prüfplan unter Einhaltung der Deklaration von Helsinki, der ICH-GCP-Empfehlungen und unter Beachtung des Deutschen Arzneimittelgesetzes durchführen wird. Zusammen mit den ICH-GCP-Empfehlungen ist dieses Prüfplan Bestandteil des Vertrages zwischen Auftraggeber und Prüfarzt.

**Titel:**	**Vergleich einer strukturierten störungsspezifischen Gruppenpsychotherapie plus Placebo oder Methylphenidat versus einer psychiatrischen Beratung plus Placebo oder Methylphenidat bei ADHS im Erwachsenenalter – eine erste randomisierte Multizenter-Studie**
**EudraCT No.:**	2006-000222-31
Projekt Nr.:	070170
***Leiter der klinischen Prüfung** (gemäß AMG):*	Dr. med. Alexandra Philipsen, Universitätsklinikum der Albert-Ludwigs-Universität Freiburg, Abteilung für Psychiatrie und Psychotherapie, Hauptstrasse 5, D- 79104 Freiburg

Prüfzentrum:

Hauptprüfer im Zentrum:

Ich bestätige, dass ich den Studienprüfplan gelesen habe und verpflichte mich hiermit alle Abläufe und Bestimmungen, wie in den entsprechenden Kapiteln der klinischen, ethischen- und allgemeinen Abschnitten spezifiziert, zu befolgen.

Ich bestätige, dass ich und meine Mitarbeiter die hiesige Gesetzgebung (in Deutschland das Deutsche Arzneimittelrecht mit den jeweils aktuell gültigen Novellen) beachten. Ich bestätige ferner, dass die klinische Prüfung unter Einhaltung der Deklaration von Helsinki und der ICH-GCP-Gesetzgebung durchgeführt wird.

Ich bestätige, dass ich alle vertraulichen Informationen in diesem Dokument, außer für die Durchführung oder die Auswertung der klinischen Prüfung, ohne die schriftliche vorherige Zustimmung des Sponsor, nicht verwenden oder weitergeben werde.

Unter meiner Verantwortung überlasse ich meinen Mitarbeitern Kopien dieses Prüfplans und eventuellen Aktualisierungen sowie Zugang zu allen Informationen in Bezug auf die Durchführung dieser klinischen Prüfung. Ich werde mit meinen Mitarbeitern dieses Dokument ausführlich besprechen und sicherstellen, dass sie umfassend über das Prüfpräparat und die Studiendurchführung informiert sind.

Ferner verpflichte ich mich, dass ich vor der Behördengenehmigung und zustimmenden Bewertung der zuständigen Ethik-Kommission nicht mit dem Einschluss von Patienten beginnen werde.

____
Name des Untersuchers

____
Datum, Unterschrift des Untersuchers

Klinik/Abteilungsstempel

22 Maßgebliche Richtlinien und Gesetze

Declaration of Helsinki	http://ohsr.od.nih.gov/helsinki.php3
ICH-GCP-Guidelines	*http://www.ich.org*
Auszug aus dem AMG	*AMG § 40 -42a*
