# Supplementary material for: Long-term Effects of Multimodal Treatment on Adult Attention-Deficit/Hyperactivity Disorder Symptoms: Follow-up Analysis of the COMPAS Trial
Source: JAMA Netw Open. 2019 May 31;2(5):e194980. doi: 10.1001/jamanetworkopen.2019.4980 (PMC6547099; doi:10.1001/jamanetworkopen.2019.4980)
Supplement: Supplement 2. — eTable 1. 2 × 2 Factorial Study Design eTable 2. Treatments After T4 by Former Randomized Treatment Groups eTable 3. Daily Dosage MPH (mg) at T5 by Former Randomized Treatment Groups eTable 4. Daily Dosage MPH (mg/kg) at T5 by Former Randomized Treatment Groups eTable 5. Primary and Secondary Outcomes at 2.5 Years (T5) eTable 6. Stability T5-T4 eTable 7. Self-Rated CAARS ADHD Index and BDI Total Score eTable 8. Major Secondary Outcomes in the Full Analysis Set at Follow-Up (T5) 2.5 Years After Baseline (T1) [file jamanetwopen-2-e194980-s002.pdf]

Supplementary Online Content

Lam AP, Matthies S, Graf E, et al; Comparison of Methylphenidate and Psychotherapy in Adult ADHD Study (COMPAS) Consortium. Long-term effects of multimodal treatment on adult attention-deficit/hyperactivity disorder symptoms: follow-up analysis of the COMPAS trial. *JAMA Netw Open*. 2019;2(5):e194980. doi:10.1001/jamanetworkopen.2019.4980

eTable 1. 2 × 2 Factorial Study Design

eTable 2. Treatments After T4 by Former Randomized Treatment Groups

eTable 3. Daily Dosage MPH (mg) at T5 by Former Randomized Treatment Groups

eTable 4. Daily Dosage MPH (mg/kg) at T5 by Former Randomized Treatment Groups

eTable 5. Primary and Secondary Outcomes at 2.5 Years (T5)

eTable 6. Stability T5-T4

eTable 7. Self-Rated CAARS ADHD Index and BDI Total Score

eTable 8. Major Secondary Outcomes in the Full Analysis Set at Follow-Up (T5) 2.5 Years After Baseline (T1)

This supplementary material has been provided by the authors to give readers additional information about their work.

© 2019 Lam AP et al. *JAMA Network Open.*

eTable 1 2 × 2 Factorial Study Design.

	GPT	CM	Total
**MPH**	107	110	217
**Plac**	109	107	216
**Total**	216	217	433

Data are numbers of participants. GPT = group psychotherapy. MPH = Methylphenidate. CM = clinical management. Plac = Placebo.

© 2019 Lam AP et al. *JAMA Network Open.*

eTable 2 Treatments After T4 by Former Randomized Treatment Groups

**Treatment at T5**	**GPT + MPH** No. [%]	**GPT + Plac** No. [%]	**CM + MPH** No. [%]	**CM + Plac** No. [%]
MPH between T4-T5, but not at T5	5/64 [7.8]	9/66 [14]	5/68 [7.4]	4/53 [7.6]
MPH at T5	16/64 [28.1]	22/66 [33.3]	25/68 [36.8]	15/53 [28.3]
Medication other than MPH at T5	4/64 [6.3]	15/66 [23]	15/68 [22]	7/53 [13])
Antidepressants at T5	4/64 [6.3]	14/66 [21.2]	16/68 [20.6]	5/53 [9.4]
Subgroup Venlafaxine/Bupropion	1 [1.6]	4 [6.1]	5 [7.1]	0 [0.0]
Antidepressants + MPH at T5	1/64 [1.6]	7/66 [11]	2/68 [2.9]	0/53 [0]
Non-pharmacologic treatment(s) at T5	18/64 [28]	16/66 [24]	20/68 [29]	18/53 [34]
Behavioural therapy once or twice/week	4 [6.3]	2 [3.0]	3 [4.4]	3 [5.7]
Behavioural therapy every 2 weeks	0 [0.0]	2 [3.0]	1 [1.5]	2 [3.8]
Behavioural therapy other frequencies (less)	2 [3.1]	5 [7.6]	2 [2.9]	2 [3.8]
Group therapy	1 [1.6]	0 [0.0]	0 [0.0]	0 [0.0]
Family therapy	0 [0.0]	1 [1.5]	2 [2.9]	0 [0.0]

GPT = group psychotherapy. MPH = Methylphenidate. CM = clinical management. Plac = Placebo.

© 2019 Lam AP et al. *JAMA Network Open.*

eTable 3 Daily Dosage MPH (mg) at T5 by Former Randomized Treatment Groups

	Daily dosage MPH [mg] at T5						
	**Total**	**Missing**	**Total valid**	**Mean**	**SD**	**Minimum**	**Maximum**
GPT + MPH	18	3	15	35.62	21.32	11.4	80.0
GPT + Plac	22	2	20	38.05	17.02	10.0	70.0
CM + MPH	25	4	21	38.03	34.90	8.6	160.0
CM + Plac	15	4	11	28.91	18.82	10.0	70.0
**Total**	80	13	67	36.00	24.77	8.6	160.0

GPT = group psychotherapy. MPH = Methylphenidate. CM = clinical management. Plac = Placebo.

© 2019 Lam AP et al. *JAMA Network Open.*

eTable 4 Daily Dosage MPH (mg/kg) at T5 by Former Randomized Treatment Groups

	Daily dosage MPH [mg/kg] at T5						
	**Total**	**Missing**	**Total valid**	**Mean**	**SD**	**Minimum**	**Maximum**
GPT + MPH	18	3	15	0.48	0.28	0.1	1.1
GPT + Plac	22	2	20	0.48	0.2	0.1	0.8
CM + MPH	25	4	21	0.46	0.34	0.1	1.5
CM + Plac	15	4	11	0.38	0.27	0.1	1.1
**Total**	80	13	67	0.46	0.27	0.1	1.5

GPT = group psychotherapy. MPH = Methylphenidate. CM = clinical management. Plac = Placebo.

© 2019 Lam AP et al. *JAMA Network Open.*

eTable 5 Primary and Secondary Outcomes at 2.5 Years (T5).

Observed Distribution by Randomized Intervention in the Full Analysis Set (n=419) (Lower score values represent better outcomes except for clinical global impression scale Global Assessment of Effectiveness).

	T1<br>Baseline	T5<br>2.5 years
**CAARS-O:L ADHD Index (range: 0 – 36)**		
GPT + MPH	20.4 ± 5.7 (103)	13.9 ± 5.6 (64)
GPT + Plac	19.8 ± 5.8 (106)	14.2 ± 6.5 (66)
CM + MPH	21.3 ± 5.1 (107)	13.9 ± 6.0 (68)
CM + Plac	20.7 ± 4.9 (103)	15.2 ± 5.5 (53)
**CAARS-O:L Inattention/Memory Problems (range: 0 – 36)**		
GPT + MPH	20.8 ± 6.8 (103)	13.8 ± 6.5 (64)
GPT + Plac	20.3 ± 7.7 (106)	13.6 ± 6.7 (66)
CM + MPH	21.3 ± 6.3 (107)	13.3 ± 6.8 (68)
CM + Plac	20.9 ± 7.1 (103)	14.7 ± 6.6 (53)
**CAARS-O:L Hyperactivity/Restlessness (range: 0 – 36)**		
GPT + MPH	17.8 ± 7.5 (103)	11.9 ± 6.8 (64)
GPT + Plac	18.3 ± 8.0 (106)	13.1 ± 7.6 (66)
CM + MPH	18.3 ± 8.3 (107)	14.0 ± 7.7 (68)
CM + Plac	18.8 ± 7.2 (103)	14.0 ± 7.5 (53)
**CAARS-O:L Impulsivity/Emotional Lability (range: 0 – 36)**		
GPT + MPH	17.8 ± 7.2 (103)	12.4 ± 5.6 (64)
GPT + Plac	18.7 ± 7.5 (106)	12.7 ± 6.8 (66)
CM + MPH	18.7 ± 7.1 (107)	11.7 ± 6.6 (68)
CM + Plac	19.1 ± 6.6 (103)	14.3 ± 6.5 (53)
**CAARS-O:L Problems with Self-Concept (range: 0 – 18)**		
GPT + MPH	10.0 ± 4.7 (103)	8.0 ± 4.8 (64)
GPT + Plac	8.9 ± 4.6 (106)	6.9 ± 4.9 (66)
CM + MPH	10.4 ± 4.5 (107)	7.8 ± 4.6 (68)
CM + Plac	10.2 ± 4.8 (103)	8.5 ± 4.5 (53)
**CAARS-O:L DSM-IV Inattentive Symptoms (range: 0 – 27)**		
GPT + MPH	15.7 ± 5.5 (103)	10.3 ± 4.7 (64)
GPT + Plac	15.9 ± 5.8 (106)	10.3 ± 5.4 (66)
CM + MPH	16.8 ± 4.4 (107)	10.4 ± 4.8 (68)
CM + Plac	16.4 ± 5.2 (103)	11.7 ± 4.9 (53)
**CAARS-O:L DSM-IV Hyperactive-Impulsive Symptoms (range: 0 – 27)**		
GPT + MPH	10.9 ± 5.5 (103)	7.0 ± 4.5 (64)
GPT + Plac	12.0 ± 6.1 (106)	7.9 ± 5.1 (66)
CM + MPH	11.7 ± 5.9 (107)	8.3 ± 5.0 (68)
CM + Plac	12.6 ± 5.7 (103)	8.4 ± 4.8 (53)
**CAARS-O:L DSM-IV ADHD Symptoms Total (range: 0 – 54)**		
GPT + MPH	26.6 ± 9.1 (103)	17.3 ± 7.8 (64)
GPT + Plac	27.9 ± 9.5 (106)	18.2 ± 9.1 (66)
CM + MPH	28.5 ± 8.1 (107)	18.6 ± 8.4 (68)
CM + Plac	29.1 ± 9.0 (103)	20.1 ± 7.4 (53)
**CAARS-O:L Total Score (range: 0 – 198)**		
GPT + MPH	103.6 ± 29.0 (103)	70.5 ± 27.5 (64)
GPT + Plac	104.8 ± 30.1 (106)	72.2 ± 31.2 (66)
CM + MPH	108.7 ± 25.8 (107)	72.7 ± 29.8 (68)
CM + Plac	109.2 ± 27.5 (103)	79.8 ± 26.9 (53)
**CAARS-S:L ADHD Index (range: 0 – 36)**		

© 2019 Lam AP et al. *JAMA Network Open.*

GPT + MPH	20.5 ± 6.3 (101)	14.9 ± 6.1 (60)
GPT + Plac	20.1 ± 5.9 (104)	14.4 ± 6.8 (61)
CM + MPH	21.3 ± 5.5 (105)	15.2 ± 6.2 (69)
CM + Plac	21.5 ± 6.1 (102)	15.8 ± 6.8 (52)
**CAARS-S:L Inattention/Memory Problems (range: 0 – 36)**		
GPT + MPH	20.4 ± 7.3 (101)	14.8 ± 5.8 (60)
GPT + Plac	20.2 ± 7.5 (104)	14.7 ± 6.9 (61)
CM + MPH	21.1 ± 6.7 (105)	14.9 ± 7.5 (69)
CM + Plac	21.9 ± 7.3 (102)	15.7 ± 7.4 (52)
**CAARS-S:L Hyperactivity/Restlessness (range: 0 – 36)**		
GPT + MPH	17.8 ± 7.5 (101)	12.1 ± 7.0 (60)
GPT + Plac	18.4 ± 7.4 (104)	12.4 ± 7.1 (61)
CM + MPH	18.1 ± 7.3 (105)	13.8 ± 7.4 (69)
CM + Plac	19.1 ± 8.0 (102)	14.6 ± 8.5 (52)
**CAARS-S:L Impulsivity/Emotional Lability (range: 0 – 36)**		
GPT + MPH	17.4 ± 7.4 (101)	13.0 ± 5.6 (60)
GPT + Plac	18.7 ± 7.4 (104)	13.8 ± 7.0 (61)
CM + MPH	18.9 ± 6.9 (105)	13.1 ± 7.3 (69)
CM + Plac	20.0 ± 7.1 (102)	14.9 ± 7.4 (52)
**CAARS-S:L Problems with Self-Concept (range: 0 – 18)**		
GPT + MPH	10.4 ± 4.5 (101)	8.7 ± 5.0 (60)
GPT + Plac	9.6 ± 5.2 (104)	7.5 ± 5.0 (61)
CM + MPH	11.3 ± 4.4 (105)	8.5 ± 5.0 (69)
CM + Plac	10.7 ± 4.6 (102)	8.5 ± 4.6 (52)
**CAARS-S:L DSM-IV Inattentive Symptoms (range: 0 – 27)**		
GPT + MPH	15.8 ± 5.3 (101)	10.7 ± 4.6 (60)
GPT + Plac	16.2 ± 5.4 (104)	10.7 ± 5.8 (61)
CM + MPH	16.6 ± 4.8 (105)	11.3 ± 5.0 (69)
CM + Plac	17.0 ± 5.3 (102)	12.1 ± 5.4 (52)
**CAARS-S:L DSM-IV Hyperactive-Impulsive Symptoms (range: 0 – 27)**		
GPT + MPH	11.4 ± 5.8 (101)	7.6 ± 4.6 (60)
GPT + Plac	12.0 ± 5.2 (104)	7.6 ± 5.1 (61)
CM + MPH	12.1 ± 5.8 (105)	8.5 ± 5.3 (69)
CM + Plac	12.8 ± 5.9 (102)	9.4 ± 5.9 (52)
**CAARS-S:L DSM-IV ADHD Symptoms Total (range: 0 – 54)**		
GPT + MPH	27.3 ± 9.5 (101)	18.3 ± 7.9 (60)
GPT + Plac	28.1 ± 8.4 (104)	18.4 ± 9.3 (61)
CM + MPH	28.7 ± 8.6 (105)	19.9 ± 8.7 (69)
CM + Plac	29.8 ± 9.4 (102)	21.5 ± 9.7 (52)
**CAARS-S:L Total Score (range: 0 – 198)**		
GPT + MPH	104.4 ± 31.8 (101)	74.3 ± 27.9 (60)
GPT + Plac	105.9 ± 29.0 (104)	74.6 ± 32.3 (61)
CM + MPH	109.4 ± 27.9 (105)	78.3 ± 30.2 (69)
CM + Plac	113.1 ± 31.0 (102)	83.6 ± 32.5 (52)
**ADHD-DC Total Score (range: 0 – 54)**		
GPT + MPH	29.7 ± 9.6 (102)	19.0 ± 9.1 (63)
GPT + Plac	30.1 ± 9.7 (106)	19.3 ± 10.1 (67)
CM + MPH	30.5 ± 8.3 (106)	20.0 ± 9.6 (69)
CM + Plac	31.5 ± 8.7 (103)	21.7 ± 8.6 (54)
**ADHD-DC Inattention (range: 0 – 27)**		
GPT + MPH	16.4 ± 5.6 (101)	9.9 ± 5.1 (63)
GPT + Plac	16.3 ± 5.6 (106)	10.5 ± 5.4 (67)
CM + MPH	16.6 ± 4.6 (107)	10.2 ± 4.9 (69)

© 2019 Lam AP et al. *JAMA Network Open.*

CM + Plac	17.0 ± 4.6 (103)	11.5 ± 5.3 (52)
**ADHD-DC Hyperactivity/Impulsivity (range: 0 – 27)**		
GPT + MPH	13.3 ± 5.9 (102)	9.0 ± 5.3 (63)
GPT + Plac	13.8 ± 6.4 (106)	8.4 ± 6.0 (65)
CM + MPH	13.9 ± 6.0 (105)	9.8 ± 6.4 (69)
CM + Plac	14.5 ± 5.8 (101)	10.1 ± 5.4 (54)
**ADHD-DC Hyperactivity (range: 0 – 15)**		
GPT + MPH	7.5 ± 3.6 (102)	4.8 ± 3.6 (64)
GPT + Plac	7.6 ± 4.1 (106)	4.4 ± 3.7 (65)
CM + MPH	7.4 ± 3.8 (105)	5.6 ± 4.1 (69)
CM + Plac	7.9 ± 3.5 (102)	5.7 ± 3.8 (54)
**ADHD-DC Impulsivity (range: 0 – 12)**		
GPT + MPH	5.8 ± 3.0 (102)	4.1 ± 2.5 (63)
GPT + Plac	6.2 ± 3.1 (106)	4.2 ± 3.1 (67)
CM + MPH	6.5 ± 2.9 (107)	4.2 ± 3.0 (69)
CM + Plac	6.6 ± 3.0 (102)	4.5 ± 3.1 (54)
**BDI Total Score (range: 0 – 63)**		
GPT + MPH	11.8 ± 8.4 (101)	9.8 ± 8.5 (60)
GPT + Plac	12.3 ± 8.8 (104)	8.1 ± 7.4 (63)
CM + MPH	12.2 ± 7.3 (106)	8.5 ± 8.3 (69)
CM + Plac	14.0 ± 10.0 (100)	9.7 ± 8.9 (53)
**CGI Severity (descriptive numerical evaluation, range: 1=not at all ill – 7=extremely ill)**		
GPT + MPH	4.7 ± 1.0 (103)	3.2 ± 1.2 (63)
GPT + Plac	4.6 ± 1.0 (104)	3.6 ± 1.2 (67)
CM + MPH	4.7 ± 0.9 (106)	3.2 ± 1.3 (69)
CM + Plac	4.7 ± 0.9 (101)	3.8 ± 1.1 (53)
**CGI Global Change (descriptive numerical evaluation, range: 1=very much improved – 7=very much worse)**		
GPT + MPH	–	2.7 ± 0.9 (64)
GPT + Plac		2.8 ± 1.1 (67)
CM + MPH		2.9 ± 1.1 (69)
CM + Plac		2.9 ± 1.0 (54)
**CGI Global Assessment of Effectiveness (descriptive numerical evaluation, range: 1=minimal – 4=very good)**		
GPT + MPH	–	2.5 ± 1.0 (64)
GPT + Plac		2.4 ± 1.0 (67)
CM + MPH		2.3 ± 1.0 (69)
CM + Plac		2.0 ± 1.1 (54)

Data are mean ± SD (n). GPT = group psychotherapy. MPH = Methylphenidate. CM = clinical management. Plac = Placebo. ADHD = Attention Deficit Hyperactivity Disorder. CAARS-O:L = Conners' Adult ADHD Rating Scale - Observer-Rating Scale, Long Version. DSM = Diagnostic and Statistical Manual of Mental Disorders. CAARS-S:L = Conners' Adult ADHD Rating Scale - Self-Rating Scale, Long Version. DC = Diagnostic Checklist. CGI = Clinical Global Impression Scale.

© 2019 Lam AP et al. *JAMA Network Open.*

eTable 6 Stability T5-T4

Change from End of Study Period (T4) to Follow-Up (T5) 2.5 Years After Baseline (T1) for Primary and Major Secondary Outcomes in the Full Analysis Set, by Randomized Intervention. Least Squares Means from Longitudinal Linear Regression Analysis Adjusted for Baseline and Centre in Patients Measured at T4 or T5. Lower score values represent better outcomes.

	T5 – T4 mean (95% CI)	p-value
**CAARS-O:L ADHD Index (range: 0-36; primary outcome scale)**		
GPT	-0.6 (-1.5 to 0.4)	0.26
CM	-0.3 (-1.3 to 0.8)	0.63
MPH	-0.5 (-1.5 to 0.4)	0.28
Plac	-0.3 (-1.4 to 0.8)	0.60
**CAARS-O:L Inattention/Memory Problems (range: 0 – 36)**		
GPT	-0.5 (-1.5 to 0.6)	0.39
CM	-0.5 (-1.6 to 0.7)	0.40
MPH	-0.7 (-1.7 to 0.3)	0.19
Plac	-0.3 (-1.4 to 0.9)	0.68
**CAARS-O:L Hyperactivity/Restlessness (range: 0 – 36)**		
GPT	-0.9 (-1.9 to 0.2)	0.10
CM	0.3 (-0.9 to 1.5)	0.62
MPH	-0.3 (-1.4 to 0.7)	0.57
Plac	-0.3 (-1.4 to 0.9)	0.64
**CAARS-O:L Impulsivity/Emotional Lability (range: 0 – 36)**		
GPT	-0.5 (-1.5 to 0.6)	0.41
CM	-0.3 (-1.5 to 0.9)	0.61
MPH	-0.6 (-1.6 to 0.5)	0.31
Plac	-0.2 (-1.4 to 1.0)	0.74
**CAARS-O:L Problems with Self-Concept (range: 0 – 18)**		
GPT	-0.5 (-1.5 to 0.6)	0.77
CM	-0.3 (-1.5 to 0.9)	0.82
MPH	-0.6 (-1.6 to 0.5)	0.47
Plac	-0.2 (-1.4 to 1.0)	0.87
**CAARS-S:L ADHD Index (range: 0-36)**		
GPT	-0.4 (-1.4 to 0.6)	0.44
CM	-0.5 (-1.6 to 0.6)	0.40
MPH	0.1 (-0.9 to 1.1)	0.81
Plac	-1.0 (-2.1 to 0.1)	0.08
**BDI Total Score (range: 0 – 63)**		
GPT	1.3 (0.1 to 2.4)	0.03
CM	0.4 (-0.9 to 1.6)	0.58
MPH	1.0 (-0.1 to 2.1)	0.08
Plac	0.6 (-0.6 to 1.9)	0.32

CI = confidence interval. ADHD = Attention Deficit Hyperactivity Disorder. GPT = group psychotherapy. MPH = Methylphenidate. CM = clinical management. Plac = Placebo. CAARS-O:L = Conners' Adult ADHD Rating Scale – Observer-Rating Scale, Long Version. CAARS-S:L = Conners' Adult ADHD Rating Scale - Self-rating Scale, Long Version. BDI = Beck Depressions Inventory (BDI-II).

© 2019 Lam AP et al. *JAMA Network Open.*

eTable 7 Self-Rated CAARS ADHD Index and BDI Total Score.

Conners' Adult ADHD Rating Scale – Self-Rating Scale, Long Version, and Beck Depressions Inventory at Follow-Up (T5) 2.5 Years After Baseline (T1), by Randomized Intervention. Regression Analysis Adjusted for Baseline and Centre (Least squares means from Linear Regression). Lower score values represent better outcomes.

	**T1 all-group mean**	**T5 mean (95% CI)**	**T5 – T1 mean (n)**
**CAARS-S:L ADHD Index (range: 0-36)**			
GPT	20.8	14.6 (13.6 to 15.6)	-6.2 (119)
CM	20.8	15.5 (14.4 to 16.5)	-5.4 (121)
MPH	20.8	14.7 (13.7 to 15.7)	-6.1 (128)
Plac	20.8	15.4 (14.3 to 16.4)	-5.5 (112)
Difference, GPT vs CM		-0.9 (-2.3 to 0.6)	
p-value		0.24	
Difference, MPH vs Plac		-0.7 (-2.1 to 0.8)	
p-value		0.36	
**BDI Total Score (range: 0 – 63)**			
GPT	12.5	9.2 (8.0 to 10.4)	-3.3 (122)
CM	12.5	9.2 (8.0 to 10.5)	-3.3 (120)
MPH	12.5	9.3 (8.1 to 10.5)	-3.2 (129)
Plac	12.5	9.1 (7.9 to 10.3)	-3.4 (113)
Difference, GPT vs CM		-0.1 (-1.7 to 1.6)	
p-value		0.94	
Difference, MPH vs Plac		0.2 (-1.5 to 1.9)	
p-value		0.80	

CI = confidence interval. ADHD = Attention Deficit Hyperactivity Disorder. GPT = group psychotherapy. MPH = Methylphenidate. CM = clinical management. Plac = Placebo. CAARS-O:L = Conners' Adult ADHD Rating Scale - Observer-Rating Scale, Long Version. CAARS-S:L = Conners' Adult ADHD Rating Scale - Self-rating Scale, Long Version. BDI = Beck Depressions Inventory (BDI-II).

© 2019 Lam AP et al. *JAMA Network Open.*

eTable 8 Major Secondary Outcomes in the Full Analysis Set at Follow-Up (T5) 2.5 Years After Baseline (T1).

By Randomized Intervention, Regression Analysis Adjusted for Baseline and Center (Least squares means from Linear Regression. Lower score values represent better outcomes.

	T1 all-group mean	T5 mean (95% CI)	T5 – T1 mean (n)
**CAARS-S:L Inattention/Memory Problems (range: 0-36)**			
GPT	20.9	14.6 (13.6 to 15.6)	-6.3 (119)
CM	20.9	15.3 (14.2 to 16.3)	-5.6 (121)
MPH	20.9	14.6 (13.6 to 15.6)	-6.3 (128)
Plac	20.9	15.3 (14.2 to 16.4)	-5.6 (112)
Difference, GPT vs CM		-0.7 (-2.1 to 0.8)	
p-value		0.36	
Difference, MPH vs Plac		-0.7 (-2.2 to 0.7)	
p-value		0.33	
**CAARS-S:L Hyperactivity/Restlessness (range: 0-36)**			
GPT	18.4	12.6 (11.6 to 13.7)	-5.7 (119)
CM	18.4	14.0 (12.9 to 15.0)	-4.4 (121)
MPH	18.4	12.9 (11.8 to 13.9)	-5.5 (128)
Plac	18.4	13.7 (12.7 to 14.8)	-4.6 (112)
Difference, GPT vs CM		-1.3 (-2.8 to 0.1)	
p-value		0.08	
Difference, MPH vs Plac		-0.9 (-2.3 to 0.6)	
p-value		0.23	
**CAARS-S:L Impulsivity/Emotional Lability (range: 0-36)**			
GPT	18.7	13.5 (12.4 to 14.5)	-5.3 (119)
CM	18.7	14.1 (13.1 to 15.2)	-4.6 (121)
MPH	18.7	13.4 (12.4 to 14.5)	-5.3 (128)
Plac	18.7	14.2 (13.1 to 15.2)	-4.6 (112)
Difference, GPT vs CM		-0.7 (-2.2 to 0.8)	
p-value		0.37	
Difference, MPH vs Plac		-0.7 (-2.2 to 0.8)	
p-value		0.35	
**CAARS-S:L Problems with Self-Concept (range: 0-18)**			
GPT	10.5	8.0 (7.3 to 8.7)	-2.5 (119)
CM	10.5	8.2 (7.5 to 8.8)	-2.4 (121)
MPH	10.5	7.9 (7.2 to 8.6)	-2.6 (128)
Plac	10.5	8.3 (7.6 to 9.0)	-2.2 (112)
Difference, GPT vs CM		-0.1 (-1.1 to 0.8)	
p-value		0.81	
Difference, MPH vs Plac		-0.4 (-1.4 to 0.6)	
p-value		0.41	
**ADHD-DC Total Score (range: 0-54)**			
GPT	30.5	19.6 (18.1 to 21.1)	-10.9 (130)
CM	30.5	21.4 (19.8 to 22.9)	-9.1 (122)
MPH	30.5	20.0 (18.5 to 21.5)	-10.5 (131)
Plac	30.5	21.0 (19.5 to 22.5)	-9.5 (121)
Difference, GPT vs CM		-1.8 (-3.9 to 0.3)	
p-value		0.10	

© 2019 Lam AP et al. *JAMA Network Open.*

Difference, MPH vs Plac		-1.0 (-3.1 to 1.1)	
p-value		0.34	
**ADHD-DC Inattention (range: 0-27)**			
GPT	16.6	10.4 (9.6 to 11.2)	-6.2 (130)
CM	16.6	11.3 (10.4 to 12.2)	-5.2 (121)
MPH	16.6	10.4 (9.6 to 11.3)	-6.1 (132)
Plac	16.6	11.3 (10.4 to 12.2)	-5.3 (119)
Difference, GPT vs CM		-0.9 (-2.1 to 0.3)	
p-value		0.12	
Difference, MPH vs Plac		-0.8 (-2.0 to 0.4)	
p-value		0.17	
**ADHD-DC Hyperactivity/Impulsivity (range: 0-27)**			
GPT	13.9	9.1 (8.2 to 9.9)	-4.8 (128)
CM	13.9	10.0 (9.1 to 10.9)	-3.8 (120)
MPH	13.9	9.5 (8.7 to 10.4)	-4.3 (130)
Plac	13.9	9.6 (8.7 to 10.4)	-4.3 (118)
Difference, GPT vs CM		-1.0 (-2.2 to 0.3)	
p-value		0.12	
Difference, MPH vs Plac		-0.0 (-1.2 to 1.2)	
p-value		0.97	
**ADHD-DC Hyperactivity (range: 0-15)**			
GPT	7.6	4.6 (4.1 to 5.2)	-3.0 (129)
CM	7.6	5.5 (4.9 to 6.1)	-2.2 (121)
MPH	7.6	5.1 (4.5 to 5.6)	-2.5 (131)
Plac	7.6	5.0 (4.4 to 5.6)	-2.6 (119)
Difference, GPT vs CM		-0.8 (-1.6 to -0.0)	
p-value		0.048	
Difference, MPH vs Plac		0.1 (-0.7 to 0.9)	
p-value		0.88	
**ADHD-DC Impulsivity (range: 0-12)**			
GPT	6.3	4.5 (4.0 to 4.9)	-1.8 (130)
CM	6.3	4.5 (4.1 to 5.0)	-1.7 (122)
MPH	6.3	4.4 (4.0 to 4.9)	-1.8 (132)
Plac	6.3	4.6 (4.1 to 5.1)	-1.7 (120)
Difference, GPT vs CM		-0.1 (-0.7 to 0.5)	
p-value		0.79	
Difference, MPH vs Plac		-0.2 (-0.8 to 0.4)	
p-value		0.58	

CI = confidence interval. ADHD = Attention Deficit Hyperactivity Disorder. GPT = group psychotherapy. MPH = Methylphenidate. CM = clinical management. Plac = Placebo. CAARS-O:L = Conners' Adult ADHD Rating Scale - Observer-Rating Scale, Long Version. CAARS-S:L = Conners' Adult ADHD Rating Scale - Self-rating Scale, Long Version. BDI = Beck Depressions Inventory (BDI-II).

© 2019 Lam AP et al. *JAMA Network Open.*
