# Supplementary material for: Long-term Effects of Multimodal Treatment on Adult Attention-Deficit/Hyperactivity Disorder Symptoms: Follow-up Analysis of the COMPAS Trial
Source: JAMA Netw Open. 2019 May 31;2(5):e194980. doi: 10.1001/jamanetworkopen.2019.4980 (PMC6547099; doi:10.1001/jamanetworkopen.2019.4980)
Supplement: Supplement 3. — Data Sharing Statement [file jamanetwopen-2-e194980-s003.pdf]

Data Sharing Statement

Lam. Long-term Effects of Multimodal Treatment on Adult Attention-Deficit/Hyperactivity Disorder Symptoms. *JAMA Netw Open*. Published May 31, 2019. 10.1001/jamanetworkopen.2019.4980

Data
Data available: Yes
Data types: Data dictionary, Other (please specify)
Additional Information: Randomised Group Data
How to access data: Results of randomised groups will be available in the trial registers (BfArM, Federal Institute of Medication and Medical Advices)
When available: With publication

Supporting Documents
Document types: Statistical/analytic code, Informed consent form
How to access documents: Upon request (note: informed consent in German language). The full trial protocol appears in the supplement, is available from the corresponding author on request and is accessible in the following website: https://www.ukbonn.de/42256BC8002AF3E7/direct/compas. The statistical analysis plan (SAP) appears in the supplement and can be downloaded from https://www.ukbonn.de/42256BC8002AF3E7/direct/compas.
When available: With publication

Additional Information
Who can access the data: researchers whose proposed use of the data has been approved
Types of analyses: for specified purpose
Mechanisms of data availability: after approval of a proposal and with agreement
